# Determinants of Health-Related Quality of Life After Acute Coronary Syndromes: A Systematic Review

**DOI:** 10.3390/healthcare14101292

**Published:** 2026-05-09

**Authors:** Panagiota Peleka, Olympia Konstantakopoulou, Aglaia Katsiroumpa, Maria Katharaki, Olga Siskou, Daphne Kaitelidou, Petros Galanis

**Affiliations:** 1 Faculty of Nursing, National and Kapodistrian University of Athens, 15773 Athens, Greece; gpeleka@nurs.uoa.gr (P.P.); olykonstant@nurs.uoa.gr (O.K.); aglaiakat@nurs.uoa.gr (A.K.); mkatharaki@nurs.uoa.gr (M.K.); dkaitelid@nurs.uoa.gr (D.K.); 2 Department of Tourism Studies, University of Piraeus, 18534 Piraeus, Greece; olsiskou@webmail.unipi.gr

**Keywords:** determinants, health, quality of life, acute coronary syndromes, patients, systematic review

## Abstract

**Highlights:**

**What are the main findings?**

- Health-related quality of life after acute coronary syndromes is determined by multiple factors, with sex, age and depression being among the most frequent.
- Comorbidity, lack of social support and living alone are associated with significant quality-of-life decline, while a higher baseline quality of life can be a major protective factor for long-term recovery.

**What are the implications of the main findings?**

- Healthcare professionals should prioritize early mental health screening for high-risk patients.
- Targeted interventions and individualized support are essential to optimize modifiable factors, thereby improving patient outcomes and overall quality of care.

**Abstract:**

Background/Objectives: Rapid advancements in reperfusion strategies and optimized medical therapies have significantly improved survival among patients with acute coronary syndromes (ACS). However, a large population still lives with the chronic consequences of the disease, including impaired post-discharge health-related quality of life (HRQOL). This systematic review aimed to identify and summarize the existing evidence regarding the determinants of HRQOL among patients with ACS. Methods: A comprehensive search following Preferred Reporting Items for Systematic Reviews and Meta-Analyses (PRISMA) guidelines was performed in PubMed, Scopus, Web of Science and Science Direct until 20 July 2025. The review protocol was registered with PROSPERO (ID CRD420251157478). Results: From an initial 12,280 articles, 55 were selected. Forty-two studies used a cohort design and thirteen were cross-sectional. All studies were published between 2000 and 2025. Most of the studies were conducted in Europe and America. Common predictors of improved HRQOL were better baseline quality of life, previous exercise behavior, sense of coherence, coping strategies, revascularization during index hospitalization, time passed after ACS and diagnosis of STEMI. The most common factors that worsened HRQOL were female sex, lower educational and financial status, being unemployed, depression, anxiety, the presence of comorbidities and lower social support including having no partner. Conclusions: The findings highlight that post-ACS HRQOL is determined by multiple factors including demographic, clinical and psychological factors. Clinical practice should therefore focus on targeted strategies that optimize modifiable factors, prioritizing early psychological screening and individualized support for high-risk groups, to effectively improve HRQOL and overall quality of care.

## 1. Introduction

Over the past few decades, rapid advancements in reperfusion strategies and optimized medical therapies have significantly improved survival among patients with acute coronary syndromes (ACS) [1]. However, this increase in survival had led to a larger population of patients living with the chronic consequences of the disease. Despite optimal medical management, many ACS survivors experience persistent physical limitations, psychological distress and social challenges that significantly impact their health-related quality of life (HRQoL) [2,3,4,5].

To effectively support patients and improve long-term outcomes, healthcare systems should focus more on optimizing post-discharge HRQOL. This requires a clear understanding of factors that influence the patients’ perceptions of quality of life. While numerous primary studies have examined multiple unique determinants (including clinical, psychosocial, demographic, socioeconomic, etc.), the evidence remains fragmented. To the best of our knowledge, no systematic review or meta-analysis has comprehensively synthesized the multifaceted determinants of HRQOL specifically within ACS populations. Previous systematic reviews have predominantly focused on the impact of specific interventions on HRQOL (e.g., exercise-based cardiac rehabilitation [6], coronary revascularization [7,8]), included broader patient populations (e.g., coronary artery disease [9,10]) or focused on individual determinants such as marital/partner status [11]. Moreover, a previous systematic review by Simpson and Pilote (2003) [12] and a literature review by Kang et al. (2017) [13] assessing determinants of HRQOL focused exclusively on myocardial infarction patients. Furthermore, earlier reviews do not adequately capture developments in the most recent literature, which includes advancements in pharmacological treatments, interventional cardiology therapies and patient-centered care models. Consequently, current evidence remains limited with respect to understanding how these diverse factors interact in shaping HRQOL in the contemporary era of ACS management.

Moreover, different studies often utilize different assessment tools, ranging from generic instruments like Short Form-36 [4] or EQ-5D [5] to disease-specific questionnaires like the Seattle Angina Questionnaire (SAQ) [14] or the Macnew Heart Disease HRQOL Questionnaire [15], and report results in varied populations at different follow-up time points. This heterogeneity makes it challenging for healthcare professionals and policy makers to synthesize a clear, evidence-based picture of the key determinants of HRQOL post-ACS.

Therefore, a comprehensive synthesis of the literature is needed in this research field. Thus, the aim of this systematic review is to identify and summarize the existing evidence regarding the determinants of HRQOL after ACS and to provide a clear foundation for future clinical practice and research.

## 2. Materials and Methods

### 2.1. Data Sources and Search Strategy

A systematic review was conducted following the Preferred Reporting Items for Systematic Reviews and Meta-Analysis (PRISMA) guidelines [16]. Pubmed, Scopus, Web of Science and Science Direct were searched until 20 July 2025. The following strategy was used: (determinant* OR predict* OR impact OR relationship OR influenc* OR “risk factor*” OR “factor* affecting” OR factor* OR exposure* OR associat* OR “influencing factor*” OR age OR anxiety OR gender OR sex OR “sense of coherence” OR depression OR “socioeconomic factor*” OR “social class” OR “social support” OR race OR diabetes OR “living alone” OR “self-efficacy” OR “coping strategies” OR “type-D personality” OR “patient sex” OR men OR women OR frailty) AND (“health related quality of life” OR “quality of life” OR QOL OR HRQOL OR “patient reported outcome*” OR PROMs OR “health status” OR “functional status” OR “health outcome*” OR “well-being” OR “patient reported quality of life” OR outcome*) AND (“acute myocardial infarction” OR AMI OR MI OR STEMI OR NSTEMI OR “ST-elevation” OR “non-ST-elevation” OR “myocardial ischemia” OR “myocardial infarction” OR “cardiac infarction” OR “coronary thrombosis” OR “coronary occlusion” OR “coronary event” OR “heart attack” OR “acute myocardial syndrome*” OR “acute coronary syndrome*” OR “post-myocardial infarction”). The review protocol was registered with PROSPERO (ID CRD420251157478). Due to the high heterogeneity among several aspects of studies, we cannot perform a meta-analysis. In particular, the studies varied considerably with respect to HRQOL measurement instruments, study designs, patient populations, and follow-up durations. Multiple generic and disease-specific HRQOL tools were used across studies, often capturing different conceptual domains and employing non-comparable scoring systems. In addition, follow-up time points ranged from early post-discharge assessments to long-term evaluations, further complicating direct comparison of outcomes. Beyond outcome measurement, heterogeneity was also present in study designs and populations, including differences in clinical settings, baseline risk profiles, and analytical approaches to identifying determinants of HRQOL.

### 2.2. Selection and Eligibility Criteria

The study selection process was conducted by two independent authors applying three consecutive steps: duplicate removal, title and abstract screening and reading the full-text articles. Two authors selected the studies independently and when any uncertainties regarding article inclusion occurred it was resolved by a third senior author. Reference lists of all relevant articles were screened to identify additional studies. The population of interest was patients with ACS, and the outcome was their HRQOL. Thus, this systematic review included studies that evaluate determinants of HRQOL among patients with ACS. Studies were also eligible if they were published in English and they measured quality of life with a valid and reliable tool.

To ensure the generalizability of the results, the study excluded articles that limited their analysis to specific demographic subgroups, such as a single sex or a specific age category (e.g., only the elderly). Similarly, studies focusing only on one type of ACS (e.g., only STEMI) or included participants with one same clinical characteristic (e.g., diabetes) were also excluded. Moreover, studies that systematically excluded patients with known determinants of HRQOL, such as smoking and diabetes, were not included in this review. Furthermore, we excluded studies including patients treated with a single treatment modality (e.g., only percutaneous coronary intervention or only medication), or studies with patients participating in cardiac rehabilitation programs. Research evaluating the impact of specific post-ACS interventions, such as counseling, exercise protocols, or specific drug therapies, were also excluded, as were studies comparing ACS patients to healthy control groups. Studies were also rejected if they included mixed patient categories (e.g., stroke and ACS patients) without a clear distinction between the patients’ results, or if they did not report a fixed time interval between the ACS event and the quality-of-life assessment (participants are evaluated at different time points post-ACS). Regarding the study design, we excluded all studies that were not original research articles, such as posters, letters to the editor, protocols, reviews, editorials and expert opinions.

### 2.3. Data Extraction and Risk of Bias Assessment

Data extraction was performed by three independent authors following a predefined protocol. A standardized data extraction form was developed a priori and piloted on a sample of five included studies to ensure consistency and clarity. For each study, two reviewers independently extracted data, including study characteristics, population details, outcomes, and key findings. Extracted data were then compared, and discrepancies were resolved through discussion and consensus. In cases where disagreements persisted, a third reviewer was consulted for final adjudication. Data extraction included the following: authors, continent, country, data collection time, sample size, age, sex (%), study design, sampling method, response rate and predictors of HRQOL.

We assessed the risk of bias using the Joanna Briggs institute critical appraisal tools for cross-sectional and cohort studies [17]. Each tool has eight and eleven criteria accordingly and each item has four response options. Items were rated as “Yes” if the criteria were clearly identified, as “No” if the criteria were not identified and as “Unclear” when the criteria were not clearly identified in the article. “Not applicable” was selected when a specific criterion did not apply to the study. Studies were categorized as having a “low”, “moderate” or “high” risk of bias, based on the percentage of the “Yes” responses.

## 3. Results

### 3.1. Identification and Selection of Studies

**Figure 1:**
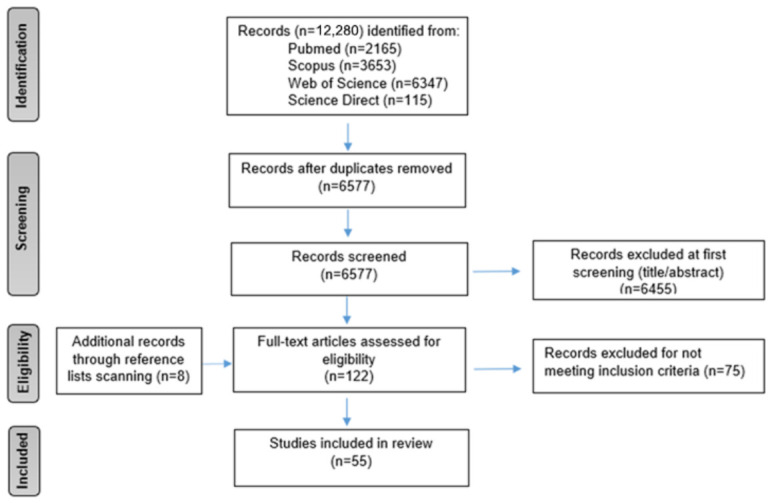
PRISMA flowchart of article selection.

A total of 12,280 articles were identified through database searches. After removing duplicate records, 6577 unique entries remained. Following the initial screening of titles and abstracts, 6455 records were excluded, resulting in 122 potentially relevant articles. Eight additional studies were found via reference list screening, resulting in 130 full-text articles. These records were then independently assessed for eligibility and 75 were excluded as they did not meet the inclusion criteria. The most common reasons for exclusion were studies with patients participating in cardiac rehabilitation programs (n = 16) and studies evaluating quality of life at different time points post-ACS among their participants (n = 13). As a result, 55 studies were included in the final systematic review. The PRISMA flowchart of our systematic review is shown in Figure 1.

### 3.2. Characteristics of the Studies

We found 55 articles in which patient volume ranged from 36 to 2498 participants. The main characteristics of the studies included in this review are presented in Table 1. Twenty-two studies were conducted in Europe, twenty-one in America, eleven in Asia and one in Africa. The most common countries were the USA (17 studies), the UK (seven studies), Sweden (seven studies), Canada (four studies), Spain (three studies), South Korea and Bosnia and Herzegovina (two studies each). Forty-two studies used a cohort design while thirteen were cross-sectional. Data collection time among studies ranged from November 1994 to December 2020. In almost all studies (*n* = 53), males represented the majority of the study population, except in one study where males accounted for the same percentage as women [18] and another one where black male patients accounted 49% of the total black population [19]. The mean age of the participants ranged from 51.9 to 72.2 years. Forty-six studies used a convenience sample technique, while nine used a random sampling technique.

### 3.3. Risk of Bias Assessment

Supplementary Table S1 provides a detailed risk of bias assessment for the included studies. Overall, the cohort studies demonstrated a low risk of bias. However, the most prevalent issues identified were the insufficient reporting of reasons for loss to follow-up and lack of clear strategies for managing incomplete follow-up. Similarly, the risk of bias was low across all cross-sectional studies.

### 3.4. Determinants of HRQOL

This study identified 87 different determinants of HRQOL comprising nine broad categories: demographic factors, socioeconomic factors, patient behaviors, psychological factors, personality traits and coping strategies, baseline quality of life, health system factors, clinical factors and factors related to ACS, risk factors for ACS and patient comorbidities. The most frequently cited determinants were depression (25 studies), sex (23 studies), age (19 studies), anxiety (eight studies), presence of multiple comorbidities (six studies) and baseline quality of life (six studies). A detailed list of determinants and the number of studies that examine each of them are presented in Supplementary Table S2.

The associations between determinants and HRQOL are summarized in Supplementary Table S3, with detailed results, including statistical findings, provided in Supplementary Table S4.

#### 3.4.1. Demographic Factors

Regarding demographic factors, female sex was the most common determinant negatively associated with HRQOL. Older age was identified as a predictor of either worse or better across several studies. Additionally, non-Caucasian versus Caucasian race was more frequently positively associated with HRQOL.

#### 3.4.2. Socioeconomic Factors

Among socioeconomic determinants, a lower educational level was identified as a predictor of worse HRQOL. Similarly, being unemployed or being on sick leave at the time of the ACS event were predictors of poorer HRQOL. Lower financial status was also negatively associated with HRQOL.

Conversely, common predictors of improved HRQOL (positive association) included greater social support and not living alone. Additionally, having a partner was positively associated with HRQOL while being single or a widower strongly predicted worse outcomes.

#### 3.4.3. Patient Behaviors

Analysis of patients’ behaviors revealed that both the previous frequency and duration of exercise, as well as generally engaging in heart-healthy physical activity were associated with better HRQOL. Conversely, smoking habits and a history of alcohol or substance abuse were linked to worse HRQOL. Furthermore, low adherence to prescribed medications was identified as a predictor of poorer HRQOL outcomes.

#### 3.4.4. Psychological Factors

As far as psychological factors are concerned, depression was consistently associated with worse HRQOL. Similarly, anxiety was linked to poorer HRQOL outcomes. Other determinants negatively impacting HRQOL included fatigue, disturbed sleep and higher perceived stress.

#### 3.4.5. Personality Traits and Coping Mechanisms

Within the domain of personality traits and coping mechanisms, a strong sense of coherence was consistently associated with better HRQOL. Similarly, optimism was identified as a predictor of improved HRQOL. Conversely, the negative affect characteristic of a type-D personality was linked to worse HRQOL outcomes.

The impact of coping strategies varied significantly depending on the specific mechanism utilized. Strategies such as problem-focused coping, emotion-focused coping, task-oriented coping, not using avoidance coping and minimization were all positively associated with HRQOL. In contrast, adopting a fatalistic coping strategy was found to be a predictor of poorer HRQOL.

#### 3.4.6. Clinical Factors and Factors Related to Acute Coronary Syndromes

An analysis of clinical factors demonstrated that a higher Left Ventricular Ejection Fraction (LVEF) was associated with better HRQOL. Revascularization in the post-discharge interim was also linked to improved HRQOL. The passage of time following an AMI was associated with better HRQOL outcomes at both 3-month and 6-month follow-ups. Conversely, greater severity of infarction as measured by higher Peel Index scores and higher Killip class was associated with poorer HRQOL. Furthermore, a prior Coronary Artery Bypass Graft (CABG) and a previous acute myocardial infarction (AMI) were identified as predictors of worse HRQOL. Patients diagnosed with unstable angina experienced worse HRQOL compared to those with an AMI, while being diagnosed with STEMI was associated with improved HRQOL compared to NSTEMI.

A longer length of hospital stay was reported as a predictor of either worse or improved HRQOL. Rehospitalization after the index event and the presence of cardiac symptoms, particularly the frequency of angina, strongly predicted worse HRQOL.

#### 3.4.7. Risk Factors for ACS and Patient Comorbidities

With respect to patient comorbidities, the presence of more comorbid conditions was consistently associated with worse HRQOL. A diagnosis of diabetes was identified as a predictor of worse HRQOL in several studies but was associated with better HRQOL in others.

Other cardiovascular conditions were primarily linked to poorer outcomes. A previous stroke and a history of heart failure were significant predictors of worse HRQOL. Similarly, the presence of coronary heart disease was negatively associated with HRQOL. Finally, hypertension was reported as a factor that worsened HRQOL across multiple studies.

#### 3.4.8. Previous Quality of Life and Dimensions

Baseline quality of life and its specific dimensions were identified as strong predictors of follow-up outcomes. Specifically, a better previous overall HRQOL was positively associated with improved HRQOL after ACS. Similarly, better baseline mental health and better baseline physical health were both strongly correlated with enhanced HRQOL.

#### 3.4.9. Health System Factor

The literature search identified only a single determinant related to health system accessibility. Specifically, poorer access to care was found to be a significant predictor of lower HRQOL.

## 4. Discussion

This systematic review identified multiple determinants of HRQOL, including demographic, clinical, socioeconomic, and psychological factors, patient behaviors, personality traits and coping mechanisms, some patient risk factors and comorbidities, factors related to previous quality of life and one health system factor. As assessed in our quality appraisal, all the studies included demonstrated a low risk of bias. Consequently, all our findings are consistently driven by high-quality evidence.

### 4.1. Demographic Factors

Regarding demographic variables, all of which were non-modifiable, sex and age appeared to be the most frequent determinants of HRQOL among patients with ACS. The vast majority of studies indicate that female patients experience significantly worse HRQOL outcomes compared to their male counterparts [3,18,20,21,22,24,25,27,31,34,35,37,40,41,42,45,52,53,55,56,57,62]. Women were found to report lower physical functioning, poorer mental health and greater deficits in social functioning compared to men at various time points post event. Critically, this disparity may be explained by the pressure that women face to quickly resume multiple societal roles, such as balancing professional duties with family caregiving, which often leave them with less time for adequate physical recovery and self-care. However, Džubur et al. (2022) [64] identified that women under 65 years reported better general health compared to men. The influence of age was also identified in many studies, as advancing age was negatively associated with many dimensions of HRQOL [24,33,34,40,49,52,55,56,57,60]. Older patients were frequently reported to have worse physical functioning and greater bodily pain than their younger individuals, possibly due to the natural physiological decline and loss of functional reserves. In contrast, the relationship between age and psychosocial well-being demonstrated a reverse trend. Several studies found that older age was a predictor of higher mental health, possibly showing better emotional resilience or adaptation than younger ACS survivors [21,26,53]. This highlights that older adults may adjust their health expectations downwards, resulting in higher mental satisfaction despite physical limitations. Racial disparities were also identified as a determinant of HRQOL outcomes following ACS. The available evidence highlights a significant gap between racial groups, specifically indicating that black patients experience worse HRQOL compared to white ones [19,43]. Some other studies identified Caucasian race as a predictor of physical limitation compared to non-Caucasian patients [26,35]. These racial disparities may often reflect deeper, modifiable systemic issues, such as ingrained socioeconomic inequalities and varying access to post-discharge cardiac rehabilitation.

### 4.2. Socioeconomic Factors

Socioeconomic determinants also had an influence on patients’ HRQOL after ACS, including both less modifiable and highly modifiable factors. Regarding the less modifiable factors, financial status emerged as a powerful determinant of post-ACS quality of life, exhibiting that higher annual incomes were linked to better health outcomes [2,5,38,46,47,63]. Significantly, the subjective perception of financial strain also played a critical role, as patients who perceived their financial status as poor reported worse HRQOL at both baseline and follow-up assessments. Educational attainment was also a strong determinant of HRQOL as patients with higher educational backgrounds reported significantly better overall HRQOL [2,5,41,55,56,63]. Higher education likely correlated with greater health literacy, empowering patients to better adhere to medication and lifestyle changes.

On the other hand, several socioeconomic factors can be more modifiable, using post-discharge interventions, rehabilitation programs and social policy support. First, employment status was identified as a significant predictor of HRQOL. Being employed at the time of an ACS event demonstrated a protective effect, as these patients reported better physical health status compared to those being unemployed or on sick leave, even after adjusting for age and sex [22,24,25,33]. Returning to work serves not only as a source of income, but also restores a sense of normalcy and social integration. Social support also had a positive correlation with HRQOL. Evidence suggests that higher levels of perceived social support act as a protective factor, leading to better overall quality of life and greater treatment satisfaction [14,45,47,60,65]. From a critical perspective, social support may help mitigate acute psychological stress, while also enhancing individuals’ ability to consistently follow required lifestyle modifications. Closely linked to the concept of social support, patient partner status and living alone were consistently identified as factors that determine HRQOL. Specifically, having a partner [22,25,57] and not living alone [22,25,48] had a positive association with better health outcomes, which suggests that they were possibly provided with essential emotional and social support compared with patients who were living alone and did not have a partner.

### 4.3. Patient Behaviors

Pre-existing and ongoing health behaviors represent highly modifiable determinants that offer direct pathways for clinical intervention. Physical activity habits were found to play a protective role in post-ACS HRQOL. A consistent positive association was observed between exercise and better health outcomes, with benefits linked specifically to the frequency and duration of physical activity [22,25,57]. Exercise may provide cardiovascular benefits at a physical level, while also reducing patients’ anxiety at a psychological level. In contrast, previous smoking status was identified as a negative determinant of HRQOL in ACS patients. Patients who were current or past smokers reported significantly worse physical quality of life [15,33]. Along with smoking, a history of alcohol and substance abuse also predicted worse physical functioning [35]. Such addictive behaviors may be indicators of a broader pattern of self-neglect, making these individuals much less likely to maintain healthy cardiovascular diets or attend crucial follow-up appointments. Another patient behavior that was linked with HRQOL was medication adherence. Patients who demonstrated lower overall compliance with their drug therapy and specifically low compliance with lipid-lowering medications reported lower mental health [28]. This finding may suggest a potential bidirectional relationship where poor mental health may hinder patients’ medication adherence and conversely failure to adhere to medical advice and treatment protocols may reflect a broader problem with self-care and psychological well-being.

### 4.4. Psychological Factors

Psychological factors can be fully modifiable, as they can be highly responsive to targeted strategies. Among the psychological determinants of HRQOL (and among all determinants), depression was found to be the most significant and consistent negative predictor. This review identifies an inverse correlation between depressive symptoms and health outcomes where higher levels of depression are associated with poorer quality of life [2,4,20,22,23,24,25,26,27,29,31,32,33,34,40,41,46,53,55,56,60,61,63,64,65]. While this negative impact is broad, affecting both mental and physical domains, the prognostic value of depression seems to be long-lasting. Depressive symptoms assessed during hospitalization or shortly after the event (e.g., at one week or one month) were found to be strong independent predictors of impaired physical and mental functioning at 3 months, 6 months, one year and even up to five years post-ACS. The long-lasting nature of this impact is likely due to other consequences of depression, as depressed patients are less likely to participate in cardiac rehabilitation programs. Anxiety was also a robust determinant of poor HRQOL following ACS, exhibiting a similar pattern of influence to that of depression [4,20,22,23,25,33,61,62]. Additionally, when anxiety was combined with depression, conceptualized as “emotional distress”, the impact was even worse, leading to worse outcomes across all dimensions of health status [23,61]. Furthermore, the presence of fatigue was strongly associated with broader deficits in well-being, correlating with worse scores in both physical and mental domains of HRQOL [31,52,53]. However, fatigue may not act as an isolated determinant but rather as a complex, overlapping symptom reflecting both underlying psychological distress and physical deconditioning.

### 4.5. Personality Traits and Coping Mechanisms

While core personality traits are generally stable and non-modifiable, the coping mechanisms that patients employ can be modified through tailored interventions. Coping strategies and mechanisms that patients employ to cope with their condition play a crucial role in determining their post-ACS HRQOL [20,27,51]. Sense of coherence (SOC) acted as a protective factor for patients, as those with high levels consistently demonstrated improved outcomes compared with those with low SOC [42,53,66]. High SOC may help patients to perceive the cardiac event as manageable, reducing the psychological shock and trauma. Other coping mechanisms such as problem-focused [51] and task-oriented coping [20] were identified as adaptive strategies associated with better outcomes, as they encourage active engagement with secondary prevention. Minimization also appeared to have a protective effect on mental health, showing a positive association with Mental Component Summary scores [27], likely by acting as a short-term coping mechanism against overwhelming anxiety. In contrast, fatalism was found to have a negative association with physical health in both sexes [27], while avoidance coping predicted worse emotional outcomes in men [20]. These mechanisms usually lead to symptom denial, delayal of the seeking of necessary medical care, and prevention of constructive health behavior modifications.

### 4.6. Clinical Factors and Factors Related to Acute Coronary Syndromes

Different clinical and ACS-related factors were the most widely investigated in the literature, including 27 determinants of HRQOL, comprising both non-modifiable and modifiable factors. Regarding the non-modifiable factors, the specific type of ACS was found to influence quality of life. When comparing the spectrum of ACS diagnoses, unstable angina was identified as a predictor of poorer physical recovery compared to acute myocardial infarction (AMI) [26,49]. Comparing patients with “ST” and “non-ST” elevation myocardial infarction, the available evidence in this review indicates an advantage for patients presenting with STEMI who usually report better health outcomes [59,60]. This somewhat paradoxical finding may be explained by the fact that STEMI patients undergo immediate revascularization protocols that often completely resolve ischemia. The severity of infarction, as measured by established prognostic indexes and classifications, was also found to be a significant determinant of post-ACS outcomes [22,24,25]. Specifically, higher scores on the Peel Index, indicating greater severity and worse prognosis, were repeatedly associated with poorer overall HRQOL [22,25]. Similarly, Killip classification, used to assess heart failure severity in acute MI, shows that patients with no signs of heart failure (Killip Class I) were more likely to demonstrate improved physical functioning [24,25]. Objective measures of cardiac function, such as Left Ventricular Ejection Fraction (LVEF), were identified as key clinical determinants of HRQOL. The literature demonstrates a negative correlation where lower baseline LVEF is associated with worse overall quality of life [35,41,58,60]. Moderate or severe left ventricular systolic dysfunction observed during the index hospitalization was associated with significant decline in physical function over time. In addition, having a history of a previous percutaneous coronary intervention (PCI) [28] or a Coronary Artery Bypass Graft (GABG) surgery [24,26,56] was associated with worse HRQOL.

Conversely, several clinical factors can be actively modified through medical management. The burden of continued or recurrent cardiac symptoms including chest pain, dyspnea, weakness, and sleep disturbances were found to be negatively associated with both physical and mental health [18,27,44,49]. Type and time of receiving therapy were also significant factors influencing HRQOL. Revascularization during the index hospitalization (either with PCI or with CABG) was significantly associated with improved physical and mental health status [26]. However, undergoing revascularization during the post-discharge interim period, although in some cases improved HRQOL [26,36,49], in other cases was found to worsen patient perspectives of their quality of life [30,49,51], as it can be viewed as a marker of clinical instability. Finally, time passed from the ACS event was also an important factor predicting HRQOL. The passage of time served as a determinant of recovery and generally acted as a positive factor in the evolution of patients’ physical and mental health [49,57].

### 4.7. Risk Factors for ACS and Patient Comorbidities

The burden of comorbid conditions was consistently identified as a major negative determinant of HRQOL in ACS patients [18,28,33,49,55,61]. While a patient’s history of comorbid conditions is technically non-modifiable at the time of the ACS event, the optimal clinical control of these conditions remains a highly modifiable priority. The literature reveals a clear inverse relationship between the number of comorbidities and health outcomes. The cumulative burden of comorbid conditions drives worse outcomes through competing physical limitations and complex polypharmacy. Patients with a greater number of comorbid illnesses reported significantly worse self-reported general health status, lower physical health and in some cases lower scores in social relations domains. Specifically, diabetes mellitus was identified as a substantial risk factor for poorer HRQOL following ACS [26,35,63]. While most findings indicate an adverse association, some studies suggested a positive association between Type 2 diabetes and global HRQOL [15,24]. History of stroke [26,28,56,63] and other clinical conditions, such as health failure [26,28,41], hypertension [55,57], coronary heart disease [49,57], chronic lung disease [35], hypercholesterolemia [56], anemia [41], etc., were found to be adversely associated with overall HRQOL, acting as significant predictors of poorer outcomes.

### 4.8. Previous Quality of Life and Dimensions

The patients’ quality-of-life status prior to or at the time of the acute event emerged as a non-modifiable, yet fundamental, prognostic component of their long-term recovery. The data indicated that patients with higher baseline QOL scores consistently reported better outcomes at follow-up assessments across physical, mental and global HRQOL [24,33,34,40,59]. Similarly, patients with better initial mental health consistently reported better perceived health status both at baseline and post-discharge [46,49,52,57]. Additionally, better physical function was associated with better health status and better mental health outcomes, suggesting that physical health provides also a foundation for psychological stability [2,41,46]. Preserved mobility and physical independence directly facilitate continued social participation, thereby preventing post-ACS isolation and feelings of depression.

### 4.9. Health System Factor

Beyond patient and clinical factors, structural factors related to the healthcare system also play a role in recovery outcomes and serve as potentially modifiable areas for quality improvement. Access to care was identified as a significant determinant of physical recovery. Data indicated that poorer access to healthcare services was associated with measurable decline in physical function as measured by the SF-12 Physical Component Summary [41]. This suggests that systemic barriers to receiving care can directly impede the physical rehabilitation of ACS patients, preventing them from maintaining or improving their functional status after the acute event.

### 4.10. Limitations

Several limitations should be considered when interpreting the findings of this systematic review. First, the search strategy was restricted to English language publications across four major databases, which may have resulted in the exclusion of studies available in other languages or other databases. Moreover, gray literature was not included in the review, raising the possibility that important data may have been missing. Furthermore, the potential influence of publication bias cannot be ruled out, as studies with statistically significant or positive results are more likely to be published than those with negative findings. Additionally, we used restrictive inclusion and exclusion criteria. We excluded studies focusing exclusively on specific subgroups (e.g., sex-specific, age-specific, or acute coronary syndrome subtype-specific populations), intervention-based designs (including cardiac rehabilitation), and populations defined by specific comorbidities. While this approach was chosen to reduce clinical heterogeneity and to focus on determinants of HRQOL in broadly defined post-ACS populations, it may have led to the exclusion of clinically relevant evidence. As a result, potentially important subgroup-specific or intervention-related determinants of HRQOL may not be captured in this review. Consequently, the generalizability of the findings may be limited, particularly for patient groups that are underrepresented or excluded from the included studies. Determinants of HRQOL may differ by sex, age, ACS subtype, comorbidity burden, or exposure to structured interventions such as cardiac rehabilitation, and these effects could not be examined within the scope of the present review. The chosen criteria therefore reflect a methodological compromise aimed at improving comparability across studies, while acknowledging the resulting limitations in scope. Future systematic reviews and meta-analyses should specifically address HRQOL determinants within defined subgroups (e.g., sex, age, ACS subtype, or comorbidity profiles) and evaluate the modifying role of interventions such as cardiac rehabilitation to provide more granular and clinically applicable insights. In addition, concerns regarding representativeness should be acknowledged. A substantial proportion of the included studies relied on convenience sampling, which introduces the possibility of selection bias, as patients recruited through non-probability methods may differ systematically from the broader post-ACS population. Moreover, most study populations were predominantly male. Although this reflects persistent patterns in cardiovascular research participation, it limits the external validity of our findings. These issues are particularly relevant given that female sex was identified as a determinant of HRQOL in this review. The underrepresentation of females may have led to imprecise or attenuated estimates of sex-related effects and may limit the ability to fully characterize the nature and magnitude of HRQOL determinants among female patients following ACS. Taken together, the restrictive eligibility criteria, reliance on convenience sampling, and sex imbalance suggest that the findings should be interpreted with caution, particularly when extrapolating to underrepresented patient groups or different clinical contexts. Future research should prioritize population-based sampling strategies, ensure adequate representation of females, and explore HRQOL determinants within specific subgroups and in relation to structured interventions, such as cardiac rehabilitation, to provide more comprehensive and clinically applicable insights. Finally, a further limitation relates to the substantial heterogeneity of the included studies, which precluded meta-analysis. As we mentioned in detail above, the studies varied considerably with respect to HRQOL measurement instruments, study designs, patient populations, and follow-up durations. While these sources of variability were considered collectively when determining that meta-analysis was not appropriate, they were not examined in a structured manner sufficient to support subgroup quantitative analyses. The absence of subgroup analyses or quantitative synthesis limits the ability to estimate the relative magnitude or consistency of associations between specific determinants and HRQOL outcomes and may reduce the strength of our findings. As a result, the conclusions of this review primarily reflect patterns of association rather than pooled effect estimates and should be interpreted as descriptive rather than quantitatively definitive. Although a narrative synthesis was deemed the most methodologically appropriate approach given the available data, future reviews may benefit from restricting inclusion to more homogeneous study subsets or focusing on specific HRQOL instruments or follow-up periods to enable meta-analysis or subgroup analyses.

## 5. Conclusions

In conclusion, this systematic review underscores the multiple factors that influence HRQOL following an ACS, identifying at least 87 distinct determinants. The findings reveal that depression and female sex were the most common predictors of poor outcomes, while psychological factors such as depression and anxiety can have a lasting impact on patients’ recovery. Furthermore, the study highlights the importance of social support, healthy patient behaviors and better baseline QOL as protective factors for better post-ACS health status. These results indicate the need for integrated, patient-centered post-ACS care. Clinical practice should therefore focus on targeted strategies that optimize modifiable factors, prioritizing early psychological screening and individualized support for high-risk groups, to effectively improve HRQOL and overall quality of care.

## Figures and Tables

**Table 1:** Analysis of the studies included.

	Reference	Continent	Country	Data Collection Time	Sample Size	Age, Mean (Standard Deviation)	Sex, Males (%)	Study Design	Sampling Method	Response Rate (%)
1	Bogg et al., 2000 [20]	Europe	United Kingdom	A 12-month period (NR)	220	♂ 60 (9.9) years ♀ 61 (8.8) years	77	Cohort	Convenience sampling	95
2	Fritz, 2000 [21]	North America	USA	NR	65	♀ 62.0 years ♂ 54.8 years	69.2	Cohort	Convenience sampling	81
3	Lane et al., 2000 [22]	Europe	United Kingdom	January 1997 to August 1998	288	62.7 (11.5) years	74.7	Cohort	Convenience sampling	65.9
4	Mayou et al., 2000 [23]	Europe	United Kingdom	November 14, 1994, to November 13, 1995	344	63.16 years	73	Cohort	Convenience sampling	71
5	Radley et al., 2000 [18]	Europe	United Kingdom	NR	120	NR	50	Cross-sectional	Convenience sampling	69
6	Beck et al., 2001 [24]	North America	Canada	December 28, 1996, to November 1, 1998.	587	Median age, 61 years	NR	Cohort	Convenience sampling	82
7	Lane et al., 2001 [25]	Europe	United Kingdom	January 1997 to August 1998	288	62.7 (11.5) years	74.7	Cohort	Convenience sampling	65.9
8	Rumsfeld et al., 2001 [26]	North America	USA	March 1998 to February 1999	1660	65.5 (10.7) years	98.0	Cohort	Convenience sampling	68.4
9	Brink et al., 2002 [27]	Europe	Sweden	October 1998 to September 1999	114	♀ 72.2 (8.6) years ♂ 65.4 (10.1) years	67.5	Cross-sectional	Convenience sampling	83
10	McBurney et al., 2002 [28]	North America	USA	July 1999 to July 2000	200	63.4 (13.1) years	68	Cross-sectional	Convenience sampling	78
11	Rumsfeld et al., 2003 [29]	North America	USA	March 1998 to February 1999	1957	65.2 years	Males without depression history: 97.8 Males with depression history: 97.9	Cohort	Convenience sampling	80.6
12	Bengtsson et al., 2004 [30]	Europe	Sweden	NR	72	59 years	81	Cohort	Convenience sampling	87.5
13	Brink et al., 2005 [31]	Europe	Sweden	October 1998 to September 1999	98	67.9 (9.95) years	66.3	Cohort	Convenience sampling	74
14	Fauerbach et al., 2005 [32]	North America	USA	An 18-month period (NR)	196	NR	57.1	Cohort	Convenience sampling	68.8
15	Spertus et al., 2005 [19]	North America	USA	1 February 2000 to 31 October 2001	1159	Black patients: 55 (11) years White patients: 63 (13) years	Black patients: males 49 White patients: males 64	Cohort	Convenience sampling	NR
16	Dickens et al., 2006 [33]	Europe	United Kingdom	NR	260	57.6 (11.2) years	63	Cohort	Convenience sampling	88
17	De Jonge et al., 2006 [34]	Europe	Netherlands	September 1997 to September 2000	421	61.0 (11.4) years	79.6	Cohort	Convenience sampling	90.0
18	Peterson et al., 2006 [35]	North America	USA	1 March 2001 to 31 October 2002	1199	Patients with diabetes: 62 (12) years, patients without diabetes: 61 (13) years	Males with diabetes: 51, males without diabetes: 65	Cohort	Convenience sampling	75
19	Failde & Soto, 2006 [36]	Europe	Spain	NR	132	NR	73.2	Cohort	Convenience sampling	57.6
20	Norris et al., 2007 [37]	North America	Canada	NR	486	♂ 59 years ♀ 66 years	79	Cohort	Convenience sampling	82
21	Rahimi et al., 2007 [38]	North America	USA	1 January 2003, and 28 June 2004	2498	60.9 (13) years	67.4	Cohort	Random	NR
22	Ho et al., 2008 [39]	North America	USA	1 January 2003, to 28 June 2004	2498	60.9 (13) years	67	Cohort	Random	NR
23	Thombs et al., 2008 [40]	North America	Canada	NR	425	62.4 (10.8) years	71.5	Cohort	Convenience sampling	50
24	Arnold et al., 2009 [41]	North America	United States of America	January 2003 to June 2004	2481	NR	NR	Cohort	Random	63,2
25	Bergman et al., 2009 [42]	Europe	Sweden	November 2003 to June 2005	100	Median age: 57 years	79	Cohort	Convenience sampling	NR
26	Spertus et al., 2009 [43]	North America	USA	1 January 2003 to 28 June 2004	1849	White patients: 61.7 (12.9) years, black patients: 57.3 (13.2) years	White males: 70.6, black males 55.1	Cohort	Random	NR
27	Arnold et al., 2009 [44]	North America	USA	January 2003 to June 2004	1835	NR	NR	Cohort	Random	NR
28	Leifheit-Limson et al., 2010 [45]	North America	USA	January 2003 to June 2004	2411	60.8 years	67	Cohort	Random	NR
29	Shin & Choi, 2010 [46]	North America	USA	December 2003 to September 2004	82	63 (11.2) years	53.7	Cohort	Convenience sampling	82.
30	de Jong-Watt & Sherifi, 2011 [47]	North America	Canada	A four-month data collection period (NR)	36	62.6 (14.38) years	58.3	Cross-sectional	Convenience sampling	97.3
31	Bucholz et al., 2011 [48]	North America	USA	1 January 2003, to 28 June 2004	2264	Patients living alone: 62.7 (13.5) years, patients not living alone: 59.3 (12.3) years	Males living alone: 57.5, males not living alone: 70.8	Cohort	Random	NR
32	Dueñas et al., 2011 [49]	Europe	Spain	NR	175	♂ 67.11 (10.9) years ♀ 69.76 (9.9) years	64	Cohort	Convenience sampling	100
33	Ginzburg & Ein-Dor, 2011 [50]	Asia	Israel	8-year period (NR)	196 baseline	Recovered/resilient group: 54.02 (8.21), chronic group: 54.73 (9.57)	84	Cohort	Convenience sampling	80
34	Panthee et al., 2011 [51]	Asia	Nepal	October 2010 to January 2011	88	57.43 (11.41) years	72.7	Cross-sectional	Convenience sampling	NR
35	Brink et al., 2012 [52]	Europe	Sweden	NR	145	64.4 (9.4) years	70.3	Cohort	Convenience sampling	86.3
36	Leifheit-Limson et al., 2012 [14]	North America	USA	January 2003 to June 2004	1951	NR	NR	Cohort	Convenience sampling	NR
37	Brink, 2012 [53]	Europe	Sweden	October 1998 September 1999	98	67.9 (9.95) years	66.3	Cross-sectional	Convenience sampling	74
38	Williams et al., 2012 [54]	Europe	United Kingdom	NR	192	66.0 (10.8) years	71.9	Cohort	Convenience sampling	97.5
39	Sertoz et al., 2013 [55]	Asia	Turkey	January 2006 to October 2008	998	57.5 (10.1) years	79.2	Cross-sectional	Convenience sampling	NR
40	Hosseini et al., 2014 [56]	Asia	Iran	NR	196	55.8 (11.1) years	74	Cohort	Convenience sampling	60%
41	Bennett et al., 2015 [2]	North America	USA	1 January 2003 to June 28, 2004	2348	60.6 (13.0) years	67.6	Cohort	Random	63.2
42	Salazar et al., 2016 [57]	Europe	Spain	NR	250	65.5 (11.3) years	69.2	Cohort	Convenience sampling	62
43	Dzubur et al., 2016 [58]	Europe	Bosnia and Herzegovina	NR	160	54.9 (8.8) years	81.3	Cohort	Random	NR
44	Mahesh et al., 2017 [59]	Asia	Sri Lanka	1st of January 2015 to March 2015	344	median (IQR) = 62.0 (54.0–70.0).	69.4	Cross-sectional	Convenience sampling	68.3
45	Kang et al., 2018 [60]	Asia	South Korea	August 2015 to February 2016	150	64.63 (11.48) years	71.3	Cohort	Convenience sampling	69.8
46	Xia et al., 2019 [61]	Asia	North China	January 2013 to June 2015	647	63.4 (12.0) years	67.7	Cohort	Convenience sampling	82.5
47	Wulandari et al., 2020 [62]	Asia	Indonesia	November 2016 to June 2017	214	58 (18) years	88	Cross-sectional	Convenience sampling	NR
48	Kang et al., 2021 [63]	Asia	South Korea	August 2015 to February 2016	150	64.63 (11.48) years	71.3	Cross-sectional	Convenience sampling	69.8
49	Džubur et al., 2022 [64]	Europe	Bosnia and Herzegovina	January 2020 to July 2020	120	64.73 (11.218) years	58.3	Cross-sectional	Convenience sampling	NR
50	Rasmussen et al., 2022 [3]	Europe	Denmark	15 April 2013 to 15 April 2014	2131	64.3 (11.8) years	75.4	Cross-sectional	Convenience sampling	45.5
51	Upadhyay et al., 2022 [65]	Asia	India	January 2018 to February 2019	103	59.66 (10.42) years	76.7	Cross-sectional	Convenience sampling	94.5
52	Jlassi et al., 2024 [15]	Africa	Tunisia.	June 2018 to December 2019	50	51.92 (6.4) years	90	Cohort	Convenience sampling	NR
53	Sauletzhanovna et al., 2024 [4]	Asia	Iraq	Patients recruited over 12 months and followed up for 5 years (period NR)	1000	58.4 (12.3) years	68	Cohort	Convenience sampling	NR
54	Füller et al., 2025 [5]	Europe	Germany	April 2019 to December 2020	298	66.6 (13.0) years	71.8	Cohort	Convenience sampling	70.1
55	Malm et al., 2025 [66]	Europe	Sweden	November 2003 to June 2005	61	57.1 (6.5) years	73.8	Cohort	Convenience sampling	61

♂: males; ♀: females. NR: not reported.

## Data Availability

No new data were created or analyzed in this study.

## Supplementary Materials

The following supporting information can be downloaded at https://www.mdpi.com/article/10.3390/healthcare14101292/s1. Table S1: Quality assessment of the studies included in this systematic review; Table S2: Determinants of health-related quality of life after acute coronary syndromes and their frequency across studies; Table S3: Associations between determinants and health-related quality of life after acute coronary syndromes; Table S4: Determinants of health-related quality of life after acute coronary syndromes.

## Abbreviations

The following abbreviations are used in this manuscript:

ACE	Angiotensin-Converting Enzyme Inhibitors
ACS	Acute Coronary Syndrome
AMI	Acute Myocardial Infarction
CABG	Coronary Artery Bypass Graft
EQ-5D	Euro-QoL 5 Dimensions
HRQOL	Health-Related Quality of Life
LVEF	Left Ventricular Ejection Fraction
MI	Myocardial Infarction
NR	Not Reported
NSTEMI	“Non-ST” Elevation Myocardial Infarction
PCI	Percutaneous Coronary Intervention
PRISMA	Preferred Reporting Items for Systematic Reviews and Meta-Analyses
PROMs	Patient-Reported Outcomes Measures
QOL	Quality of Life
SAQ	Seattle Angina Questionnaire
SOC	Sense of Coherence
STEMI	“ST” Elevation Myocardial Infarction
UK	United Kingdom
USA	United States of America

## References

[1] Rao S.V., O’Donoghue M.L., Ruel M., Rab T., Tamis-Holland J.E., Alexander J.H., Baber U., Baker H., Cohen M.G., Cruz-Ruiz M. (2025). 2025 ACC/AHA/ACEP/NAEMSP/SCAI Guideline for the Management of Patients With Acute Coronary Syndromes. JACC.

[2] Bennett K.K., Buchanan D.M., Jones P.G., Spertus J.A. (2015). Socioeconomic Status, Cognitive-Emotional Factors, and Health Status Following Myocardial Infarction: Testing the Reserve Capacity Model. J. Behav. Med..

[3] Rasmussen A.A., Fridlund B., Nielsen K., Rasmussen T.B., Thrysoee L., Borregaard B., Thorup C.B., Berg S.K., Mols R.E. (2022). Gender Differences in Patient-Reported Outcomes in Patients with Acute Myocardial Infarction. Eur. J. Cardiovasc. Nurs..

[4] Sauletzhanovna T.A., Mohammed W.K., Ahmed A.S., Mohammed H.I., Al-Hili A., Alnajar M.J., Naser N.S., Amr E.F., Mohsin R.M. (2024). The Predictive Value of Depression and Anxiety on Protracted Cardiovascular Outcomes in Individuals with Acute Myocardial Infarction. Int. J. Body Mind Cult..

[5] Füller D., Andresen-Bundus H., Pagonas N., Jaehn P., Ukena C., Gödde K., Holmberg C., Ritter O., Sasko B. (2025). Adverse Socioeconomic Factors Are Associated with a Widening Gap in One-Year Health-Related Quality of Life after Acute Myocardial Infarction. Sci. Rep..

[6] Candelaria D., Randall S., Ladak L., Gallagher R. (2020). Health-Related Quality of Life and Exercise-Based Cardiac Rehabilitation in Contemporary Acute Coronary Syndrome Patients: A Systematic Review and Meta-Analysis. Qual. Life Res..

[7] Takousi M.G., Schmeer S., Manaras I., Olympios C.D., Makos G., Troop N.A. (2016). Health-Related Quality of Life after Coronary Revascularization: A Systematic Review with Meta-Analysis. Hell. J. Cardiol..

[8] Pons A., Whalley G., Sneddon K., Williams M., Coffey S. (2022). Predictors of Quality of Life after Revascularization for Ischemic Heart Disease: A Systematic Review. Health Sci. Rev..

[9] Foxwell R., Morley C., Frizelle D. (2013). Illness Perceptions, Mood and Quality of Life: A Systematic Review of Coronary Heart Disease Patients. J. Psychosom. Res..

[10] Dickens C., Cherrington A., McGowan L. (2012). Depression and Health-Related Quality of Life in People with Coronary Heart Disease: A Systematic Review. Eur. J. Cardiovasc. Nurs..

[11] Zhu C., Tran P.M., Leifheit E.C., Spatz E.S., Dreyer R.P., Nyhan K., Wang S.-Y., Lichtman J.H. (2023). Association of Marital/Partner Status and Patient-Reported Outcomes Following Myocardial Infarction: A Systematic Review and Meta-Analysis. Eur. Heart J. Open.

[12] Simpson E., Pilote L. (2003). Quality of Life after Acute Myocardial Infarction: A Systematic Review. Can. J. Cardiol..

[13] Kang K., Gholizadeh L., Inglis S.C., Han H.-R. (2017). Correlates of Health-Related Quality of Life in Patients with Myocardial Infarction: A Literature Review. Int. J. Nurs. Stud..

[14] Leifheit-Limson E.C., Reid K.J., Kasl S.V., Lin H., Buchanan D.M., Jones P.G., Peterson P.N., Parashar S., Spertus J.A., Lichtman J.H. (2012). Changes in Social Support within the Early Recovery Period and Outcomes after Acute Myocardial Infarction. J. Psychosom. Res..

[15] Jlassi O., Omrane A., Ben Massoud M., Khalfallah T., Bouzgarrou L., Gamra H. (2024). Determinants of Health-Related Quality of Life among Patients with Ischemic Heart Disease. Health Syst..

[16] Page M.J., McKenzie J.E., Bossuyt P.M., Boutron I., Hoffmann T.C., Mulrow C.D., Shamseer L., Tetzlaff J.M., Akl E.A., Brennan S.E. (2021). The PRISMA 2020 Statement: An Updated Guideline for Reporting Systematic Reviews. BMJ.

[17] Santos W.M.D., Secoli S.R., Püschel V.A.D.A. (2018). The Joanna Briggs Institute Approach for Systematic Reviews. Rev. Lat. Am. Enferm..

[18] Radley A., Grove A., Wright S., Thurston H. (2000). Gender-Role Identity after Heart Attack: Links with Sex and Subjective Health Status. Psychol. Health.

[19] Spertus J., Safley D., Garg M., Jones P., Peterson E.D. (2005). The Influence of Race on Health Status Outcomes One Year After an Acute Coronary Syndrome. J. Am. Coll. Cardiol..

[20] Bogg J., Thornton E., Bundred P. (2000). Gender Variability in Mood, Quality of Life and Coping Following Primary Myocardial Infarction. Coron. Health Care.

[21] Fritz H.L. (2000). Gender-Linked Personality Traits Predict Mental Health and Functional Status Following a First Coronary Event. Health Psychol..

[22] Lane D., Carroll D., Ring C., Beevers D.G., Lip G.Y.H. (2000). Effects of Depression and Anxiety on Mortality and Quality-of-Life 4 Months after Myocardial Infarction. J. Psychosom. Res..

[23] Mayou R.A., Gill D., Thompson D.R., Day A., Hicks N., Volmink J., Neil A. (2000). Depression and Anxiety As Predictors of Outcome After Myocardial Infarction. Psychosom. Med..

[24] Beck C.A., Joseph L., Bélisle P., Pilote L. (2001). Predictors of Quality of Life 6 Months and 1 Year after Acute Myocardial Infarction. Am. Heart J..

[25] Lane D., Carroll D., Ring C., Beevers D.G., Lip G.Y.H. (2001). Mortality and Quality of Life 12 Months After Myocardial Infarction: Effects of Depression and Anxiety. Psychosom. Med..

[26] Rumsfeld J.S., Magid D.J., Plomondon M.E., O’Brien M.M., Spertus J.A., Every N.R., Sales A.E. (2001). Predictors of Quality of Life Following Acute Coronary Syndromes. Am. J. Cardiol..

[27] Brink E., Karlson B.W., Hallberg L.R.-M. (2002). Health Experiences of First-Time Myocardial Infarction: Factors Influencing Women’s and Men’s Health-Related Quality of Life after Five Months. Psychol. Health Med..

[28] McBurney C.R., Eagle K.A., Kline-Rogers E.M., Cooper J.V., Mani O.C.M., Smith D.E., Erickson S.R. (2002). Health-Related Quality of Life in Patients 7 Months After a Myocardial Infarction: Factors Affecting the Short Form-12. Pharmacother. J. Hum. Pharmacol. Drug Ther..

[29] Rumsfeld J.S., Magid D.J., Plomondon M.E., Sales A.E., Grunwald G.K., Every N.R., Spertus J.A. (2003). History of Depression, Angina, and Quality of Life after Acute Coronary Syndromes. Am. Heart J..

[30] Bengtsson I., Hagman M., Währborg P., Wedel H. (2004). Lasting Impact on Health-Related Quality of Life after a First Myocardial Infarction. Int. J. Cardiol..

[31] Brink E., Grankvist G., Karlson B.W., Hallberg L.R.-M. (2005). Health-Related Quality of Life in Women and Men One Year after Acute Myocardial Infarction. Qual. Life Res..

[32] Fauerbach J.A., Bush D.E., Thombs B.D., McCann U.D., Fogel J., Ziegelstein R.C. (2005). Depression Following Acute Myocardial Infarction: A Prospective Relationship With Ongoing Health and Function. Psychosomatics.

[33] Dickens C.M., McGowan L., Percival C., Tomenson B., Cotter L., Heagerty A., Creed F.H. (2006). Contribution of Depression and Anxiety to Impaired Health-Related Quality of Life Following First Myocardial Infarction. Br. J. Psychiatry.

[34] De Jonge P., Spijkerman T.A., Van Den Brink R.H.S., Ormel J. (2006). Depression after Myocardial Infarction Is a Risk Factor for Declining Health Related Quality of Life and Increased Disability and Cardiac Complaints at 12 Months. Heart.

[35] Peterson P.N., Spertus J.A., Magid D.J., Masoudi F.A., Reid K., Hamman R.F., Rumsfeld J.S. (2006). The Impact of Diabetes on One-Year Health Status Outcomes Following Acute Coronary Syndromes. BMC Cardiovasc. Disord..

[36] Failde I.I., Soto M.M. (2006). Changes in Health Related Quality of Life 3 Months after an Acute Coronary Syndrome. BMC Public Health.

[37] Norris C.M., Hegadoren K., Pilote L. (2007). Depression Symptoms Have a Greater Impact on the 1-Year Health-Related Quality of Life Outcomes of Women Post-Myocardial Infarction Compared to Men. Eur. J. Cardiovasc. Nurs..

[38] Rahimi A.R., Spertus J.A., Reid K.J., Bernheim S.M., Krumholz H.M. (2007). Financial Barriers to Health Care and Outcomes After Acute Myocardial Infarction. JAMA.

[39] Ho P.M., Eng M.H., Rumsfeld J.S., Spertus J.A., Peterson P.N., Jones P.G., Peterson E.D., Alexander K.P., Havranek E.P., Krumholz H.M. (2008). The Influence of Age on Health Status Outcomes after Acute Myocardial Infarction. Am. Heart J..

[40] Thombs B.D., Ziegelstein R.C., Stewart D.E., Abbey S.E., Parakh K., Grace S.L. (2008). Usefulness of Persistent Symptoms of Depression to Predict Physical Health Status 12 Months After an Acute Coronary Syndrome. Am. J. Cardiol..

[41] Arnold S.V., Alexander K.P., Masoudi F.A., Ho P.M., Xiao L., Spertus J.A. (2009). The Effect of Age on Functional and Mortality Outcomes After Acute Myocardial Infarction. J. Am. Geriatr. Soc..

[42] Bergman E., Malm D., Karlsson J.-E., Berterö C. (2009). Longitudinal Study of Patients after Myocardial Infarction: Sense of Coherence, Quality of Life, and Symptoms. Heart Lung.

[43] Spertus J.A., Jones P.G., Masoudi F.A., Rumsfeld J.S., Krumholz H.M. (2009). Factors Associated With Racial Differences in Myocardial Infarction Outcomes. Ann. Intern. Med..

[44] Arnold S.V., Spertus J.A., Jones P.G., Xiao L., Cohen D.J. (2009). The Impact of Dyspnea on Health-Related Quality of Life in Patients with Coronary Artery Disease: Results from the PREMIER Registry. Am. Heart J..

[45] Leifheit-Limson E.C., Reid K.J., Kasl S.V., Lin H., Jones P.G., Buchanan D.M., Parashar S., Peterson P.N., Spertus J.A., Lichtman J.H. (2010). The Role of Social Support in Health Status and Depressive Symptoms After Acute Myocardial Infarction: Evidence for a Stronger Relationship Among Women. Circ. Cardiovasc. Qual. Outcomes.

[46] Shin N.-M., Choi J. (2010). Relationship Between Survivors’ Perceived Health Status Following Acute Coronary Syndrome and Depression Symptoms During Early Recovery Phase. Asian Nurs. Res..

[47] de Jong-Watt W., Sherifi I. (2011). Patient-Centred Assessment of Social Support, Health Status and Quality of Life in Patients with Acute Coronary Syndrome. Can. J. Cardiovasc. Nurs..

[48] Bucholz E.M., Rathore S.S., Gosch K., Schoenfeld A., Jones P.G., Buchanan D.M., Spertus J.A., Krumholz H.M. (2011). Effect of Living Alone on Patient Outcomes After Hospitalization for Acute Myocardial Infarction. Am. J. Cardiol..

[49] Dueñas M., Ramirez C., Arana R., Failde I. (2011). Gender Differences and Determinants of Health Related Quality of Life in Coronary Patients: A Follow-up Study. BMC Cardiovasc. Disord..

[50] Ginzburg K., Ein-Dor T. (2011). Posttraumatic Stress Syndromes and Health-Related Quality of Life Following Myocardial Infarction: 8-Year Follow-Up. Gen. Hosp. Psychiatry.

[51] Panthee B., Kritpracha C., Chinnawong T. (2011). Correlation between Coping Strategies and Quality of Life among Myocardial Infarction Patients in Nepal. Nurse Media J. Nurs..

[52] Brink E., Alsén P., Herlitz J., Kjellgren K., Cliffordson C. (2012). General Self-Efficacy and Health-Related Quality of Life after Myocardial Infarction. Psychol. Health Med..

[53] Brink E. (2012). Considering Both Health-Promoting and Illness-Related Factors in Assessment of Health-Related Quality of Life After Myocardial Infarction. Open Nurs. J..

[54] Williams L., O’Connor R.C., Grubb N.R., O’Carroll R.E. (2012). Type D Personality and Three-Month Psychosocial Outcomes among Patients Post-Myocardial Infarction. J. Psychosom. Res..

[55] Sertoz O.O., Aydemir O., Gulpek D., Elbi H., Ozenli Y., Yilmaz A., Ozan E., Atesci F., Abay E., Semiz M. (2013). The Impact of Physical and Psychological Comorbid Conditions on the Quality of Life of Patients with Acute Myocardial Infarction: A Multi-Center, Cross-Sectional Observational Study from Turkey. Int. J. Psychiatry Med..

[56] Hosseini S.H., Ghaemian A., Mehdizadeh E., Ashraf H. (2014). Contribution of Depression and Anxiety to Impaired Quality of Life in Survivors of Myocardial Infarction. Int. J. Psychiatry Clin. Pract..

[57] Salazar A., Dueñas M., Fernandez-Palacin F., Failde I. (2016). Factors Related to the Evolution of Health Related Quality of Life in Coronary Patients. A Longitudinal Approach Using Weighted Generalized Estimating Equations with Missing Data. Int. J. Cardiol..

[58] Dzubur A., Mekic M., Pesto S., Nabil N. (2016). Echocardiographic Parameters as Life Quality Predictors in Patients After Myocardial Infarction Treated with Different Methods. Med. Arch..

[59] Mahesh P.K.B., Gunathunga M.W., Jayasinghe S., Arnold S.M., Haniffa R., De Silva A.P. (2017). Pre-Event Quality of Life and Its Influence on the Post-Event Quality of Life among Patients with ST Elevation and Non-ST Elevation Myocardial Infarctions of a Premier Province of Sri Lanka. Health Qual. Life Outcomes.

[60] Kang K., Gholizadeh L., Han H.-R., Inglis S.C. (2018). Predictors of Health-Related Quality of Life in Korean Patients with Myocardial Infarction: A Longitudinal Observational Study. Heart Lung.

[61] Xia K., Wang L.-F., Yang X.-C., Jiang H.-Y., Zhang L.-J., Yao D.-K., Hu D.-Y., Ding R.-J. (2019). Comparing the Effects of Depression, Anxiety, and Comorbidity on Quality-of-Life, Adverse Outcomes, and Medical Expenditure in Chinese Patients with Acute Coronary Syndrome. Chin. Med. J. (Engl.).

[62] Wulandari D., Ginanjar A.S., Purwono U., Purba D. (2020). Marital Satisfaction, Anxiety, and Health-Related Quality of Life in Myocardial Infarction Patients. J. Glob. Pharma Technol..

[63] Kang K., Gholizadeh L., Han H.-R. (2021). Health-Related Quality of Life and Its Predictors in Korean Patients with Myocardial Infarction in the Acute Phase. Clin. Nurs. Res..

[64] Džubur A., Lisica D., Hodžić E., Begić E., Lepara O., Fajkić A., Gogić E., Ejubović M. (2022). Relationship between Depression and Quality of Life after Myocardial Infarction. Med. Glas..

[65] Upadhyay V., Bhandari S.S., Rai D.P., Dutta S., García-Grau P., Vaddiparti K. (2022). Improving Depression and Perceived Social Support Enhances Overall Quality of Life among Myocardial Infarction Survivors: Necessity for Integrating Mental Health Care into Cardiac Rehabilitation Programs. Egypt. J. Neurol. Psychiatry Neurosurg..

[66] Malm D., Mårtensson J., Årestedt K. (2025). Sense of Coherence and Quality of Life in the Recovery of Women and Men with Myocardial Infarction: A 10-Year Follow-up Study. Eur. J. Cardiovasc. Nurs..

