# Supplementary material for: Determinants of Health-Related Quality of Life After Acute Coronary Syndromes: A Systematic Review
Source: Healthcare (Basel). 2026 May 9;14(10):1292. doi: 10.3390/healthcare14101292 (PMC13205943; doi:10.3390/healthcare14101292)
Supplement: Supplementary file 1 [file healthcare-14-01292-s001.zip › Supplementary Table S1.pdf]

Supplementary Table S1a. Quality assessment of the cross-sectional studies included in this systematic review.

	Reference	Were the criteria for inclusion in the sample clearly defined?	Were the study subjects and the setting described in detail?	Was the exposure measured in a valid and reliable way?	Were objective, standard criteria used for measurement of the condition?	Were confounding factors identified?	Were strategies to deal with confounding factors stated?	Were the outcomes measured in a valid and reliable way?	Was appropriate statistical analysis used?	Risk of Bias
1	Radley et al., 2000 [1]	Yes	No	Yes	Yes	Yes	Yes	Yes	Yes	Low
2	Brink et al., 2002 [2]	Yes	Yes	Yes	Yes	Yes	Yes	Yes	Yes	Low
3	McBurney et al., 2002 [3]	Yes	Yes	Yes	Yes	Yes	Yes	Yes	Yes	Low
4	de Jong-Watt & Sherifi, 2011 [4]	Yes	Yes	Yes	Yes	Yes	No	Yes	No	Low
5	Panthee et al., 2011 [5]	Yes	Yes	Yes	Yes	Yes	No	Yes	No	Low
6	Brink, 2012 [6]	Unclear	Yes	Yes	Yes	Yes	Yes	Yes	Yes	Low
7	Sertoz et al., 2013 [7]	Yes	Yes	Yes	Yes	Yes	Yes	Yes	Yes	Low
8	Mahesh et al., 2017 [8]	Yes	Yes	Yes	Yes	Yes	Yes	Yes	Yes	Low
9	Wulandari et al., 2020 [9]	Yes	Yes	Yes	Yes	Yes	Yes	Yes	Yes	Low
10	Kang et al., 2021 [10]	Yes	Yes	Yes	Yes	Yes	Yes	Yes	Yes	Low
11	Džubur et al., 2022 [11]	Yes	Unclear	Yes	Yes	Yes	Yes	Yes	Yes	Low
12	Rasmussen et al., 2022 [12]	Yes	Yes	Yes	Yes	Yes	Yes	Yes	Yes	Low
13	Upadhyay et al., 2022 [13]	Yes	Yes	Yes	Yes	Yes	Yes	Yes	Yes	Low

Supplementary Table S1b. Quality assessment of the cohort studies included in this systematic review.

	Reference	Were the two groups similar and recruited from the same population ?	Were the exposures measured similarly to assign people to both exposed and unexposed groups?	Was the exposure measured in a valid and reliable way?	Were confoundin g factors identified?	Were strategies to deal with confoundin g factors stated?	Were the groups/ participants free of the outcome at the start of the study (or at the moment of exposure)?	Were the outcomes measured in a valid and reliable way?	Was the follow up time reported and sufficient to be long enough for outcomes to occur?	Was follow up complete, and if not, were the reasons to loss to follow up described and explored?	Were strategies to address incomplete follow up utilized?	Was appropriate statistical analysis used?	Risk of bias
1	Bogg et al., 2000 [14]	Yes	Yes	Yes	Yes	Yes	Yes	Yes	Yes	Unclear	Unclear	Yes	Low
2	Fritz, 2000 [15]	Yes	Yes	Yes	Yes	Yes	Yes	Yes	Yes	Yes	No	Yes	Low
3	Lane et al., 2000 [16]	Yes	Yes	Yes	Yes	Yes	Yes	Yes	Yes	Yes	NA	Yes	Low
4	Mayou et al., 2000 [17]	Yes	Yes	Yes	Yes	Yes	Yes	Yes	Yes	Yes	Yes	Yes	Low
5	Beck et al., 2001 [18]	Yes	Yes	Yes	Yes	Yes	Yes	Yes	Yes	No	Yes	Yes	Low
6	Lane et al., 2001 [19]	Yes	Yes	Yes	Yes	Yes	Yes	Yes	Yes	Yes	NA	Yes	Low
7	Rumsfeld et al., 2001 [20]	Yes	Yes	Yes	Yes	Yes	Yes	Yes	Yes	No	No	Yes	Low
8	Rumsfeld et al., 2003 [21]	Yes	Yes	Unclear	Yes	Yes	Yes	Yes	Yes	No	Yes	Yes	Low
9	Bengtson et al., 2004 [22]	Yes	Yes	Yes	Yes	Yes	Yes	Yes	Yes	Yes	Yes	Yes	Low
10	Brink et al., 2005 [23]	Yes	Yes	Yes	Yes	Yes	Yes	Yes	Yes	Yes	No	Yes	Low
11	Fauerbach et al., 2005 [24]	Yes	Yes	Yes	Yes	Yes	Yes	Yes	Yes	Yes	No	Yes	Low
12	Spertus et al., 2005 [25]	Yes	Yes	Yes	Yes	Yes	Yes	Yes	Yes	Yes	Yes	Yes	Low
13	Dickens et al.,	Yes	Yes	Yes	Yes	Yes	Yes	Yes	Yes	Yes	Yes	Yes	Low

	2006 [26]												
14	de Jonge et al., 2006 [27]	Yes	Yes	Yes	Yes	Yes	Yes	Yes	Yes	Yes	Yes	Yes	Low
15	Peterson et al., 2006 [28]	Yes	Yes	Yes	Yes	Yes	Yes	Yes	Yes	Yes	Yes	Yes	Low
16	Failde & soto, 2006 [29]	Yes	Yes	Yes	Yes	Yes	Yes	Yes	Yes	No	Yes	Yes	Low
17	Norris et al., 2007 [30]	Yes	Yes	Yes	Yes	Yes	Yes	Yes	Yes	Yes	Yes	Yes	Low
18	Rahimi et al., 2007 [31]	Yes	Yes	Unclear	Yes	Yes	Yes	Yes	Yes	No	Yes	Yes	Low
19	Ho et al., 2008 [32]	Yes	Yes	Yes	Yes	Yes	Yes	Yes	Yes	Yes	Yes	Yes	Low
20	Thombs et al., 2008 [33]	Yes	Yes	Yes	Yes	Yes	Yes	Yes	Yes	Yes	Yes	Yes	Low
21	Arnold et al., 2009 [34]	Yes	Yes	Yes	Yes	Yes	Yes	Yes	Yes	Yes	Yes	Yes	Low
22	Bergman et al., 2009 [35]	Yes	Yes	Yes	Yes	Yes	Yes	Yes	No	Unclear	Unclear	Yes	Low
23	Spertus et al., 2009 [36]	Yes	Yes	No	Yes	Yes	Yes	Yes	Yes	Yes	Yes	Yes	Low
24	Arnold et al., 2009 [37]	Yes	Yes	Yes	Yes	Yes	Yes	Yes	Yes	Yes	Yes	Yes	Low
25	Leifheit-Limson et al., 2010 [38]	Yes	Yes	Yes	Yes	Yes	Yes	Yes	Yes	Yes	Yes	Yes	Low
26	Shin & Choi, 2010 [39]	Yes	Yes	Yes	Yes	Yes	Yes	Yes	No	No	Yes	Yes	Low
27	Bucholz et al., 2011 [40]	Yes	Yes	Yes	Yes	Yes	Yes	Yes	Yes	Yes	Yes	Yes	Low
28	Dueñas et al., 2011 [41]	Yes	Yes	Yes	Yes	Yes	Yes	Yes	Yes	No	Yes	Yes	Low
29	Ginzburg & Ein-dor, 2011 [42]	Yes	Yes	Yes	Yes	Yes	Yes	Yes	Yes	Yes	Yes	Yes	Low

30	Brink et al., 2012 [43]	Yes	Yes	Yes	Yes	Yes	Yes	Yes	Yes	No	No	Yes	Low
31	Leifheit-Limson et al., 2012 [44]	Yes	Yes	Yes	Yes	Yes	Yes	Yes	Yes	Yes	Yes	Yes	Low
32	Williams et al., 2012 [45]	Yes	Yes	Yes	Yes	Yes	Yes	Yes	Yes	Yes	No	Yes	Low
33	Hosseini et al., 2014 [46]	Yes	Yes	Yes	Yes	Yes	Yes	Yes	Yes	Yes	Yes	Yes	Low
34	Bennett et al., 2015 [47]	Yes	Yes	Yes	Yes	Yes	Yes	Yes	Yes	Yes	Yes	Yes	Low
35	Salazar et al., 2016 [48]	Yes	Yes	Yes	Yes	Yes	Yes	Yes	Yes	Yes	Yes	Yes	Low
36	Dzubur et al., 2016 [49]	Yes	Yes	Yes	Yes	Yes	Yes	Yes	Yes	Yes	NA	Yes	Low
37	Kang et al., 2018 [50]	Yes	Yes	Yes	Yes	Yes	Yes	Yes	Yes	Yes	Yes	Yes	Low
38	Xia et al., 2019 [51]	Yes	Yes	Yes	Yes	Yes	Yes	Yes	Yes	No	No	Yes	Low
39	Jlassi et al., 2023 [52]	Yes	Yes	Yes	Yes	Yes	Yes	Yes	No	Yes	Nα	Yes	Low
40	Sauletzhanovna et al., 2024 [53]	Yes	Yes	Yes	Yes	Yes	Yes	Yes	Yes	Unclear	Yes	Yes	Low
41	Füller et al., 2025 [54]	Yes	Yes	Yes	No	No	Yes	Yes	Yes	Yes	No	Yes	Low
42	Malm et al., 2025 [55]	Yes	Yes	Yes	Yes	Yes	Yes	Yes	Yes	Yes	Yes	Yes	Low

References

1. Radley, A.; Grove, A.; Wright, S.; Thurston, H. Gender-Role Identity after Heart Attack: Links with Sex and Subjective Health Status. *Psychol. Health* **2000**, *15*, 123–133, doi:10.1080/08870440008400293.
2. Brink, E.; Karlson, B.W.; Hallberg, L.R.-M. Health Experiences of First-Time Myocardial Infarction: Factors Influencing Women's and Men's Health-Related Quality of Life after Five Months. *Psychol. Health Med.* **2002**, *7*, 5–16, doi:10.1080/13548500120101522.
3. McBurney, C.R.; Eagle, K.A.; Kline-Rogers, E.M.; Cooper, J.V.; Mani, O.C.M.; Smith, D.E.; Erickson, S.R. Health-Related Quality of Life in Patients 7 Months After a Myocardial Infarction: Factors Affecting the Short Form-12. *Pharmacother. J. Hum. Pharmacol. Drug Ther.* **2002**, *22*, 1616–1622, doi:10.1592/phco.22.17.1616.34121.
4. de Jong-Watt, W.; Sherifi, I. Patient-Centred Assessment of Social Support, Health Status and Quality of Life in Patients with Acute Coronary Syndrome. *Can. J. Cardiovasc. Nurs.* **2011**, *21*, 26–33.
5. Panthee, B.; Kritpracha, C.; Chinnawong, T. Correlation between Coping Strategies and Quality of Life among Myocardial Infarction Patients in Nepal. *Nurse Media J. Nurs.* **2011**, *1*, 187–194.
6. Brink, E. Considering Both Health-Promoting and Illness-Related Factors in Assessment of Health-Related Quality of Life After Myocardial Infarction. *Open Nurs. J.* **2012**, *6*, 90-94.
7. Sertoz, O.O.; Aydemir, O.; Gulpek, D.; Elbi, H.; Ozenli, Y.; Yilmaz, A.; Ozan, E.; Atesci, F.; Abay, E.; Semiz, M.; et al. The Impact of Physical and Psychological Comorbid Conditions on the Quality of Life of Patients with Acute Myocardial Infarction: A Multi-Center, Cross-Sectional Observational Study from Turkey. *Int. J. Psychiatry Med.* **2013**, *45*, 97–109, doi:10.2190/PM.45.2.a.
8. Mahesh, P.K.B.; Gunathunga, M.W.; Jayasinghe, S.; Arnold, S.M.; Haniffa, R.; De Silva, A.P. Pre-Event Quality of Life and Its Influence on the Post-Event Quality of Life among Patients with ST Elevation and Non-ST Elevation Myocardial Infarctions of a Premier Province of Sri Lanka. *Health Qual. Life Outcomes* **2017**, *15*, 154, doi:10.1186/s12955-017-0730-9.
9. Wulandari, D.; Ginanjar, A.S.; Purwono, U.; Purba, D. Marital Satisfaction, Anxiety, and Health-Related Quality of Life in Myocardial Infarction Patients. *J. Glob. Pharma Technol.* **2020**, *12*, 483-495.
10. Kang, K.; Gholizadeh, L.; Han, H.-R. Health-Related Quality of Life and Its Predictors in Korean Patients with Myocardial Infarction in the Acute Phase. *Clin. Nurs. Res.* **2021**, *30*, 161–170, doi:10.1177/1054773819894692.
11. Džubur, A.; Lisica, D.; Hodžić, E.; Begić, E.; Lepara, O.; Fajkić, A.; Gogić, E.; Ejubović, M. Relationship between Depression and Quality of Life after Myocardial Infarction. *Med. Glas.* **2022**, *19*, 0–0, doi:10.17392/1404-21.
12. Rasmussen, A.A.; Fridlund, B.; Nielsen, K.; Rasmussen, T.B.; Thrysoee, L.; Borregaard, B.; Thorup, C.B.; Berg, S.K.; Mols, R.E. Gender Differences in Patient-Reported Outcomes in Patients with Acute Myocardial Infarction. *Eur. J. Cardiovasc. Nurs.* **2022**, *21*, 772–781, doi:10.1093/eurjcn/zvac022.
13. Upadhyay, V.; Bhandari, S.S.; Rai, D.P.; Dutta, S.; García-Grau, P.; Vaddiparti, K. Improving Depression and Perceived Social Support Enhances Overall Quality of Life among Myocardial Infarction Survivors: Necessity for Integrating Mental Health Care into Cardiac Rehabilitation Programs. *Egypt. J. Neurol. Psychiatry Neurosurg.* **2022**, *58*, 87, doi:10.1186/s41983-022-00521-6.
14. Bogg, J.; Thornton, E.; Bundred, P. Gender Variability in Mood, Quality of Life and Coping Following Primary Myocardial Infarction. *Coron. Health Care* **2000**, *4*, 163–168, doi:10.1054/chec.2000.0095.

15. Fritz, H.L. Gender-Linked Personality Traits Predict Mental Health and Functional Status Following a First Coronary Event. *Health Psychol.* **2000**, *19*, 420-428, doi: 10.1037/0278-6133.19.5.420.
16. Lane, D.; Carroll, D.; Ring, C.; Beevers, D.G.; Lip, G.Y.H. Effects of Depression and Anxiety on Mortality and Quality-of-Life 4 Months after Myocardial Infarction. *J. Psychosom. Res.* **2000**, *49*, 229–238, doi:10.1016/S0022-3999(00)00170-7.
17. Mayou, R.A.; Gill, D.; Thompson, D.R.; Day, A.; Hicks, N.; Volmink, J.; Neil, A. Depression and Anxiety As Predictors of Outcome After Myocardial Infarction: *Psychosom. Med.* **2000**, *62*, 212–219, doi:10.1097/00006842-200003000-00011.
18. Beck, C.A.; Joseph, L.; Bélisle, P.; Pilote, L. Predictors of Quality of Life 6 Months and 1 Year after Acute Myocardial Infarction. *Am. Heart J.* **2001**, *142*, 271–279, doi:10.1067/mhj.2001.116758.
19. Lane, D.; Carroll, D.; Ring, C.; Beevers, D.G.; Lip, G.Y.H. Mortality and Quality of Life 12 Months After Myocardial Infarction: Effects of Depression and Anxiety: *Psychosom. Med.* **2001**, *63*, 221–230, doi:10.1097/00006842-200103000-00005.
20. Rumsfeld, J.S.; Magid, D.J.; Plomondon, M.E.; O'Brien, M.M.; Spertus, J.A.; Every, N.R.; Sales, A.E. Predictors of Quality of Life Following Acute Coronary Syndromes. *Am. J. Cardiol.* **2001**, *88*, 781–784, doi:10.1016/S0002-9149(01)01852-5.
21. Rumsfeld, J.S.; Magid, D.J.; Plomondon, M.E.; Sales, A.E.; Grunwald, G.K.; Every, N.R.; Spertus, J.A. History of Depression, Angina, and Quality of Life after Acute Coronary Syndromes. *Am. Heart J.* **2003**, *145*, 493–499, doi:10.1067/mhj.2003.177.
22. Bengtsson, I.; Hagman, M.; Währborg, P.; Wedel, H. Lasting Impact on Health-Related Quality of Life after a First Myocardial Infarction. *Int. J. Cardiol.* **2004**, *97*, 509–516, doi:10.1016/j.ijcard.2003.12.011.
23. Brink, E.; Grankvist, G.; Karlson, B.W.; Hallberg, L.R.-M. Health-Related Quality of Life in Women and Men One Year after Acute Myocardial Infarction. *Qual. Life Res.* **2005**, *14*, 749–757, doi:10.1007/s11136-004-0785-z.
24. Fauerbach, J.A.; Bush, D.E.; Thombs, B.D.; McCann, U.D.; Fogel, J.; Ziegelstein, R.C. Depression Following Acute Myocardial Infarction: A Prospective Relationship With Ongoing Health and Function. *Psychosomatics* **2005**, *46*, 355–361, doi:10.1176/appi.psy.46.4.355.
25. Spertus, J.; Safley, D.; Garg, M.; Jones, P.; Peterson, E.D. The Influence of Race on Health Status Outcomes One Year After an Acute Coronary Syndrome. *J. Am. Coll. Cardiol.* **2005**, *46*, 1838–1844, doi:10.1016/j.jacc.2005.05.092.
26. Dickens, C.M.; McGowan, L.; Percival, C.; Tomenson, B.; Cotter, L.; Heagerty, A.; Creed, F.H. Contribution of Depression and Anxiety to Impaired Health-Related Quality of Life Following First Myocardial Infarction. *Br. J. Psychiatry* **2006**, *189*, 367–372, doi:10.1192/bjp.bp.105.018234.
27. De Jonge, P.; Spijkerman, T.A.; Van Den Brink, R.H.S.; Ormel, J. Depression after Myocardial Infarction Is a Risk Factor for Declining Health Related Quality of Life and Increased Disability and Cardiac Complaints at 12 Months. *Heart* **2006**, *92*, 32–39, doi:10.1136/hrt.2004.059451.
28. Peterson, P.N.; Spertus, J.A.; Magid, D.J.; Masoudi, F.A.; Reid, K.; Hamman, R.F.; Rumsfeld, J.S. The Impact of Diabetes on One-Year Health Status Outcomes Following Acute Coronary Syndromes. *BMC Cardiovasc. Disord.* **2006**, *6*, 41, doi:10.1186/1471-2261-6-41.
29. Failde, I.I.; Soto, M.M. Changes in Health Related Quality of Life 3 Months after an Acute Coronary Syndrome. *BMC Public Health* **2006**, *6*, 18, doi:10.1186/1471-2458-6-18.
30. Norris, C.M.; Hegadoren, K.; Pilote, L. Depression Symptoms Have a Greater Impact on the 1-Year Health-Related Quality of Life Outcomes of Women Post-Myocardial Infarction Compared to Men. *Eur. J. Cardiovasc. Nurs.* **2007**, *6*, 92–98, doi:10.1016/j.ejcnurse.2006.05.003.

31. Rahimi, A.R.; Spertus, J.A.; Reid, K.J.; Bernheim, S.M.; Krumholz, H.M. Financial Barriers to Health Care and Outcomes After Acute Myocardial Infarction. *JAMA* **2007**, *297*, 1063, doi:10.1001/jama.297.10.1063.
32. Ho, P.M.; Eng, M.H.; Rumsfeld, J.S.; Spertus, J.A.; Peterson, P.N.; Jones, P.G.; Peterson, E.D.; Alexander, K.P.; Havranek, E.P.; Krumholz, H.M.; et al. The Influence of Age on Health Status Outcomes after Acute Myocardial Infarction. *Am. Heart J.* **2008**, *155*, 855–861, doi:10.1016/j.ahj.2007.11.032.
33. Thombs, B.D.; Ziegelstein, R.C.; Stewart, D.E.; Abbey, S.E.; Parakh, K.; Grace, S.L. Usefulness of Persistent Symptoms of Depression to Predict Physical Health Status 12 Months After an Acute Coronary Syndrome. *Am. J. Cardiol.* **2008**, *101*, 15–19, doi:10.1016/j.amjcard.2007.07.043.
34. Arnold, S.V.; Alexander, K.P.; Masoudi, F.A.; Ho, P.M.; Xiao, L.; Spertus, J.A. The Effect of Age on Functional and Mortality Outcomes After Acute Myocardial Infarction. *J. Am. Geriatr. Soc.* **2009**, *57*, 209–217, doi:10.1111/j.1532-5415.2008.02106.x.
35. Bergman, E.; Malm, D.; Karlsson, J.-E.; Berterö, C. Longitudinal Study of Patients after Myocardial Infarction: Sense of Coherence, Quality of Life, and Symptoms. *Heart Lung* **2009**, *38*, 129–140, doi:10.1016/j.hrtlng.2008.05.007.
36. Spertus, J.A.; Jones, P.G.; Masoudi, F.A.; Rumsfeld, J.S.; Krumholz, H.M. Factors Associated With Racial Differences in Myocardial Infarction Outcomes. *Ann. Intern. Med.* **2009**, *150*, 314–324, doi:10.7326/0003-4819-150-5-200903030-00007.
37. Arnold, S.V.; Spertus, J.A.; Jones, P.G.; Xiao, L.; Cohen, D.J. The Impact of Dyspnea on Health-Related Quality of Life in Patients with Coronary Artery Disease: Results from the PREMIER Registry. *Am. Heart J.* **2009**, *157*, 1042-1049.e1, doi:10.1016/j.ahj.2009.03.021.
38. Leifheit-Limson, E.C.; Reid, K.J.; Kasl, S.V.; Lin, H.; Jones, P.G.; Buchanan, D.M.; Parashar, S.; Peterson, P.N.; Spertus, J.A.; Lichtman, J.H. The Role of Social Support in Health Status and Depressive Symptoms After Acute Myocardial Infarction: Evidence for a Stronger Relationship Among Women. *Circ. Cardiovasc. Qual. Outcomes* **2010**, *3*, 143–150, doi:10.1161/CIRCOUTCOMES.109.899815.
39. Shin, N.-M.; Choi, J. Relationship Between Survivors' Perceived Health Status Following Acute Coronary Syndrome and Depression Symptoms During Early Recovery Phase. *Asian Nurs. Res.* **2010**, *4*, 174–184, doi:10.1016/S1976-1317(11)60002-9.
40. Bucholz, E.M.; Rathore, S.S.; Gosch, K.; Schoenfeld, A.; Jones, P.G.; Buchanan, D.M.; Spertus, J.A.; Krumholz, H.M. Effect of Living Alone on Patient Outcomes After Hospitalization for Acute Myocardial Infarction. *Am. J. Cardiol.* **2011**, *108*, 943–948, doi:10.1016/j.amjcard.2011.05.023.
41. Dueñas, M.; Ramirez, C.; Arana, R.; Failde, I. Gender Differences and Determinants of Health Related Quality of Life in Coronary Patients: A Follow-up Study. *BMC Cardiovasc. Disord.* **2011**, *11*, 24, doi:10.1186/1471-2261-11-24.
42. Ginzburg, K.; Ein-Dor, T. Posttraumatic Stress Syndromes and Health-Related Quality of Life Following Myocardial Infarction: 8-Year Follow-Up. *Gen. Hosp. Psychiatry* **2011**, *33*, 565–571, doi:10.1016/j.genhosppsych.2011.08.015.
43. Brink, E.; Alsén, P.; Herlitz, J.; Kjellgren, K.; Cliffordson, C. General Self-Efficacy and Health-Related Quality of Life after Myocardial Infarction. *Psychol. Health Med.* **2012**, *17*, 346–355, doi:10.1080/13548506.2011.608807.
44. Leifheit-Limson, E.C.; Reid, K.J.; Kasl, S.V.; Lin, H.; Buchanan, D.M.; Jones, P.G.; Peterson, P.N.; Parashar, S.; Spertus, J.A.; Lichtman, J.H. Changes in Social Support within the Early Recovery Period and Outcomes after Acute Myocardial Infarction. *J. Psychosom. Res.* **2012**, *73*, 35–41, doi:10.1016/j.jpsychores.2012.04.006.
45. Williams, L.; O'Connor, R.C.; Grubb, N.R.; O'Carroll, R.E. Type D Personality and Three-Month Psychosocial Outcomes among Patients Post-Myocardial Infarction. *J. Psychosom. Res.* **2012**, *72*, 422–426, doi:10.1016/j.jpsychores.2012.02.007.

46. Hosseini, S.H.; Ghaemian, A.; Mehdizadeh, E.; Ashraf, H. Contribution of Depression and Anxiety to Impaired Quality of Life in Survivors of Myocardial Infarction. *Int. J. Psychiatry Clin. Pract.* **2014**, *18*, 175–181, doi:10.3109/13651501.2014.940049.
47. Bennett, K.K.; Buchanan, D.M.; Jones, P.G.; Spertus, J.A. Socioeconomic Status, Cognitive-Emotional Factors, and Health Status Following Myocardial Infarction: Testing the Reserve Capacity Model. *J. Behav. Med.* **2015**, *38*, 110–121, doi:10.1007/s10865-014-9583-4.
48. Salazar, A.; Dueñas, M.; Fernandez-Palacin, F.; Failde, I. Factors Related to the Evolution of Health Related Quality of Life in Coronary Patients. A Longitudinal Approach Using Weighted Generalized Estimating Equations with Missing Data. *Int. J. Cardiol.* **2016**, *223*, 940–946, doi:10.1016/j.ijcard.2016.08.300.
49. Dzubur, A.; Mekic, M.; Pesto, S.; Nabil, N. Echocardiographic Parameters as Life Quality Predictors in Patients After Myocardial Infarction Treated with Different Methods. *Med. Arch.* **2016**, *70*, 419, doi:10.5455/medarh.2016.70.419-424.
50. Kang, K.; Gholizadeh, L.; Han, H.-R.; Inglis, S.C. Predictors of Health-Related Quality of Life in Korean Patients with Myocardial Infarction: A Longitudinal Observational Study. *Heart Lung* **2018**, *47*, 142–148, doi:10.1016/j.hrtlng.2017.12.005.
51. Xia, K.; Wang, L.-F.; Yang, X.-C.; Jiang, H.-Y.; Zhang, L.-J.; Yao, D.-K.; Hu, D.-Y.; Ding, R.-J. Comparing the Effects of Depression, Anxiety, and Comorbidity on Quality-of-Life, Adverse Outcomes, and Medical Expenditure in Chinese Patients with Acute Coronary Syndrome. *Chin. Med. J. (Engl.)* **2019**, *132*, 1045–1052, doi:10.1097/CM9.0000000000000215.
52. Jlassi, O.; Omrane, A.; Ben Massoud, M.; Khalfallah, T.; Bouzgarrou, L.; Gamra, H. Determinants of Health-Related Quality of Life among Patients with Ischemic Heart Disease. *Health Syst.* **2024**, *13*, 322–331, doi:10.1080/20476965.2023.2275799.
53. Sauletzhanovna, T.A.; Mohammed, W.K.; Ahmed, A.S.; Mohammed, H.I.; Al-Hili, A.; Alnajar, M.J.; Naser, N.S.; Amr, E.F.; Mohsin, R.M. The Predictive Value of Depression and Anxiety on Protracted Cardiovascular Outcomes in Individuals with Acute Myocardial Infarction. *Int. J. Body Mind Cult.* **2024**, 64–75, doi:10.22122/ijbmc.v11isp.739.
54. Füller, D.; Andresen-Bundus, H.; Pagonas, N.; Jaehn, P.; Ukena, C.; Gödde, K.; Holmberg, C.; Ritter, O.; Sasko, B. Adverse Socioeconomic Factors Are Associated with a Widening Gap in One-Year Health-Related Quality of Life after Acute Myocardial Infarction. *Sci. Rep.* **2025**, *15*, 19791, doi:10.1038/s41598-025-04604-1.
55. Malm, D.; Mårtensson, J.; Årestedt, K. Sense of Coherence and Quality of Life in the Recovery of Women and Men with Myocardial Infarction: A 10-Year Follow-up Study. *Eur. J. Cardiovasc. Nurs.* **2025**, *24*, 631–639, doi:10.1093/eurjcn/zvaf028.
