# Supplementary material for: Determinants of Health-Related Quality of Life After Acute Coronary Syndromes: A Systematic Review
Source: Healthcare (Basel). 2026 May 9;14(10):1292. doi: 10.3390/healthcare14101292 (PMC13205943; doi:10.3390/healthcare14101292)
Supplement: Supplementary file 1 [file healthcare-14-01292-s001.zip › Supplementary Table S2.pdf]

Supplementary Table S2. Frequency of determinants of health-related quality of life after acute coronary syndromes.

	Determinants	No of studies	References
**Demographic factors**			
**1**	Sex	23	[1–23]
**2**	Age	19	[2,5,24–26,8,27,9,10,28,29,12,30,16–20,31]
**3**	Race (Non-Caucasian vs Caucasian)	3	[24,10,32]
**4**	Race (black vs whites)	2	[33,34]
**Socioeconomic factors**			
**5**	Educational level	6	[13,18,19,35–37]
**6**	Employment status	5	[3,5,6,27]
**7**	Financial status	6	[38,32,39,35–37]
**8**	Social support	5	[15,39,40,31,41]
**9**	Marital/partner status	4	[3,6,20]
**10**	Living alone	3	[3,6,42]
**11**	Marital satisfaction	1	[21]
**12**	Occupational qualifications	1	[37]
**13**	Insurance status (yes)	1	[35]
**14**	Reserves at the end of month	1	[35]
**15**	Number of children in charge	1	[43]
**16**	Socioeconomic status	1	[35]
**17**	Return to work	1	[30]
**Patient behaviors**			
**18**	Previous exercise behavior	3	[3,6,20]
**19**	Smoking	2	[27,43]
**20**	History of alcohol or substance abuse	1	[10]
**21**	Adherence to prescribed medications	1	[25]
**Psychological factors**			
**22**	Depression	25	[1,3,44,5,6,24,45,7,8,46,27,9,12,13,32,17–19,35,31,47,36,22,41,48]
**23**	Anxiety	8	[1,3,44,6,27,47,21,48]
**24**	Fatigue	3	[8,16,17]
**25**	Disturbed sleep	1	[16]
**26**	Acute stress disorder	1	[49]
**27**	Posttraumatic Stress Disorder	1	[49]
**28**	Perceived stress	1	[35]
**Personality traits and coping strategies**			
**29**	Sense of coherence	3	[14,17,50]
**30**	Coping strategies	3	[1,7,51]
**31**	Agency	1	[2]
**32**	Unmitigated communion	1	[2]
**33**	Level of masculinity	1	[4]
**34**	Understanding of illness	1	[31]
**35**	Optimism	1	[17]

**36**	Type D personality	1	[52]
**37**	Self-efficacy	1	[16]
**38**	Reserve capacity	1	[35]
**39**	Negative mood	1	[1]
**Quality of life and dimensions**			
**40**	Baseline quality of life	6	[13,5,9,27,12,53]
**41**	Mental health	4	[32,30,16,20]
**42**	Physical health	3	[13,32,35]
**Health System factor**			
**43**	Access to care	1	[13]
**Clinical factors and factors related to Acute Coronary Syndromes**			
**44**	Left Ventricular Ejection Fraction (LVEF)	5	[10,13,52,54,31]
**45**	Revascularization in the post discharge interim	5	[24,26,28,30,51]
**46**	Length of hospital stay	3	[3,6,43]
**47**	Presence of cardiac symptoms	3	[4,7,30]
**48**	Prior Coronary Artery Bypass Graft surgery	3	[5,24,19]
**49**	Severity of infarction	3	[3,5,6]
**50**	"ST" Elevation Myocardial Infarction vs "Non-ST" Elevation Myocardial Infarction	2	[53,31]
**51**	Unstable angina vs Acute Myocardial Infarction	2	[24,30]
**52**	Previous Myocardial Infarction	2	[9,10]
**53**	Time passed after myocardial infarction	2	[20,30]
**54**	Rehospitalization after index hospitalization for Myocardial Infarction	2	[25,30]
**55**	Localization of the index Myocardial Infarction	1	[27]
**56**	Revascularization during initial hospitalization	1	[24]
**57**	History of Percutaneous Coronary Intervention	1	[25]
**58**	Cardiac events during follow up period	1	[27]
**59**	Subsequent Myocardial Infarction	1	[51]
**60**	Thrombolytic therapy	2	[10,19]
**61**	Percutaneous Coronary Intervention vs medications	1	[54]
**62**	Coronary Artery Bypass Graft vs delayed Percutaneous Coronary Intervention	1	[54]
**63**	Left Ventricular Internal Diameter at end diastole (LVIDd)	1	[54]
**64**	Mitral regurtation	1	[54]
**65**	Discharge prescription of an angiotensin converting enzyme inhibitor or an	1	[10]

	angiotensin receptor blocker		
**66**	Acute mitral regurgitation, acute ventriculoseptal defect, or tamponade	1	[5]
**67**	Q wave Myocardial Infarction	1	[19]
**68**	Dyspnea	1	[55]
**69**	Shock sustained in-hospital	1	[5]
**70**	Stress test not performed	1	[24]
**71**	Creatine kinase level	1	[5]
**Risk factors for ACS and patient comorbidities**			
**72**	Comorbidities	6	[4,25,27,18,47,30]
**73**	Diabetes	5	[5,24,10,36,43]
**74**	Previous stroke	4	[24,25,19,36]
**75**	Heart failure	3	[24,25,13]
**76**	History of Coronary Heart Disease	3	[28,30,20]
**77**	Hypertension	2	[18,20]
**78**	Gastrointestinal problems	1	[27]
**79**	Rheumatic disease	1	[27]
**80**	Chronic Obstructive Pulmonary Disease	1	[24]
**81**	Chronic lung disease	1	[10]
**82**	Elevated serum creatinine	1	[24]
**83**	Anemia	1	[13]
**84**	History of arthritis	1	[24]
**85**	History of other heart disease	1	[36]
**86**	Hypercholesterolemia	1	[19]
**87**	Peptic ulcer disease	1	[24]

References

1. Bogg, J.; Thornton, E.; Bundred, P. Gender Variability in Mood, Quality of Life and Coping Following Primary Myocardial Infarction. *Coron. Health Care* **2000**, *4*, 163–168, doi:10.1054/chec.2000.0095.
2. Fritz, H.L. Gender-Linked Personality Traits Predict Mental Health and Functional Status Following a First Coronary Event. *Health Psychol.* **2000**, *19*, 420-428, doi: 10.1037/0278-6133.19.5.420.
3. Lane, D.; Carroll, D.; Ring, C.; Beevers, D.G.; Lip, G.Y.H. Effects of Depression and Anxiety on Mortality and Quality-of-Life 4 Months after Myocardial Infarction. *J. Psychosom. Res.* **2000**, *49*, 229–238, doi:10.1016/S0022-3999(00)00170-7.
4. Radley, A.; Grove, A.; Wright, S.; Thurston, H. Gender-Role Identity after Heart Attack: Links with Sex and Subjective Health Status. *Psychol. Health* **2000**, *15*, 123–133, doi:10.1080/08870440008400293.
5. Beck, C.A.; Joseph, L.; Bélisle, P.; Pilote, L. Predictors of Quality of Life 6 Months and 1 Year after Acute Myocardial Infarction. *Am. Heart J.* **2001**, *142*, 271–279, doi:10.1067/mhj.2001.116758.
6. Lane, D.; Carroll, D.; Ring, C.; Beevers, D.G.; Lip, G.Y.H. Mortality and Quality of Life 12 Months After Myocardial Infarction: Effects of Depression and Anxiety: *Psychosom. Med.* **2001**, *63*, 221–230, doi:10.1097/00006842-200103000-00005.
7. Brink, E.; Karlson, B.W.; Hallberg, L.R.-M. Health Experiences of First-Time Myocardial Infarction: Factors Influencing Women's and Men's Health-Related Quality of Life after Five Months. *Psychol. Health Med.* **2002**, *7*, 5–16, doi:10.1080/13548500120101522.

8. Brink, E.; Grankvist, G.; Karlson, B.W.; Hallberg, L.R.-M. Health-Related Quality of Life in Women and Men One Year after Acute Myocardial Infarction. *Qual. Life Res.* **2005**, *14*, 749–757, doi:10.1007/s11136-004-0785-z.
9. De Jonge, P.; Spijkerman, T.A.; Van Den Brink, R.H.S.; Ormel, J. Depression after Myocardial Infarction Is a Risk Factor for Declining Health Related Quality of Life and Increased Disability and Cardiac Complaints at 12 Months. *Heart* **2006**, *92*, 32–39, doi:10.1136/hrt.2004.059451.
10. Peterson, P.N.; Spertus, J.A.; Magid, D.J.; Masoudi, F.A.; Reid, K.; Hamman, R.F.; Rumsfeld, J.S. The Impact of Diabetes on One-Year Health Status Outcomes Following Acute Coronary Syndromes. *BMC Cardiovasc. Disord.* **2006**, *6*, 41, doi:10.1186/1471-2261-6-41.
11. Norris, C.M.; Hegadoren, K.; Pilote, L. Depression Symptoms Have a Greater Impact on the 1-Year Health-Related Quality of Life Outcomes of Women Post-Myocardial Infarction Compared to Men. *Eur. J. Cardiovasc. Nurs.* **2007**, *6*, 92–98, doi:10.1016/j.ejcnurse.2006.05.003.
12. Thombs, B.D.; Ziegelstein, R.C.; Stewart, D.E.; Abbey, S.E.; Parakh, K.; Grace, S.L. Usefulness of Persistent Symptoms of Depression to Predict Physical Health Status 12 Months After an Acute Coronary Syndrome. *Am. J. Cardiol.* **2008**, *101*, 15–19, doi:10.1016/j.amjcard.2007.07.043.
13. Arnold, S.V.; Alexander, K.P.; Masoudi, F.A.; Ho, P.M.; Xiao, L.; Spertus, J.A. The Effect of Age on Functional and Mortality Outcomes After Acute Myocardial Infarction. *J. Am. Geriatr. Soc.* **2009**, *57*, 209–217, doi:10.1111/j.1532-5415.2008.02106.x.
14. Bergman, E.; Malm, D.; Karlsson, J.-E.; Berterö, C. Longitudinal Study of Patients after Myocardial Infarction: Sense of Coherence, Quality of Life, and Symptoms. *Heart Lung* **2009**, *38*, 129–140, doi:10.1016/j.hrtlng.2008.05.007.
15. Leifheit-Limson, E.C.; Reid, K.J.; Kasl, S.V.; Lin, H.; Jones, P.G.; Buchanan, D.M.; Parashar, S.; Peterson, P.N.; Spertus, J.A.; Lichtman, J.H. The Role of Social Support in Health Status and Depressive Symptoms After Acute Myocardial Infarction: Evidence for a Stronger Relationship Among Women. *Circ. Cardiovasc. Qual. Outcomes* **2010**, *3*, 143–150, doi:10.1161/CIRCOUTCOMES.109.899815.
16. Brink, E.; Alsén, P.; Herlitz, J.; Kjellgren, K.; Cliffordson, C. General Self-Efficacy and Health-Related Quality of Life after Myocardial Infarction. *Psychol. Health Med.* **2012**, *17*, 346–355, doi:10.1080/13548506.2011.608807.
17. Brink, E. Considering Both Health-Promoting and Illness-Related Factors in Assessment of Health-Related Quality of Life After Myocardial Infarction. *Open Nurs. J.* **2012**, *6*, 90-94.
18. Sertoz, O.O.; Aydemir, O.; Gulpek, D.; Elbi, H.; Ozenli, Y.; Yilmaz, A.; Ozan, E.; Atesci, F.; Abay, E.; Semiz, M.; et al. The Impact of Physical and Psychological Comorbid Conditions on the Quality of Life of Patients with Acute Myocardial Infarction: A Multi-Center, Cross-Sectional Observational Study from Turkey. *Int. J. Psychiatry Med.* **2013**, *45*, 97–109, doi:10.2190/PM.45.2.a.
19. Hosseini, S.H.; Ghaemian, A.; Mehdizadeh, E.; Ashraf, H. Contribution of Depression and Anxiety to Impaired Quality of Life in Survivors of Myocardial Infarction. *Int. J. Psychiatry Clin. Pract.* **2014**, *18*, 175–181, doi:10.3109/13651501.2014.940049.
20. Salazar, A.; Dueñas, M.; Fernandez-Palacin, F.; Failde, I. Factors Related to the Evolution of Health Related Quality of Life in Coronary Patients. A Longitudinal Approach Using Weighted Generalized Estimating Equations with Missing Data. *Int. J. Cardiol.* **2016**, *223*, 940–946, doi:10.1016/j.ijcard.2016.08.300.
21. Wulandari, D.; Ginanjar, A.S.; Purwono, U.; Purba, D. Marital Satisfaction, Anxiety, and Health-Related Quality of Life in Myocardial Infarction Patients. *J. Glob. Pharma Technol.* **2020**, *12*, 483-495.
22. Džubur, A.; Lisica, D.; Hodžić, E.; Begić, E.; Lepara, O.; Fajkić, A.; Gogić, E.; Ejubović, M. Relationship between Depression and Quality of Life after Myocardial Infarction. *Med. Glas.* **2022**, *19*, 0–0, doi:10.17392/1404-21.

23. Rasmussen, A.A.; Fridlund, B.; Nielsen, K.; Rasmussen, T.B.; Thrysoee, L.; Borregaard, B.; Thorup, C.B.; Berg, S.K.; Mols, R.E. Gender Differences in Patient-Reported Outcomes in Patients with Acute Myocardial Infarction. *Eur. J. Cardiovasc. Nurs.* **2022**, *21*, 772–781, doi:10.1093/eurjcn/zvac022.
24. Rumsfeld, J.S.; Magid, D.J.; Plomondon, M.E.; O'Brien, M.M.; Spertus, J.A.; Every, N.R.; Sales, A.E. Predictors of Quality of Life Following Acute Coronary Syndromes. *Am. J. Cardiol.* **2001**, *88*, 781–784, doi:10.1016/S0002-9149(01)01852-5.
25. McBurney, C.R.; Eagle, K.A.; Kline-Rogers, E.M.; Cooper, J.V.; Mani, O.C.M.; Smith, D.E.; Erickson, S.R. Health-Related Quality of Life in Patients 7 Months After a Myocardial Infarction: Factors Affecting the Short Form-12. *Pharmacother. J. Hum. Pharmacol. Drug Ther.* **2002**, *22*, 1616–1622, doi:10.1592/phco.22.17.1616.34121.
26. Bengtsson, I.; Hagman, M.; Währborg, P.; Wedel, H. Lasting Impact on Health-Related Quality of Life after a First Myocardial Infarction. *Int. J. Cardiol.* **2004**, *97*, 509–516, doi:10.1016/j.ijcard.2003.12.011.
27. Dickens, C.M.; McGowan, L.; Percival, C.; Tomenson, B.; Cotter, L.; Heagerty, A.; Creed, F.H. Contribution of Depression and Anxiety to Impaired Health-Related Quality of Life Following First Myocardial Infarction. *Br. J. Psychiatry* **2006**, *189*, 367–372, doi:10.1192/bjp.bp.105.018234.
28. Failde, I.I.; Soto, M.M. Changes in Health Related Quality of Life 3 Months after an Acute Coronary Syndrome. *BMC Public Health* **2006**, *6*, 18, doi:10.1186/1471-2458-6-18.
29. Ho, P.M.; Eng, M.H.; Rumsfeld, J.S.; Spertus, J.A.; Peterson, P.N.; Jones, P.G.; Peterson, E.D.; Alexander, K.P.; Havranek, E.P.; Krumholz, H.M.; et al. The Influence of Age on Health Status Outcomes after Acute Myocardial Infarction. *Am. Heart J.* **2008**, *155*, 855–861, doi:10.1016/j.ahj.2007.11.032.
30. Dueñas, M.; Ramirez, C.; Arana, R.; Failde, I. Gender Differences and Determinants of Health Related Quality of Life in Coronary Patients: A Follow-up Study. *BMC Cardiovasc. Disord.* **2011**, *11*, 24, doi:10.1186/1471-2261-11-24.
31. Kang, K.; Gholizadeh, L.; Han, H.-R.; Inglis, S.C. Predictors of Health-Related Quality of Life in Korean Patients with Myocardial Infarction: A Longitudinal Observational Study. *Heart Lung* **2018**, *47*, 142–148, doi:10.1016/j.hrtlng.2017.12.005.
32. Shin, N.-M.; Choi, J. Relationship Between Survivors' Perceived Health Status Following Acute Coronary Syndrome and Depression Symptoms During Early Recovery Phase. *Asian Nurs. Res.* **2010**, *4*, 174–184, doi:10.1016/S1976-1317(11)60002-9.
33. Spertus, J.; Safley, D.; Garg, M.; Jones, P.; Peterson, E.D. The Influence of Race on Health Status Outcomes One Year After an Acute Coronary Syndrome. *J. Am. Coll. Cardiol.* **2005**, *46*, 1838–1844, doi:10.1016/j.jacc.2005.05.092.
34. Spertus, J.A.; Jones, P.G.; Masoudi, F.A.; Rumsfeld, J.S.; Krumholz, H.M. Factors Associated With Racial Differences in Myocardial Infarction Outcomes. *Ann. Intern. Med.* **2009**, *150*, 314–324, doi:10.7326/0003-4819-150-5-200903030-00007.
35. Bennett, K.K.; Buchanan, D.M.; Jones, P.G.; Spertus, J.A. Socioeconomic Status, Cognitive-Emotional Factors, and Health Status Following Myocardial Infarction: Testing the Reserve Capacity Model. *J. Behav. Med.* **2015**, *38*, 110–121, doi:10.1007/s10865-014-9583-4.
36. Kang, K.; Gholizadeh, L.; Han, H.-R. Health-Related Quality of Life and Its Predictors in Korean Patients with Myocardial Infarction in the Acute Phase. *Clin. Nurs. Res.* **2021**, *30*, 161–170, doi:10.1177/1054773819894692.
37. Füller, D.; Andresen-Bundus, H.; Pagonas, N.; Jaehn, P.; Ukena, C.; Gödde, K.; Holmberg, C.; Ritter, O.; Sasko, B. Adverse Socioeconomic Factors Are Associated with a Widening Gap in One-Year Health-Related Quality of Life after Acute Myocardial Infarction. *Sci. Rep.* **2025**, *15*, 19791, doi:10.1038/s41598-025-04604-1.

38. Rahimi, A.R.; Spertus, J.A.; Reid, K.J.; Bernheim, S.M.; Krumholz, H.M. Financial Barriers to Health Care and Outcomes After Acute Myocardial Infarction. *JAMA* **2007**, *297*, 1063, doi:10.1001/jama.297.10.1063.
39. de Jong-Watt, W.; Sherifi, I. Patient-Centred Assessment of Social Support, Health Status and Quality of Life in Patients with Acute Coronary Syndrome. *Can. J. Cardiovasc. Nurs.* **2011**, *21*, 26–33.
40. Leifheit-Limson, E.C.; Reid, K.J.; Kasl, S.V.; Lin, H.; Buchanan, D.M.; Jones, P.G.; Peterson, P.N.; Parashar, S.; Spertus, J.A.; Lichtman, J.H. Changes in Social Support within the Early Recovery Period and Outcomes after Acute Myocardial Infarction. *J. Psychosom. Res.* **2012**, *73*, 35–41, doi:10.1016/j.jpsychores.2012.04.006.
41. Upadhyay, V.; Bhandari, S.S.; Rai, D.P.; Dutta, S.; García-Grau, P.; Vaddiparti, K. Improving Depression and Perceived Social Support Enhances Overall Quality of Life among Myocardial Infarction Survivors: Necessity for Integrating Mental Health Care into Cardiac Rehabilitation Programs. *Egypt. J. Neurol. Psychiatry Neurosurg.* **2022**, *58*, 87, doi:10.1186/s41983-022-00521-6.
42. Bucholz, E.M.; Rathore, S.S.; Gosch, K.; Schoenfeld, A.; Jones, P.G.; Buchanan, D.M.; Spertus, J.A.; Krumholz, H.M. Effect of Living Alone on Patient Outcomes After Hospitalization for Acute Myocardial Infarction. *Am. J. Cardiol.* **2011**, *108*, 943–948, doi:10.1016/j.amjcard.2011.05.023.
43. Jlassi, O.; Omrane, A.; Ben Massoud, M.; Khalfallah, T.; Bouzgarrou, L.; Gamra, H. Determinants of Health-Related Quality of Life among Patients with Ischemic Heart Disease. *Health Syst.* **2024**, *13*, 322–331, doi:10.1080/20476965.2023.2275799.
44. Mayou, R.A.; Gill, D.; Thompson, D.R.; Day, A.; Hicks, N.; Volmink, J.; Neil, A. Depression and Anxiety As Predictors of Outcome After Myocardial Infarction: *Psychosom. Med.* **2000**, *62*, 212–219, doi:10.1097/00006842-200003000-00011.
45. Rumsfeld, J.S.; Magid, D.J.; Plomondon, M.E.; Sales, A.E.; Grunwald, G.K.; Every, N.R.; Spertus, J.A. History of Depression, Angina, and Quality of Life after Acute Coronary Syndromes. *Am. Heart J.* **2003**, *145*, 493–499, doi:10.1067/mhj.2003.177.
46. Fauerbach, J.A.; Bush, D.E.; Thombs, B.D.; McCann, U.D.; Fogel, J.; Ziegelstein, R.C. Depression Following Acute Myocardial Infarction: A Prospective Relationship With Ongoing Health and Function. *Psychosomatics* **2005**, *46*, 355–361, doi:10.1176/appi.psy.46.4.355.
47. Xia, K.; Wang, L.-F.; Yang, X.-C.; Jiang, H.-Y.; Zhang, L.-J.; Yao, D.-K.; Hu, D.-Y.; Ding, R.-J. Comparing the Effects of Depression, Anxiety, and Comorbidity on Quality-of-Life, Adverse Outcomes, and Medical Expenditure in Chinese Patients with Acute Coronary Syndrome. *Chin. Med. J. (Engl.)* **2019**, *132*, 1045–1052, doi:10.1097/CM9.0000000000000215.
48. Sauletzhanovna, T.A.; Mohammed, W.K.; Ahmed, A.S.; Mohammed, H.I.; Al-Hili, A.; Alnajar, M.J.; Naser, N.S.; Amr, E.F.; Mohsin, R.M. The Predictive Value of Depression and Anxiety on Protracted Cardiovascular Outcomes in Individuals with Acute Myocardial Infarction. *Int. J. Body Mind Cult.* **2024**, 64–75, doi:10.22122/ijbmc.v11isp.739.
49. Ginzburg, K.; Ein-Dor, T. Posttraumatic Stress Syndromes and Health-Related Quality of Life Following Myocardial Infarction: 8-Year Follow-Up. *Gen. Hosp. Psychiatry* **2011**, *33*, 565–571, doi:10.1016/j.genhosppsych.2011.08.015.
50. Malm, D.; Mårtensson, J.; Årestedt, K. Sense of Coherence and Quality of Life in the Recovery of Women and Men with Myocardial Infarction: A 10-Year Follow-up Study. *Eur. J. Cardiovasc. Nurs.* **2025**, *24*, 631–639, doi:10.1093/eurjcn/zvaf028.
51. Panthee, B.; Kritpracha, C.; Chinnawong, T. Correlation between Coping Strategies and Quality of Life among Myocardial Infarction Patients in Nepal. *Nurse Media J. Nurs.* **2011**, *1*, 187–194.
52. Williams, L.; O'Connor, R.C.; Grubb, N.R.; O'Carroll, R.E. Type D Personality and Three-Month Psychosocial Outcomes among Patients Post-Myocardial Infarction. *J. Psychosom. Res.* **2012**, *72*, 422–426, doi:10.1016/j.jpsychores.2012.02.007.

53. Mahesh, P.K.B.; Gunathunga, M.W.; Jayasinghe, S.; Arnold, S.M.; Haniffa, R.; De Silva, A.P. Pre-Event Quality of Life and Its Influence on the Post-Event Quality of Life among Patients with ST Elevation and Non-ST Elevation Myocardial Infarctions of a Premier Province of Sri Lanka. *Health Qual. Life Outcomes* **2017**, *15*, 154, doi:10.1186/s12955-017-0730-9.
54. Dzubur, A.; Mekic, M.; Pesto, S.; Nabil, N. Echocardiographic Parameters as Life Quality Predictors in Patients After Myocardial Infarction Treated with Different Methods. *Med. Arch.* **2016**, *70*, 419, doi:10.5455/medarh.2016.70.419-424.
55. Arnold, S.V.; Spertus, J.A.; Jones, P.G.; Xiao, L.; Cohen, D.J. The Impact of Dyspnea on Health-Related Quality of Life in Patients with Coronary Artery Disease: Results from the PREMIER Registry. *Am. Heart J.* **2009**, *157*, 1042-1049.e1, doi:10.1016/j.ahj.2009.03.021.
