# Supplementary material for: Determinants of Health-Related Quality of Life After Acute Coronary Syndromes: A Systematic Review
Source: Healthcare (Basel). 2026 May 9;14(10):1292. doi: 10.3390/healthcare14101292 (PMC13205943; doi:10.3390/healthcare14101292)
Supplement: Supplementary file 1 [file healthcare-14-01292-s001.zip › Supplementary Table S3.pdf]

Supplementary Table S3. Associations between determinants and health-related quality of life after acute coronary syndromes.

	Bogg et al., 2000 [1]	Fritz, 2000 [2]	Lane et al., 2000 [3]	Mayou et al., 2000 [4]	Radley et al., 2000 [5]	Beck et al., 2001 [6]	Lane et al., 2001 [7]	Rumsfeld et al., 2001 [8]	Brink et al., 2002 [9]	McBurney et al., 2002 [10]	Rumsfeld et al., 2003 [11]	Bengtsson et al., 2004 [12]	Brink et al., 2005 [13]	Fauerbach et al., 2005 [14]	Spertus et al., 2005 [15]	Dickens et al., 2006 [16]	de Jonge et al., 2006 [17]	Peterson et al., 2006 [18]	Failde & Soto, 2006 [19]	Norris et al., 2007 [20]	Rahimi et al., 2007 [21]	Ho et al., 2008 [22]	Thombs et al., 2008 [23]	Arnold et al., 2009 [24]	Bergman et al., 2009 [25]	Spertus et al., 2009 [26]	Arnold et al., 2009 [27]	Leifheit-Limson et al., 2010 [28]	Shin & Choi, 2010 [29]	de Jong-Watt & Sherifi, 2011 [30]	Bucholz et al., 2011 [31]	Dueñas et al., 2011 [32]	Ginzburg & Ein-Dor, 2011 [33]	Panthee et al., 2011 [34]	Brink et al., 2012 [35]	Leifheit-Limson et al.,2012 [36]	Brink., 2012 [37]	Williams et al., 2012 [38]	Sertoz et al., 2013 [39]	Hosseini et al., 2014 [40]	Bennett et al., 2015 [41]	Salazar et al., 2016 [42]	Dzubur et al., 2016 [43]	Mahesh et al., 2017 [44]	Kang et al., 2018 [45]	Xia et al., 2019 [46]	Wulandari et al., 2020 [47]	Kang et al., 2021 [48]	Džubur et al., 2022 [49]	Rasmussen et al., 2022 [50]	Upadhyay et al., 2022 [51]	Ilassi et al., 2023 [52]	Sauletzhanovna et al., 2024 [53]	Füller et al., 2025 [54]	Malm et al., 2025 [55]
Sex (female)	↓	↓	↓		↓	↓	↓		↓				↓				↓	↓		↓			↓	↓	↓			↓							↓		↓		↓	↓		↓					↓		↓↑	↓					
Age (older)		↑				↓		↑		↑		↑	♂ ↑ ♀ ↓			↓	↓	↑	↑			↑	↓									↓			↓		↓↑		↓	↓		↓			↓										
Black vs whites															↓											↓																													
Non-Caucasian vs Caucasian								↑										↑											↓																										
Educational level (lower)																								↓															↓	↓	↓							↓						↓	
Occupational qualification (lower)																																																						↓	
Employment status (unemployed or sick leave)			↓			↓	↓									↓																																							

Financial status (lower)																					↓								↓	↓											↓							↓						↓
Insurance status (yes)																																									↑													
↑ reserves at the end of month																																									↑													
Social support (low)																												↓		↓						↓									↓						↓			
Marital/partner status (having a partner)			↑				↑																																			↑												
Marital satisfaction																																															↑							
Living alone			↓				↓																								↓																							
Number of children in charge																																																				↑		
Socioeconomic status (low)																																									↓													
Return to work																																↑																						
Previous exercise behavior			↑				↑																																			↑												
Smoking																↓																																				↓		
Alcohol or substance abuse																		↓																																				
Adherence to medications (low)										↓																																												
Depression	↓		↓	↓		↓	↓	↓	↓		↓		↓	↓		↓	↓						↓	↓					↓								↓		↓	↓	↓				↓	↓		↓	↓		↓		↓	
Anxiety	↓		↓	↓			↓									↓																														↓	↓						↓	

Fatigue						↓										↓	↓						
Disturbed sleep																↓							
ASD														↓									
PTSD														↓									
Sense of coherence											↑						↑						↑
↑ Perceived stress																			↓				
Coping strategies	↑↓				↑↓										↑								
Agency		↑																					
Unmitigated communion		↓																					
Level of masculinity (higher)			↑																				
Understanding of illness																						↑	
Optimism																	↑						
Type D personality																		↓					
Self-efficacy																↑							
Reserve capacity																			↑				
Negative mood	↓																						
Baseline QOL higher				↑			↑	↑	↑	↓											↑		
Mental health (worse)												↓	↓			↓				↓			
Physical health (lower)										↓		↓							↓				
Poor access to care										↓													

LVEF (lower)																↓						↓														↑					↓		↓							
Revascularization in the post discharge interim						↑				↓							↑													♂ ↑ ♀ ↓		↓																		
Length of stay longer	↓				↓																																													↑
Presence of cardiac symptoms			↓				↓																							↓																				
Prior CABG surgery				↓		↓																																↓												
Severity of infarction (higher)	↓			↓	↓																																													
Type of ACS (STEMI vs NSTEMI)																																										↑	↑							
Unstable angina vs MI						↓																								↓																				
Previous MI															↓	↓																																		
Time passed after MI																														↑										↑										
Rehospitalization after index hospitalization for MI								↓																						↓																				
Localization of the index MI other than anterior														↓																																				

Revascularization during initial hospitalization		↑							
History of PCI			↓						
Cardiac events during follow up period				↓					
Subsequent MI							↓		
pPCI vs medications									↑
CABG vs delayed PCI									↑
Acute thrombolytic therapy					↓			↓	
LVIDd									↑↓
Mitral regurtation									↑↓
Discharge prescription of an ACE inhibitor or an ARB					↓				
Acute mitral regurgitation, acute ventriculoseptal defect, or tamponade	↓								
Q wave MI								↓	
Dyspnea						↓			

Shock sustained in-hospital		↑													
Stress test not performed			↓												
Comorbidities	↓			↓	↓				↓	↓			↓		
Diabetes		↑	↓			↓								↓	↑
Previous stroke			↓	↓							↓			↓	
Heart failure			↓	↓				↓							
History of CHD							↑		↓			↓			
Hypertension										↓		↓			
Gastrointestina l problems					↓										
Rhematic disease					↓										
COPD			↓												
Chronic lung disease						↓									
Elevated serum creatinine			↓												
Anemia								↓							
History of arthritis			↓												
History of other heart disease														↓	
Hypercholester olemia											↓				
Creatine kinase level		↓													
Peptic ulcer disease			↓												

↓: negative association with Health-Related Quality of Life; ↑ positive association with Health-Related Quality of Life; ↓↑ some dimensions of Health-Related Quality of Life were better and some others were worse; ♀ women; ♂ men
ACE, Angiotensin-Converting Enzyme inhibitors; ACS, Acute Coronary Syndrome; ARB, Angiotensin Receptor Blockers; ASD, Acute stress disorder; CABG, Coronary Artery Bypass Graft; CHD, Coronary heart disease; COPD, Chronic obstructive pulmonary disease; LVEF, Left Ventricular Ejection Fraction; LVIDd, Left Ventricular Internal Dimension at End-Diastole; MI, Myocardial Infarction; NSTEMI, "non-ST" Elevation Myocardial Infarction; PCI, Percutaneous Coronary Intervention; pPCI, Primary Percutaneous Coronary Intervention; PTSD, Posttraumatic Stress Disorder; QOL, Quality of Life; STEMI, "ST" Elevation Myocardial Infarction.

References

1. Bogg, J.; Thornton, E.; Bundred, P. Gender Variability in Mood, Quality of Life and Coping Following Primary Myocardial Infarction. *Coron. Health Care* **2000**, *4*, 163–168, doi:10.1054/chec.2000.0095.
2. Fritz, H.L. Gender-Linked Personality Traits Predict Mental Health and Functional Status Following a First Coronary Event. *Health Psychol.* **2000**, *19,* 420-428, doi: 10.1037/0278-6133.19.5.420.
3. Lane, D.; Carroll, D.; Ring, C.; Beevers, D.G.; Lip, G.Y.H. Effects of Depression and Anxiety on Mortality and Quality-of-Life 4 Months after Myocardial Infarction. *J. of Psychosom. Res.* **2000**, *49*, 229–238, doi:10.1016/S0022-3999(00)00170-7.
4. Mayou, R.A.; Gill, D.; Thompson, D.R.; Day, A.; Hicks, N.; Volmink, J.; Neil, A. Depression and Anxiety As Predictors of Outcome After Myocardial Infarction: *Psychosom. Med.* **2000**, *62*, 212–219, doi:10.1097/00006842-200003000-00011.
5. Radley, A.; Grove, A.; Wright, S.; Thurston, H. Gender-Role Identity after Heart Attack: Links with Sex and Subjective Health Status. *Psychol. & Health* **2000**, *15*, 123–133, doi:10.1080/08870440008400293.
6. Beck, C.A.; Joseph, L.; Bélisle, P.; Pilote, L. Predictors of Quality of Life 6 Months and 1 Year after Acute Myocardial Infarction. *Am. Heart J.* **2001**, *142*, 271–279, doi:10.1067/mhj.2001.116758.
7. Lane, D.; Carroll, D.; Ring, C.; Beevers, D.G.; Lip, G.Y.H. Mortality and Quality of Life 12 Months After Myocardial Infarction: Effects of Depression and Anxiety: *Psychosom. Med.* **2001**, *63*, 221–230, doi:10.1097/00006842-200103000-00005.
8. Rumsfeld, J.S.; Magid, D.J.; Plomondon, M.E.; O'Brien, M.M.; Spertus, J.A.; Every, N.R.; Sales, A.E. Predictors of Quality of Life Following Acute Coronary Syndromes. *Am. J. Cardiol.* **2001**, *88*, 781–784, doi:10.1016/S0002-9149(01)01852-5.
9. Brink, E.; Karlson, B.W.; Hallberg, L.R.-M. Health Experiences of First-Time Myocardial Infarction: Factors Influencing Women's and Men's Health-Related Quality of Life after Five Months. *Psychol. Health & Med.* **2002**, *7*, 5–16, doi:10.1080/13548500120101522.

10. McBurney, C.R.; Eagle, K.A.; Kline-Rogers, E.M.; Cooper, J.V.; Mani, O.C.M.; Smith, D.E.; Erickson, S.R. Health-Related Quality of Life in Patients 7 Months After a Myocardial Infarction: Factors Affecting the Short Form-12. *Pharmacother. J. Hum. Pharmacol. Drug Ther.* **2002**, *22*, 1616–1622, doi:10.1592/phco.22.17.1616.34121.
11. Rumsfeld, J.S.; Magid, D.J.; Plomondon, M.E.; Sales, A.E.; Grunwald, G.K.; Every, N.R.; Spertus, J.A. History of Depression, Angina, and Quality of Life after Acute Coronary Syndromes. *Am. Heart J.* **2003**, *145*, 493–499, doi:10.1067/mhj.2003.177.
12. Bengtsson, I.; Hagman, M.; Währborg, P.; Wedel, H. Lasting Impact on Health-Related Quality of Life after a First Myocardial Infarction. *Int. J. Cardiol.* **2004**, *97*, 509–516, doi:10.1016/j.ijcard.2003.12.011.
13. Brink, E.; Grankvist, G.; Karlson, B.W.; Hallberg, L.R.-M. Health-Related Quality of Life in Women and Men One Year after Acute Myocardial Infarction. *Qual. Life Res.* **2005**, *14*, 749–757, doi:10.1007/s11136-004-0785-z.
14. Fauerbach, J.A.; Bush, D.E.; Thombs, B.D.; McCann, U.D.; Fogel, J.; Ziegelstein, R.C. Depression Following Acute Myocardial Infarction: A Prospective Relationship With Ongoing Health and Function. *Psychosomatics* **2005**, *46*, 355–361, doi:10.1176/appi.psy.46.4.355.
15. Spertus, J.; Safley, D.; Garg, M.; Jones, P.; Peterson, E.D. The Influence of Race on Health Status Outcomes One Year After an Acute Coronary Syndrome. *J. Am. Coll. of Cardiol.* **2005**, *46*, 1838–1844, doi:10.1016/j.jacc.2005.05.092.
16. Dickens, C.M.; McGowan, L.; Percival, C.; Tomenson, B.; Cotter, L.; Heagerty, A.; Creed, F.H. Contribution of Depression and Anxiety to Impaired Health-Related Quality of Life Following First Myocardial Infarction. *Br. J. Psychiatry* **2006**, *189*, 367–372, doi:10.1192/bjp.bp.105.018234.
17. De Jonge, P.; Spijkerman, T.A.; Van Den Brink, R.H.S.; Ormel, J. Depression after Myocardial Infarction Is a Risk Factor for Declining Health Related Quality of Life and Increased Disability and Cardiac Complaints at 12 Months. *Heart* **2006**, *92*, 32–39, doi:10.1136/hrt.2004.059451.
18. Peterson, P.N.; Spertus, J.A.; Magid, D.J.; Masoudi, F.A.; Reid, K.; Hamman, R.F.; Rumsfeld, J.S. The Impact of Diabetes on One-Year Health Status Outcomes Following Acute Coronary Syndromes. *BMC Cardiovasc. Disord.* **2006**, *6*, 41, doi:10.1186/1471-2261-6-41.
19. Failde, I.I.; Soto, M.M. Changes in Health Related Quality of Life 3 Months after an Acute Coronary Syndrome. *BMC Public Health* **2006**, *6*, 18, doi:10.1186/1471-2458-6-18.
20. Norris, C.M.; Hegadoren, K.; Pilote, L. Depression Symptoms Have a Greater Impact on the 1-Year Health-Related Quality of Life Outcomes of Women Post-Myocardial Infarction Compared to Men. *Eur. J. of Cardiovasc. Nurs.* **2007**, *6*, 92–98, doi:10.1016/j.ejcnurse.2006.05.003.
21. Rahimi, A.R.; Spertus, J.A.; Reid, K.J.; Bernheim, S.M.; Krumholz, H.M. Financial Barriers to Health Care and Outcomes After Acute Myocardial Infarction. *JAMA* **2007**, *297*, 1063, doi:10.1001/jama.297.10.1063.
22. Ho, P.M.; Eng, M.H.; Rumsfeld, J.S.; Spertus, J.A.; Peterson, P.N.; Jones, P.G.; Peterson, E.D.; Alexander, K.P.; Havranek, E.P.; Krumholz, H.M.; et al. The Influence of Age on Health Status Outcomes after Acute Myocardial Infarction. *Am. Heart J.* **2008**, *155*, 855–861, doi:10.1016/j.ahj.2007.11.032.
23. Thombs, B.D.; Ziegelstein, R.C.; Stewart, D.E.; Abbey, S.E.; Parakh, K.; Grace, S.L. Usefulness of Persistent Symptoms of Depression to Predict Physical Health Status 12 Months After an Acute Coronary Syndrome. *Am. J. Cardiol.* **2008**, *101*, 15–19, doi:10.1016/j.amjcard.2007.07.043.
24. Arnold, S.V.; Alexander, K.P.; Masoudi, F.A.; Ho, P.M.; Xiao, L.; Spertus, J.A. The Effect of Age on Functional and Mortality Outcomes After Acute Myocardial Infarction. *J. Am. Geriatr. Soc.* **2009**, *57*, 209–217, doi:10.1111/j.1532-5415.2008.02106.x.

25. Bergman, E.; Malm, D.; Karlsson, J.-E.; Berterö, C. Longitudinal Study of Patients after Myocardial Infarction: Sense of Coherence, Quality of Life, and Symptoms. *Heart & Lung* **2009**, *38*, 129–140, doi:10.1016/j.hrtlng.2008.05.007.
26. Spertus, J.A.; Jones, P.G.; Masoudi, F.A.; Rumsfeld, J.S.; Krumholz, H.M. Factors Associated With Racial Differences in Myocardial Infarction Outcomes. *Ann. Intern. Med.* **2009**, *150*, 314–324, doi:10.7326/0003-4819-150-5-200903030-00007.
27. Arnold, S.V.; Spertus, J.A.; Jones, P.G.; Xiao, L.; Cohen, D.J. The Impact of Dyspnea on Health-Related Quality of Life in Patients with Coronary Artery Disease: Results from the PREMIER Registry. *Am. Heart J.* **2009**, *157*, 1042-1049.e1, doi:10.1016/j.ahj.2009.03.021.
28. Leifheit-Limson, E.C.; Reid, K.J.; Kasl, S.V.; Lin, H.; Jones, P.G.; Buchanan, D.M.; Parashar, S.; Peterson, P.N.; Spertus, J.A.; Lichtman, J.H. The Role of Social Support in Health Status and Depressive Symptoms After Acute Myocardial Infarction: Evidence for a Stronger Relationship Among Women. *Circ: Cardiovasc. Qual. Outcomes* **2010**, *3*, 143–150, doi:10.1161/CIRCOUTCOMES.109.899815.
29. Shin, N.-M.; Choi, J. Relationship Between Survivors' Perceived Health Status Following Acute Coronary Syndrome and Depression Symptoms During Early Recovery Phase. *Asian Nurs. Res.* **2010**, *4*, 174–184, doi:10.1016/S1976-1317(11)60002-9.
30. de Jong-Watt, W.; Sherifi, I. Patient-Centred Assessment of Social Support, Health Status and Quality of Life in Patients with Acute Coronary Syndrome. *Can. J. Cardiovasc. Nurs.* **2011**, *21*, 26–33.
31. Bucholz, E.M.; Rathore, S.S.; Gosch, K.; Schoenfeld, A.; Jones, P.G.; Buchanan, D.M.; Spertus, J.A.; Krumholz, H.M. Effect of Living Alone on Patient Outcomes After Hospitalization for Acute Myocardial Infarction. *Am. J. Cardiol.* **2011**, *108*, 943–948, doi:10.1016/j.amjcard.2011.05.023.
32. Dueñas, M.; Ramirez, C.; Arana, R.; Failde, I. Gender Differences and Determinants of Health Related Quality of Life in Coronary Patients: A Follow-up Study. *BMC Cardiovasc. Disord.* **2011**, *11*, 24, doi:10.1186/1471-2261-11-24.
33. Ginzburg, K.; Ein-Dor, T. Posttraumatic Stress Syndromes and Health-Related Quality of Life Following Myocardial Infarction: 8-Year Follow-Up. *Gen. Hosp. Psychiatry* **2011**, *33*, 565–571, doi:10.1016/j.genhosppsych.2011.08.015.
34. Panthee, B.; Kritpracha, C.; Chinnawong, T. Correlation between Coping Strategies and Quality of Life among Myocardial Infarction Patients in Nepal. *Nurse Media J. Nurs.* **2011**, *1*, 187–194.
35. Brink, E.; Alsén, P.; Herlitz, J.; Kjellgren, K.; Cliffordson, C. General Self-Efficacy and Health-Related Quality of Life after Myocardial Infarction. *Psychol. Health Med.* **2012**, *17*, 346–355, doi:10.1080/13548506.2011.608807.
36. Leifheit-Limson, E.C.; Reid, K.J.; Kasl, S.V.; Lin, H.; Buchanan, D.M.; Jones, P.G.; Peterson, P.N.; Parashar, S.; Spertus, J.A.; Lichtman, J.H. Changes in Social Support within the Early Recovery Period and Outcomes after Acute Myocardial Infarction. *J. Psychosom. Res.* **2012**, *73*, 35–41, doi:10.1016/j.jpsychores.2012.04.006.
37. Brink, E. Considering Both Health-Promoting and Illness-Related Factors in Assessment of Health-Related Quality of Life After Myocardial Infarction. *Open Nurs. J.* **2012**, *6*, 90-94.
38. Williams, L.; O'Connor, R.C.; Grubb, N.R.; O'Carroll, R.E. Type D Personality and Three-Month Psychosocial Outcomes among Patients Post-Myocardial Infarction. *J. Psychosom. Res.* **2012**, *72*, 422–426, doi:10.1016/j.jpsychores.2012.02.007.
39. Sertoz, O.O.; Aydemir, O.; Gulpek, D.; Elbi, H.; Ozenli, Y.; Yilmaz, A.; Ozan, E.; Atesci, F.; Abay, E.; Semiz, M.; et al. The Impact of Physical and Psychological Comorbid Conditions on the Quality of Life of Patients with Acute Myocardial Infarction: A Multi-Center, Cross-Sectional Observational Study from Turkey. *Int. J. Psychiatry Med.* **2013**, *45*, 97–109, doi:10.2190/PM.45.2.a.

40. Hosseini, S.H.; Ghaemian, A.; Mehdizadeh, E.; Ashraf, H. Contribution of Depression and Anxiety to Impaired Quality of Life in Survivors of Myocardial Infarction. *Int. J. Psychiatry Clin. Pract.* **2014**, *18*, 175–181, doi:10.3109/13651501.2014.940049.
41. Bennett, K.K.; Buchanan, D.M.; Jones, P.G.; Spertus, J.A. Socioeconomic Status, Cognitive-Emotional Factors, and Health Status Following Myocardial Infarction: Testing the Reserve Capacity Model. *J. Behav. Med.* **2015**, *38*, 110–121, doi:10.1007/s10865-014-9583-4.
42. Salazar, A.; Dueñas, M.; Fernandez-Palacin, F.; Failde, I. Factors Related to the Evolution of Health Related Quality of Life in Coronary Patients. A Longitudinal Approach Using Weighted Generalized Estimating Equations with Missing Data. *Int. J. Cardiol.* **2016**, *223*, 940–946, doi:10.1016/j.ijcard.2016.08.300.
43. Dzubur, A.; Mekic, M.; Pesto, S.; Nabil, N. Echocardiographic Parameters as Life Quality Predictors in Patients After Myocardial Infarction Treated with Different Methods. *Med. Arh.* **2016**, *70*, 419, doi:10.5455/medarh.2016.70.419-424.
44. Mahesh, P.K.B.; Gunathunga, M.W.; Jayasinghe, S.; Arnold, S.M.; Haniffa, R.; De Silva, A.P. Pre-Event Quality of Life and Its Influence on the Post-Event Quality of Life among Patients with ST Elevation and Non-ST Elevation Myocardial Infarctions of a Premier Province of Sri Lanka. *Health Qual. Life Outcomes* **2017**, *15*, 154, doi:10.1186/s12955-017-0730-9.
45. Kang, K.; Gholizadeh, L.; Han, H.-R.; Inglis, S.C. Predictors of Health-Related Quality of Life in Korean Patients with Myocardial Infarction: A Longitudinal Observational Study. *Heart Lung* **2018**, *47*, 142–148, doi:10.1016/j.hrtlng.2017.12.005.
46. Xia, K.; Wang, L.-F.; Yang, X.-C.; Jiang, H.-Y.; Zhang, L.-J.; Yao, D.-K.; Hu, D.-Y.; Ding, R.-J. Comparing the Effects of Depression, Anxiety, and Comorbidity on Quality-of-Life, Adverse Outcomes, and Medical Expenditure in Chinese Patients with Acute Coronary Syndrome. *Chin. Med. J.* **2019**, *132*, 1045–1052, doi:10.1097/CM9.0000000000000215.
47. Wulandari, D.; Ginanjar, A.S.; Purwono, U.; Purba, D. Marital Satisfaction, Anxiety, and Health-Related Quality of Life in Myocardial Infarction Patients. *J. Glob. Pharma Technol.* **2020**, *12*, 483-495.
48. Kang, K.; Gholizadeh, L.; Han, H.-R. Health-Related Quality of Life and Its Predictors in Korean Patients with Myocardial Infarction in the Acute Phase. *Clin. Nurs. Res.* **2021**, *30*, 161–170, doi:10.1177/1054773819894692.
49. Džubur, A.; Lisica, D.; Hodžić, E.; Begić, E.; Lepara, O.; Fajkić, A.; Gogić, E.; Ejubović, M. Relationship between Depression and Quality of Life after Myocardial Infarction. *Med. Glas.* **2022**, *19*, 0–0, doi:10.17392/1404-21.
50. Rasmussen, A.A.; Fridlund, B.; Nielsen, K.; Rasmussen, T.B.; Thrysoee, L.; Borregaard, B.; Thorup, C.B.; Berg, S.K.; Mols, R.E. Gender Differences in Patient-Reported Outcomes in Patients with Acute Myocardial Infarction. *Eur. J. Cardiovasc. Nurs.* **2022**, *21*, 772–781, doi:10.1093/eurjcn/zvac022.
51. Upadhyay, V.; Bhandari, S.S.; Rai, D.P.; Dutta, S.; García-Grau, P.; Vaddiparti, K. Improving Depression and Perceived Social Support Enhances Overall Quality of Life among Myocardial Infarction Survivors: Necessity for Integrating Mental Health Care into Cardiac Rehabilitation Programs. *Egypt J. Neurol. Psychiatry Neurosurg.* **2022**, *58*, 87, doi:10.1186/s41983-022-00521-6.
52. Jlassi, O.; Omrane, A.; Ben Massoud, M.; Khalfallah, T.; Bouzgarrou, L.; Gamra, H. Determinants of Health-Related Quality of Life among Patients with Ischemic Heart Disease. *Health Syst.* **2024**, *13*, 322–331, doi:10.1080/20476965.2023.2275799.
53. Sauletzhanovna, T.A.; Mohammed, W.K.; Ahmed, A.S.; Mohammed, H.I.; Al-Hili, A.; Alnajar, M.J.; Naser, N.S.; Amr, E.F.; Mohsin, R.M. The Predictive Value of Depression and Anxiety on Protracted Cardiovascular Outcomes in Individuals with Acute Myocardial Infarction. *Int. J. Body Mind Cult.* **2024**, 64–75, doi:10.22122/ijbmc.v11isp.739.

54. Füller, D.; Andresen-Bundus, H.; Pagonas, N.; Jaehn, P.; Ukena, C.; Gödde, K.; Holmberg, C.; Ritter, O.; Sasko, B. Adverse Socioeconomic Factors Are Associated with a Widening Gap in One-Year Health-Related Quality of Life after Acute Myocardial Infarction. *Sci. Rep.* **2025**, *15*, 19791, doi:10.1038/s41598-025-04604-1.
55. Malm, D.; Mårtensson, J.; Årestedt, K. Sense of Coherence and Quality of Life in the Recovery of Women and Men with Myocardial Infarction: A 10-Year Follow-up Study. *Eur. J. Cardiovasc. Nurs.* **2025**, *24*, 631–639, doi:10.1093/eurjcn/zvaf028.
