# Supplementary material for: Determinants of Health-Related Quality of Life After Acute Coronary Syndromes: A Systematic Review
Source: Healthcare (Basel). 2026 May 9;14(10):1292. doi: 10.3390/healthcare14101292 (PMC13205943; doi:10.3390/healthcare14101292)
Supplement: Supplementary file 1 [file healthcare-14-01292-s001.zip › Supplementary Table S4.pdf]

Supplementary Table S4. Determinants of health-related quality of life after acute coronary syndromes.

Reference	Determinants	Outcomes	Measures of effect	P-value
Bogg et al., 2000 [1]	• Female sex	• ↓ emotional QOL	• NR	• <0.01
	• Emotion focused coping	• ↑ social QOL	• b=0.49	• <0.001
	• Depression at baseline	• ↓ social QOL	• b=0.92	• <0.001
	• Depression at one month	• ↓ social QOL	• b=0.76	• <0.001
	• Depression at baseline	• ↓ physical QOL	• b=0.52	• <0.001
	• Depression at one month	• ↓ physical QOL	• b=0.89	• <0.001
	• Absence of anxiety at baseline	• ↑ emotional QOL	• b=0.89	• <0.001
	• Absence of anxiety at one month	• ↑ emotional QOL	• b=0.76	• <0.001
	• Negative mood at one month	• ↓ emotional QOL	• b=0.69	• <0.001
	• Negative mood at one month	• ↓ physical QOL	• b=0.46	• <0.001
	• Emotion focused coping	• ↑ emotional QOL	• b=0.67	• <0.001
	• Not using avoidance coping	• ↑ emotional QOL	• b=0.52	• <0.001
	• Task oriented coping	• ↑ social QOL	• b=0.63	• <0.001
Fritz, 2000 [2]	• Female sex	• ↓ physical functioning	• NR	• < 0.001
	• Older age	• ↑ mental functioning	• r = 0.31	• < 0.05
	• Agency	• ↑ physical functioning	• r= 0.28	• < 0.05
	• Unmitigated communion	• ↓ mental functioning	• r= -0.29	• < 0.05
	• Agency	• ↑ mental functioning	• b=0.3	• <0.05
Lane et al., 2000 [3]	• Male sex	• ↑ HRQOL	• r=0.31	• <0.05
	• Having a partner	• ↑ HRQOL	• b=0.22	• 0.002
	• Not living alone	• ↑ HRQOL	• r=0.2	• <0.05
	• Being employed	• ↑ HRQOL	• r=0.18	• <0.05
	• Higher Peel Index score	• ↓ HRQOL	• b= 0.24	• 0.001
	• Longer hospital stay	• ↓ HRQOL	• r=0.15	• <0.04
	• Higher frequency of previous exercise behavior	• ↑ HRQOL	• r=-0.21	• < 0.003
	• Higher duration of previous exercise behavior	• ↑ HRQOL	• r=-0.17	• 0.02
	• High anxiety scores	• ↓ HRQOL	• b= 0.18	• 0.02

	• Depression	• ↓ HRQOL	• b=0.2	• 0.0001
Mayou et al., 2000 [4]	• Combined anxiety and depression (distress)	• ↓ HRQOL	• NR	• <0.05
Radley et al., 2000 [5]	• Higher levels of masculinity<br>• Female sex<br>• More cardiac symptoms<br>• More comorbid illnesses	• ↑ health status<br>• ↓ health status<br>• ↓QOL<br>• ↓QOL	• b=0.32<br>• NR<br>• b=-0.40<br>• b=-0.16	• < 0.05<br>• < 0.05<br>• < 0.05<br>• < 0.05
Beck et al., 2001 [6]	• ↑ QOL score at baseline<br>• ↑ BDI score<br>• Older age<br>• Previous bypass surgery<br>• Shock sustained in-hospital<br>• Creatine kinase level<br>• Being on sick leave at baseline<br>• Killip class I<br>• ↑ QOL scores at baseline<br>• Older age<br>• ↑ BDI score at baseline<br>• Previous bypass surgery<br>• Male sex<br>• At least one episode of acute mitral regurgitation, acute ventriculoseptal defect, or tamponade<br>• Diabetes	• ↑ PCS, MCS, and EuroQol scores at 6 months<br>• ↓ PCS, MCS, and EuroQol scores at 6 months<br>• ↓ PCS and EuroQol scores at 6 months<br>• ↓ PCS scores at 6 months<br>• ↑PCS scores at 6 months<br>• ↓ MCS scores at 6 months<br>• ↓ EuroQol scores at 6 months<br>• ↑ PCS scores at 6 months<br>• ↑ PCS, MCS, and EuroQol scores at 1 year<br>• ↓ PCS and EuroQol scores at 1 year<br>• ↓ MCS scores at 1 year<br>• ↓ in both PCS and EuroQol scores at 1 year<br>• ↑ PCS at 1 year<br>• ↓ EuroQol at 1 year<br>• ↑EuroQol at 1 year	• b= 3.6, 2.4, 2.0<br>• b= –1.6, –3.7, –5.4<br>• b= –1.5, –2.7<br>• b= –5.6<br>• b=9.9<br>• b=–0.1<br>• b= –23.8<br>• b=4.1<br>• b=4.6, 3.3, 2.2<br>• b= –1.3, –2.5<br>• b= –3<br>• b=–5.3, –12.5<br>• b= 3.6<br>• b= –34.3<br>• b= 6.7	• < 0.05<br>• < 0.05<br>• < 0.05<br>• < 0.05<br>• < 0.05<br>• < 0.05<br>• < 0.05<br>• < 0.05<br>• < 0.05<br>• < 0.05<br>• < 0.05<br>• < 0.05<br>• < 0.05<br>• < 0.05<br>• < 0.05
Lane et al., 2001 [7]	• Female sex<br>• Having a partner	• ↓ QOL<br>• ↑ QOL	• r=0.2<br>• r=0.22	• <0.05<br>• <0.05

	• Not living alone<br>• Being employed<br>• Higher Peel Index score<br>• Worse Killip class designation<br>• Longer hospital stay<br>• Higher frequency of previous exercise behavior<br>• High anxiety scores<br>• Depression	• ↑ QOL<br>• ↑ QOL<br>• ↓ QOL<br>• ↓ QOL<br>• ↓ QOL<br>• ↑ QOL<br>• ↓ QOL<br>• ↓ QOL	• b= 3.79<br>• r=0.18<br>• b= 0.34<br>• r=0.15<br>• r=0.25<br>• r=-0.18<br>• b= 0.10<br>• b=0.21	• 0.001<br>• <0.05<br>• 0.001<br>• <0,05<br>• <0.001<br>• 0.02<br>• 0.008<br>• 0.001
Rumsfeld et al., 2001 [8]	• History of arthritis<br>• Chronic obstructive pulmonary disease<br>• Prior CABG surgery<br>• Congestive heart failure<br>• Stroke<br>• Diabetes:<br>• Peptic ulcer disease<br>• Depression<br>• Elevated serum creatinine<br>• No stress test during the index ACS hospitalization<br>• Discharge diagnosis of unstable angina (as opposed to myocardial infarction)<br>• Revascularization with either PCI or CABG surgery during the initial hospitalization<br>• Revascularization with either PCI or CABG surgery during the post-discharge period<br>• Non-Caucasian race<br>• History of depression<br>• Stroke<br>• Revascularization with either PCI or CABG surgery during the index hospitalization<br>• Older age	• ↓ physical health status<br>• ↓ physical health status<br>• ↓ physical health status<br>• ↓ physical health status<br>• ↓ physical health status<br>• ↓ physical health status<br>• ↓ physical health status<br>• ↓ physical health status<br>• ↓ physical health status<br>• ↓ physical health status<br>• ↓ physical health status<br>• ↑ physical health status<br>• ↑ physical health status<br>• ↑ physical health status<br>• ↓ mental health status<br>• ↓ mental health status<br>• ↑ mental health status<br>• ↑ mental health status	• b= -4.40<br>• b= -3.67<br>• b=-3.46<br>• b= -3.42<br>• b= -3.08<br>• b=-2.39<br>• b=-2.24<br>• b=-1.71<br>• b=-0.73<br>• b=-1.79<br>• b= -1.33<br>• b=3.18<br>• b= 2.04<br>• b=2.03<br>• b=-12.03<br>• b=-2.40<br>• b=1.41<br>• b=0.08	• 0.001<br>• 0.001<br>• 0.001<br>• 0.001<br>• 0.001<br>• 0.001<br>• 0.007<br>• 0.003<br>• 0.002<br>• 0.002<br>• 0.012<br>• 0.001<br>• 0.003<br>• 0.02<br>• 0.001<br>• 0.008<br>• 0.040<br>• 0.002
Brink et al.,	• Female sex	• ↓ PCS	• NR	• <0.05

2002 [9]	• Depression<br>• Coping strategy fatalism<br>• Health complaints/symptoms<br>• Coping strategy minimization<br>• Depression<br>• Health complaints/symptoms	• ↓ PCS<br>• ↓ PCS<br>• ↓ PCS<br>• ↑MCS<br>• ↓MCS<br>• ↓MCS	• b= -0.34<br>• b= -0.25<br>• b=-0.52<br>• b=0.27<br>• b=-0.38<br>• b=-0.3	• < 0.01<br>• <0.05<br>• < 0.001<br>• <0.01<br>• <0,01<br>• <0.001
McBurney et al., 2002 [10]	• Younger than 65 years:<br>• Lower compliance with overall drug therapy<br>• Low compliance with lipid-lowering therapy<br>• More comorbid diseases<br>• History of PCI<br>• Chronic heart failure<br>• Transient Ischemic Attack<br>• Rehospitalization in the period after the index hospitalization	• ↓MCS<br>• ↓MCS<br>• ↓MCS<br>• ↓ PCS<br>• ↓ PCS<br>• ↓ PCS<br>• ↓ PCS<br>• ↓ PCS	• b=0.22<br>• b=0.24<br>• b=0.20<br>• b=-0.24<br>• b=-0.15<br>• b=-0.16<br>• b=-0.14<br>• b=-0.24	• 0.001<br>• 0.001<br>• 0.003<br>• 0.001<br>• 0.02<br>• 0.02<br>• 0.04<br>• <0.001
Rumsfeld et al., 2003 [11]	• History of depression	• ↓ QOL	• OR=2.84	• 0.001
Bengtsson et al., 2004 [12]	• Age group <59 years<br>• Age group <59 years<br>• Age group <59 years<br>• No invasive intervention during follow-up	• ↓ Emotional Functioning<br>• ↓ Social Functioning in the Cardiac Health Profile<br>• ↓ Social Functioning in the SF-36<br>• ↑ MCS scores	• NR<br>• NR<br>• NR<br>• NR	• 0.03<br>• 0.05<br>• 0.03<br>• <0.001
Brink et al., 2005 [13]	• Female sex<br><br><u>Predictors (female sex)</u><br>• Depression measured one week after AMI<br>• Depression at five months after AMI<br>• Depression at five months after AMI<br>• Older age<br>• Fatigue five months after AMI<br><br><u>Predictors (male sex)</u><br>• Depression five months after AMI	• ↓ PCS one year after AMI<br><br><br>• ↓ PCS one year after AMI<br>• ↓ PCS one year after AMI<br>• ↓ MCS one year after AMI<br>• ↓ PCS one year after AMI<br>• ↓ MCS one year after AMI<br><br><br>• ↓ PCS one year after MI	• NR<br><br><br>• b=-0.27<br>• b= -0.35<br>• b=-0.57<br>• b=-0.28<br>• b=- 0.25<br><br><br>• b=-0.13	• <0.01<br><br><br>• <0.01<br>• <0.01<br>• <0.01<br>• <0.01<br>• <0.01<br><br><br>• <0.01

	• Fatigue five months after AMI<br>• Depression one week after AMI<br>• Older age<br>• Depression five months after AMI<br>• Fatigue five months after AMI	• ↓ PCS one year after AMI<br>• ↓ MCS one year after AMI<br>• ↑ MCS one year after AMI<br>• ↓ MCS 1 year after AMI<br>• ↓ MCS 1 year after AMI	• b=-0.63<br>• b=-0.29<br>• b=0.27<br>• b=-0.09<br>• b=-0.24	• <0.01<br>• <0.01<br>• <0.05<br>• <0.01<br>• <0.01
Fauerbach et al., 2005 [14]	• At baseline, depressed group<br>• At baseline, depressed group<br>• At follow-up: depression<br><br>• Depressed patients at the baseline	• ↓ SF-36 mental domain<br>• ↓ SF-36 physical domain<br>• ↓ in the mental domain<br><br>• ↓ global health at 4 months<br>• ↓ mental health at 4 months<br>• ↓ role interference by emotional problems at 4 months<br>• ↓ vitality at 4 months<br>• ↓ social functioning at 4 months	• NR<br>• NR<br>• NR<br><br>• NR<br>• NR<br>• NR<br>• NR<br>• NR	• <0.01<br>• 0.04<br>• <0.01<br><br>• < 0.01<br>• < 0.01<br>• < 0.01<br>• < 0.01<br>• < 0.01
Spertus et al., 2005 [15]	• Race: Blacks vs whites	• ↓ QOL (SAQ scores)<br>• ↓ SF-12 PCS	• NR<br>• NR	• 0.002<br>• 0.005
Dickens et al., 2006 [16]	• Older age<br>• ↑ physical QOL at baseline<br><br>• Gastrointestinal problems<br>• Rheumatic disease<br>• Presence of other medical problems<br>• Current or past smokers<br>• Infarction site categorized as "other" (as opposed to "anterior")<br>• Experience of further cardiac events (like another hospital admission for a cardiac problem) during the 12-month follow-up	• ↓ QOL<br>• ↑ physical QOL at one year<br><br>• ↓ physical QOL<br>• ↓ physical QOL<br>• ↓ physical QOL<br>• ↓ physical QOL<br>• ↓ physical QOL<br>• ↓ physical QOL	• b=-21<br>• b=0.18<br><br>• b=-0.12<br>• b=-0.15<br>• b=-0.13<br>• b=-0.15<br>• b=-0.1<br>• b=-0.12	• 0.001<br>• 0.005<br><br>• 0.021<br>• <0.008<br>• 0.018<br>• 0.006<br>• 0.04<br>• 0.028

	period			
	Higher levels of depression at 6-months	↓ physical QOL at one year	b=-0.21	0.004
	Higher levels of anxiety at 6 months	↓ physical QOL at one year	b=-0.15	0.037
	Depression at 12 months	↓ PCS scores at one year	NR	<0.0005
	Being employed at the time of AMI vs being on sick leave	↑ mean PCS at baseline	NR	<0.0005
	Those who had returned to work at 12-months vs those who had not	↑ mean PCS at follow-up	NR	<0.003
de Jonge et al., 2006 [17]	Health status at 3 months	↑ physical functioning at one year	b=0.70	<0.05
	History of MI	↓ physical functioning at one year	b=-6.32	<0.05
	Post MI depression	↓ physical functioning at one year	b=-6.73	<0.05
	Health status at 3 months	↑ social functioning at one year	b= 0.47	<0.05
	Older age at MI	↓ social functioning at one year	b= -0.17	<0.05
	Female sex	↓ social functioning at one year	b=-5.30	<0.05
	Post MI depression	↓ social functioning at one year	b=-10.36	<0.05
	Health status at 3 months	↑ role limitations-physical at one year	b=0.44	<0.05
	Female sex	↓ role limitations-physical at one year	b=-12.26	<0.05
	Post MI depression	↓ role limitations-physical at one year	b=-15.46	<0.05
	Health status at 3 months	↑ role limitations-emotional at one year	b=0.37	<0.05
	History of MI	↓ role limitations-emotional at one year	b== -11.71	<0.05
	Post MI depression:	↓ role limitations-emotional at one year	b=-20.44	<0.05

	Health status at 3 months	↑ general health at one year	b= 0.57	<0.05
	Post MI depression	↓ general health at one year	b=-6.43	<0.05
	Health status at 3 months	↑ scores in pain domain (less pain) at one year	b=0.52	<0.05
	Post MI depression	↓ scores in pain domain (more pain) at one year	b=-5.74	<0.05
Peterson et al., 2006 [18]	Diabetes	↓ SAQ physical limitation<br>↓ SAQ-HRQOL<br>↓ PCS	OR=1.94<br>OR=1.43<br>b = 2.85	<0.05<br><0.05<br>0.01
	Female sex	↓ SAQ physical limitation	OR=1.65	<0.05
	Caucasian race	↓ SAQ physical limitation	OR=0.61	<0.05
	History of MI	↓ SAQ physical limitation	OR=1.61	<0.05
	History of alcohol or substance abuse	↓ SAQ physical limitation	OR=2.28	<0.05
	Discharge prescription of an angiotensin converting enzyme inhibitor or an ARBs	↓ SAQ physical limitation	OR=1.52	<0.05
	Chronic lung disease	↓ SAQ physical limitation	OR=2.15	<0.05
	Age	↓ SAQ-HRQoL	OR=1.28	<0.05
	Ejection fraction	↓ SAQ-HRQoL	OR=1.12	<0.05
	Acute thrombolytic therapy	↓ SAQ-HRQoL	OR=1.92	<0.05
	Chronic lung disease	↓ SAQ-HRQoL	OR=2.36	<0.05
Failde & Soto, 2006 [19]	Revascularization performed during the follow-up	The decrease in PCS at 3 months (from baseline) was 6.4 points less in patients submitted to a revascularization	b= -6.497	0.047
	Age	The decrease in PCS at 3 months (from baseline) was 0.2 points less for each year of age	b=-0.259	0.037
	Interaction between previous history of coronary heart disease and the presence of one or more risk factors:	A reduced decrease (4.7 points) at 3 months (from baseline) was found	b=-4.783	0.080

Norris et al., 2007 [20]	• Female sex<br>• Female sex<br>• Female sex<br>• Male sex	• ↓ PCS at baseline and 1 year<br>• ↓ MCS at baseline<br>• ↓ MCS at 1 year<br>• Higher mean adjusted PCS scores	• NR<br>• NR<br>• NR<br>• NR	• <0.01<br>• <0.01<br>• 0.01<br>• 0.008
Rahimi et al., 2007 [21]	• Financial barriers to health care services<br>• Financial barriers to Medication	• ↓ SAQ QOL, ↓ SF-12 PCS, ↓ SF-12 MCS<br>• ↓ SAQ QOL, ↓ SF-12 PCS, ↓ SF-12 MCS	• NR<br>• NR	• <0.05<br>• <0.05
Ho et al., 2008 [22]	• Older age at baseline<br>• Older age at 12 months	• ↑ baseline HRQOL<br>• ↑ one-year HRQOL	• NR<br>• NR	• < 0.0001<br>• < 0.0001
Thombs et al., 2008 [23]	<u>Independent effect of in-hospital symptoms of depression on physical health status 12 months after ACS</u><br>• ↑SF-12 PCS before ACS<br>• Older age<br>• Female sex<br>• Higher in-hospital BDI score<br><u>Independent effects of persistent symptoms of depression on physical health status 12 months after ACS</u><br>• ↑SF-12 PCS before ACS<br>• Older age<br>• Female sex<br>• Persistent Symptoms of depression	• ↑ physical health status 12 months after ACS<br>• ↓ physical health status 12 months after ACS<br>• ↓ physical health status 12 months after ACS<br>• ↓ physical health status 12 months after ACS<br>• ↑ physical health status 12 months after ACS<br>• ↓ physical health status 12 months after ACS<br>• ↓ physical health status 12 months after ACS<br>• ↓ physical health status 12	• b=0.459<br>• b=-0.190<br>• b=-0.096<br>• b=-0.150<br>• b=0.451<br>• b= -0.189<br>• b=-0.084<br>• b= -0.220	• < 0.001<br>• <0.001<br>• 0.018<br>• <0.001<br>• < 0.001<br>• <0.001<br>• 0.037<br>• <0.001

		months after ACS		
Arnold et al., 2009 [24]	• Female sex<br>• Low education (high school)<br>• Having difficulty getting medical care<br>• Congestive heart failure<br>• Anemia on admission<br>• Depressive symptoms<br>• Moderate or severe left ventricular systolic dysfunction during index hospitalization (ejection fraction< 40%)<br>• Higher baseline SF-12 score<br>• Decline in physical function	• ↓ QOL<br>• ↓ QOL<br>• ↓ QOL<br>• ↓QOL<br>• ↓ QOL<br>• ↓QOL<br>• ↓QOL<br>• ↓ physical function<br>• ↓ SAQ QOL scores	• OR = 1.37<br>• OR= 1.34<br>• OR = 1.65<br>• OR = 1.96<br>• OR = 1.60<br>• OR = 1.97<br>• OR = 1.63<br>• OR = 1.09<br>• NR	• 0.01<br>• 0.01<br>• 0.003<br>• 0.001<br>• 0.001<br>• < 0.001<br>• 0.001<br>• <0.001<br>• <0.001
Bergman et al., 2009 [25]	• Male sex<br>• Male sex<br>• High Sense of Coherence<br>• High Sense of Coherence	• ↑ PCS-12 and MCS-12 at discharge and 2 weeks after discharge<br>• ↑ SAQ QOL<br>• ↑ SAQ QOL<br>• ↑ MCS-12 at discharge	• NR<br>• OR = 1.05<br>• OR = 1.04<br>• OR=1.07	• <0.05<br>• <0.001<br>• 0.015<br>• 0.04
Spertus et al., 2009 [26]	Race: Blacks vs whites	• ↓ one-year QOL	• NR	• < 0.001
Arnold et al., 2009 [27]	• 1 month dyspnea score<br>• Change in dyspnea score (from 1 month to 1 year): increase in dyspnea severity	• ↓ SF-12 PCS<br>• ↓ SAQ QOL<br>• ↓ SF-12 PCS<br>• ↓ SAQ QOL	• b= −2.47<br>• b= −2.43<br>• b = −3.81<br>• b=−3.49	• <0.001<br>• < 0.001<br>• <0.001<br>• <0.001
Leifheit-Limson et al., 2010 [28]	• Lower Social Support<br>• Lower Social Support	• ↓ SAQ QoL<br>• ↓ mental functioning	• b= -3.33<br>• b=-1.72	• <0.05<br>• <0.05

	• Lower Social Support	• ↓ PCS	• NR	• <0.001
	• Female sex	• ↓ QOL, particularly among the low social support group.	• b = -4.89	• 0.015
Shin & Choi, 2010 [29]	<u>Time 1: Baseline</u>			
	• Higher scores in mental health	• ↑ perceived health status	• r=0 .85	• <0.05
	• Higher scores in physical health	• ↑ perceived health status	• r= 0.88	• <0.05
	• Higher scores in physical health	• ↑ perceived mental health	• r=0.56	• <0.05
	• Higher scores in BDI	• ↓ perceived health status	• r= −0.63	• <0.05
	• Higher scores in BDI	• ↓perceived mental health	• r= −0.70	• <0.05
	• Higher scores in BDI	• ↓perceived physical health	• r= −0.44	• <0.05
	<u>Time 2: one-month post discharge</u>			
	• Higher scores in mental health	• ↑ perceived health status	• r= 0.83	• <0.05
	• Higher scores in physical health	• ↑ perceived health status	• r=0.92	• <0.05
	• Higher scores in physical health	• ↑ perceived mental health	• r=0.58	• <0.05
	• Higher scores in BDI	• ↓ perceived health status	• r= −0.62	• <0.05
	• Higher scores in BDI	• ↓ perceived mental health	• r=−0.76	• <0.05
	• Higher scores in BDI	• ↓ perceived physical health	• r= −0.42	• <0.05
	• Annual household income ≤$40,000:	• ↓ perceived health status at Time 1 and at Time 2	• NR	• <0.05
	• African Americans VS Caucasian patients:	• ↓ perceived health status at Time 2	• NR	• <0.05
	• History of depression:	• ↓ perceived health status at Time 1 and Time 2	• NR<br>• NR	• <0.05<br>• <0.05
	• Prescription of antidepressants:	• ↓ perceived health status at Time 1 and Time 2	• NR	• <0.05
de Jong-Watt & Sherifi, 2011 [30]	• Higher perceived social support	• ↑ treatment satisfaction and Cardiac-HRQOL	• NR	• < 0.05
	• Higher annual income	• ↑ treatment satisfaction and	• NR	• < 0.05

		Cardiac-HRQOL		
Bucholz et al., 2011 [31]	• Living alone	• ↓ SF-12 PCS (baseline)	• NR	• <0.001
	• Living alone	• ↓ Mean QOL (one year)	• NR	• 0.034
Dueñas et al., 2011 [32]	♂ Predictors of QOL			
	• Older age	• ↓ PCS	• B =-0.25	• 0.000
	• Previous history of CHD	• ↓ PCS	• B =-3.29	• 0.013
	• Rehospitalization (Between 3 and 6 months)	• ↓ PCS	• B =-9.79	• 0.013
	• Higher scores in GHQ-28 (Mental health	• ↓ PCS	• B = -1.46	• 0.000
	• 6 months after AMI	• ↑ PCS	• B = 3.44	• 0.016
	• Comorbidity	• ↓ Vitality	• B =-9.53	• 0.001
	• Higher scores in GHQ-28	• ↓ Vitality	• B =-3.75	• 0.000
	• Older age	• ↓ Social Functioning	• B=-0.54	• 0.002
	• Frequency of angina (≥ 1 between baseline-3 months)	• ↓ Social Functioning	• B = -22.94	• 0.019
	• Frequency of angina (≥ 1 between 3 and 6 months)	• ↓ Social Functioning	• B =-21.62	• 0.006
	• Higher scores in GHQ-28	• ↓ Social Functioning	• B =-2.87	• 0.000
	• 6 months after AMI	• ↑ Social Functioning	• B = 8.1	• 0.003
	• Higher scores in GHQ-28	• ↓ Role emotional	• B=-2.20	• 0.006
	• 3 months after AMI	• ↑ Role emotional	• B =10.90	• 0.000
	• Revascularization (Between baseline-3 months)	• ↑ Mental Health	• B = 20.91	• 0.011
	• Frequency of angina (≥1 between baseline-3 months	• ↓ Mental Health	• B =-12.68	• 0.01
	• Return to work 4 (Between baseline-3 months	• ↑ Mental Health	• B = 7.48	• 0.043
	• Higher scores in GHQ-28	• ↓ Mental Health	• B= -1.83	• 0.000
	♀ Predictors of QOL			
	• Previous history of CHD	• ↓ PCS	• B=-8.32	• 0.000
	• Diagnostic group (AMI)	• ↑ PCS	• B= 4.52	• 0.033
	• Frequency of angina (≥1 between 3 and 6	• ↓ PCS	• B=-10.58	• 0.000

	months)	↓ PCS		
	• Higher scores in GHQ-28	• ↑ PCS	• B = -0.57	• 0.000
	• 6 months after AMI	• ↓ MCS	• B = 8.8	• 0.000
	• Revascularization (Between 3 and 6 months)	• ↓ MCS	• B=-25.75	• 0.000
	• Frequency of angina (≥1 between 3 and 6 months)		• B=18.21	• 0.001
		• ↓ MCS		
	• Higher scores in GHQ-28		• B=-1.34	• 0.000
Ginzburg & Ein-Dor, 2011 [33]	• Chronic group vs Recovered/resilient group	• ↓ Physical HRQoL	• NR	• <0.001
	• Chronic group vs Recovered/resilient group	• ↓ Mental HRQoL	• NR	• <0.01
	• Severity of ASD at initial hospitalization	• ↓ physical health	• r=−0.27	• <0.01
		• ↓ mental health	• r=−0.30	• <0.01
	• Severity of PTSD at 7 months after MI	• ↓ physical health	• r=−0.38,	• <0.001
		• ↓ mental health	• r=−0.41	• <0.001
	• Severity of PTSD at 8 years after MI	• ↓ physical health	• r=−0.44	• <0.001
	• Severity of PTSD at 8 years after MI	• ↓ mental health	• r=−0.49	• <0.001
	• In the chronic group, the greater the initial probability of having ASD:	• the lower the physical HRQOL 8 years after MI	• b=−16.57	• <0.001
		• the lower the mental HRQOL 8 years after MI.	• b=−21.31	• <0.001
Panthee et al., 2011 [34]	• History of re-infarction	• ↓ QoL	• NR	• <0.05
	• Patients who needed revascularization	• ↓ QoL	• NR	• <0.05
	• Problem-focused coping	• ↑ QoL	• r = 0.41	• <0.01
	• Problem-focused coping	• ↑ health and functioning dimension	• r= 0.39	• <0.01
	• Problem-focused coping	• ↑ socio-economic dimension	• r= 0.46	• <0.01
Brink et al., 2012 [35]	• Female sex	• ↓ PCS	• NR	• 0.015
	• Older age	• ↓ PCS	• r=-0.229	• < 0.01
	• Fatigue	• ↓ PCS	• r=-0.602	• < 0.001
	• Disturbed sleep	• ↓ PCS	• r=-0.384	• < 0.001
	• Increased levels of GSE	• ↑ PCS	• r=0.177	• < 0.05
	• Fatigue	• ↓MCS	• r=-0.678	• < 0.001
	• Disturbed sleep	• ↓MCS	• r=-0.383	• < 0.001
	• Increased levels of GSE	• ↑MCS	• r=0.246	• < 0.01
	• Better MCS	• ↑PCS	• r=0.283	• < 0.01

Leifheit-Limson et al.,2012 [36]	<u>Baseline social support entered separately</u>			
	• Low baseline support	• ↓ SAQ QOL	• b=–2.65	• 0.02
		• ↓ SF-12 MCS	• b =–2.57	• <0.001
	<u>1-Month social support entered separately</u>			
	• Low 1-month support	• ↓ SAQ QOL	• b=–7.41	• <0.001
		• ↓ SF-12 MCS	• b =–4.97	• <0.001
	<u>Baseline and 1-month social support entered simultaneously</u>			
	• Low1-month support	• ↓ SAQ QOL	• b=–7.19	• <0.001
		• ↓ SF-12MCS	• b=–4.54	• <0.001
	• Low baseline support	• ↓ SF-12MCS	• b=–1.35	• 0.02
Brink., 2012 [37]	• Fatigue	• ↓ PCS	• b=-0.430	• <0.001
	• Older age	• ↓ PCS	• b=-0.297	• <0.001
	• Females	• ↓ PCS	• b=0.183	• 0.026
	• Depression	• ↓ MCS	• b=-0.337	• 0.001
	• Fatigue	• ↓ MCS	• b=-.0337	• 0.001
	• Older age	• ↑ MCS	• b= 0.159	• 0.042
	• Sense of coherence	• ↑ PCS	• r= 0.315	• <0.01
	• Sense of coherence	• ↑ MCS	• r= 0.315	• <0.01
	• Optimism	• ↑ PCS	• r= 0.249	• <0.05
	• Optimism	• ↑ MCS	• r= 0.486	• <0.001
Williams et al., 2012 [38]	• Impaired LVF	• ↓ QOL	• b=–0.165	• <0.05
	• Negative Affect (characteristic of Type D Personality)	• ↓ QOL	• b=–0.624	• <0.01
	• Type D personality	• ↓ QOL	• NR	• <0.001
Sertoz et al., 2013 [39]	• Older age	• ↓ physical QOL	• B =–0.149	• 0.002
	• Female sex	• ↓ physical QOL	• B =–3.314	• 0.006
	• Low education level	• ↓ physical QOL	• B = 0.405	• < 0.0001
	• Hypertension	• ↓ physical QOL	• B = 4.835	• < 0.0001
	• Higher BDI score	• ↓ physical QOL	• B =–0.993	• <0.0001
	• Female sex	• ↓ Psychological QOL	• B =–2.723	• 0.013
	• Low education level	• ↓ Psychological QOL	• B = 0.436	• < 0.0001
	• Higher BDI score	• ↓ Psychological QOL	• B =–1.114	• < 0.0001
	• Older age	• ↓ Social relations QOL	• B=–0.248	• < 0.0001

	Low education level	↓ Social relations QOL	B = 0.436	0.002
	Hypertension	↓ Social relations QOL	B = 3.232	0.030
	Comorbid medical conditions	↓ Social relations QOL	B =–3.738	0.018
	Higher BDI score	↓ Social relations QOL	B =–1.170	< 0.0001
	Low education level	↓ environmental QOL	B = 0.681	< 0.0001
	Higher BDI score	↓ environmental QOL	B =–0.667	< 0.0001
Hosseini et al., 2014 [40]	Older Age	↓ PCS	b= -0.247	0.001
	Low Education level	↓ PCS	b=0.180	0.019
	CABG history	↓ PCS	b=-0.213	0.002
	CVA history	↓ PCS	b=-0.250	0.000
	Q-wave MI	↓ PCS	b=0.165	0.019
	Thrombolysis	↓ PCS	b=-0.174	0.011
	BDI score≥ 10	↓ PCS	b= -0.151	0.026
	Females sex	↓ MCS	B= -0.171	0.011
	Hypercholesterolemia	↓ MCS	B=-0.226	0.001
	CVA history	↓ MCS	B=-0.306	0.000
	BDI score≥10	↓ MCS	B=-0.249	0.000
Bennett et al., 2015 [41]	Low Income	↓ SAQ-QoL	r=0.10	<0.05
	Low Income	↓ SF-12 PCS	r=0.35	<0.05
	Low Income	↓SF-12 MCS	r=0.11	<0.05
	Low Education	↓ SF-12 PCS	r=0.20	<0.05
	Insurance status (yes) at baseline	↑SAQ-QoL	r=0.26	<0.05
	Insurance status (yes) at baseline	↑SF-12PCS	r=0.11	<0.05
	Insurance status (yes) at baseline	↑SF-12MCS	r=0.25	<0.05
	↑ Reserves at the end of the month	↑SAQ-QoL	r=0.22	<0.05
	↑ Reserves at the end of the month	↑SF-12PCS	r=0.28	<0.05
	↑ Reserves at the end of the month	↑SF-12MCS	r=0.22	<0.05
	↑ Median household income in residential zip code at baseline	↑ SF-12PCS	r=0.18	<0.05
	↓ Social Support at 1 month	↓SAQ-QoL	r=0.14	<0.05
	↓ Social Support at 1 month	↓SF-12PCS	r=0.09	<0.05
	↓ Social Support at 1 month	↓SF-12MCS	r= 0.30	<0.05
	↑ Life Orientation Test-Revised at 1-month	↑SAQ-QoL	r=0.22	<0.05
	↑ Life Orientation Test-Revised at 1-month	↑SF-12PCS	r=0.18	<0.05

	↑ Life Orientation Test-Revised at 1-month	↑SF-12MCS	r= 0.28	<0.05
	Health locus of control at 1 month	↑SF-12PCS	r=0.12	<0.05
	Health locus of control at 1 month	↑SF-12MCS	r=0.05	<0.05
	↑ Perceived Stress Scale-4 at 1-month	↓SAQ-QoL	r= -0.34	<0.05
	↑ Perceived Stress Scale-4 at 1-month	↓SF-12PCS	r=-0.25	<0.05
	↑ Perceived Stress Scale-4 at 1-month	↓SF-12 MCS	r=-0.42	<0.05
	Depressive symptoms at 6 months	↓SAQ-QoL	r=-0.42	<0.05
	Depressive symptoms at 6 months	↓SF-12PCS	r=-0.37	<0.05
	Depressive symptoms at 6 months	↓SF-12MCS	r=-0.52	<0.05
	↑SAQ-QoL	↑SF-12PCS	r=0.35	<0.05
	↑SAQ-QoL	↑SF-12MCS	r= 0.45	<0.05
	↑SF-12PCS	↑SF-12MCS	r= 0.05	<0.05
	Low baseline Socioeconomic status	↓ SF-12 PCS scores	b = 0.17	<0.001
	Low Socioeconomic status	↓ SF-12MCS	b=0.08	<0.01
	Reserve capacity (variable comprised of perceived social support, optimism, and health locus of control) at 1 month	↑ SF-12 MCS scores	b = 0.22	<0.05
	Negative emotions at 6 months	↓ SF-12 MCS	b =-0.22	<0.05
	↓ Socioeconomic status	↓ SAQ-QoL	b= 0.07	<0.10
Salazar et al., 2016 [42]	Older Age	↓ Pain	B= −0.5	<0.001
	Single/Separated	↓ General health	B= −14.5	<0.05
	Widow(er)	↓ Physical functioning	B=−23.2	<0.05
	Performing heart-healthy physical activity	↑ Physical functioning	B= 13.9	<0.001
	Previous history CHD	↓ PCS	B=−5.2	<0.01
	Higher scores in Depression (GHQ-28)	↓PCS	B= −1.2	<0.001
	Hypertension	↓ Role Physical	B=−19.8	<0.01
	Time 6 months	↑ Role Physical	B=13.3	<0.05
	Female sex	↓ MCS	B= −7.0	< 0.05
	Older age	↓ Vitality	B= −0.3	< 0.05
	Single/Separated	↓ Social Functioning	B= −14.1	< 0.05
	Single/Separated	↓Mental Health	B= −23.3	< 0.05
	Widow(er)	↓ Social Functioning	B= −29.8	<0.001
	Hypertension	↓ Vitality	B= −8.9	< 0.01
	Higher scores in (GHQ-28)	↓ Vitality	B= −3.8	<0.001

	• Higher scores in (GHQ-28	• ↓ Social Functioning	• B= −2.6,	• <0.001
Dzubur et al., 2016 [43]	• Treated with medications<br>• PCI immediately after the incident<br>• Patients who underwent Delayed PCI<br>• Patients who underwent CABG<br><br>Patients treated with medications<br>• LVEF<br>Patients treated with PCI immediately after the incident<br>• ↑ LVIDd<br>• ↑ LVIDd<br>• LVEF<br><br><br><br><br>• Mitral regurgitation:<br><br>Patients treated with delayed PCI<br>• ↑ LVIDd<br>• LVEF:<br>• Mitral regurtation:<br><br>Patients treated with CABG<br>• Mitral regurtation	• Lowest scores in physical role<br>• Highest scores in physical role<br>• Lowest scores in social functioning<br>• Highest scores in social functioning<br><br><br>• ↑ QOL<br><br>• ↓ physical functioning<br>• ↓ general health<br>• ↑ vitality<br>• ↑ mental health<br>• ↑pain<br>• ↑general health<br>• ↑ vitality<br><br>• ↑ pain<br>• ↑ emotional role<br>• ↓ the physical functioning<br>• ↓ physical role<br>• ↓ social functioning<br><br>• ↓ emotional role<br>• ↓ mental health	• NR<br>• NR<br>• NR<br>• NR<br><br><br>• NR<br><br>• r=-0.31<br>• r=-0.35<br>• NR<br>• NR<br>• NR<br>• NR<br>• NR<br><br>• r=0.35<br>• NR<br>• NR<br>• NR<br>• NR<br><br>• NR<br>• NR	• 0.017<br>• 0.017<br>• 0.016<br>• 0.016<br><br><br>• 0.00<br><br>• 0.045<br>• 0.026<br>• 0.02<br>• 0.00<br>• 0.00<br>• 0.02<br>• 0.04<br><br>• 0.02<br>• 0.00<br>• 0.00<br>• 0.00<br>• 0.01<br><br>• 0.03<br>• 0.02
Mahesh et al., 2017 [44]	• Patients with STEMI vs patients with NSTEMI	• ↑ pre-event QOL<br>○ ↑ General health	 • NR	 • 0.003

		○ ↑ Physical- functioning<br>○ ↑ Pain<br>○ ↑ Role-limitation-physical<br>○ ↑ Role-limitation-emotional<br>○ ↑ Social functioning<br>○ ↑ Emotional well-being	• NR<br>• NR<br>• NR<br>• NR<br>• NR<br>• NR	• 0.012<br>• 0.004<br>• 0.002<br>• 0.006<br>• 0.028<br>• 0.007
	• Patients with STEMI vs patients with NSTEMI	• ↑ post discharge general health	• NR	• 0.042
	• STEMI patients with higher pre-event QOL	• ↑ discharge QOL<br>○ General Health<br>○ Physical-Functioning<br>○ Vitality<br>○ Social-Functioning<br>○ Emotional Well-Being	 • b=0.625<br>• b=0.335<br>• b=0.465<br>• b= 0.297<br>• b= 0.566	 • <0.001<br>• 0.002<br>• <0.001<br>• 0.020<br>• <0.001
	• NSTEMI patients with higher pre-event QOL	• ↑ post-discharge QOL in all domains<br>○ General Health<br>○ Physical-Functioning<br>○ Pain<br>○ Role Limitation-physical<br>○ Role Limitation-emotional<br>○ Vitality<br>○ Social-Functioning<br>○ Emotional Well-Being	 • b=0.673<br>• b=0.494<br>• b=0.358<br>• b=0.203<br>• b=0.333<br>• b=0.490<br>• b=0.445<br>• b= 0.536	 • <0.001<br>• <0.001<br>• <0.001<br>• <0.001<br>• <0.001<br>• <0.001<br>• <0.001<br>• <0.001
Kang et al., 2018 [45]	• Younger age<br>• Diagnosed with STEMI<br>• Higher LVEF at baseline<br>• Patients’ better understanding of their illness<br>• Perceived higher social support at baseline<br>Higher score of depression at baseline	• ↑ HRQOL at three months<br>• ↑ HRQOL at three months<br>• ↑ HRQOL at three months<br>• ↑ HRQOL at three months<br>• ↑ HRQOL at three months<br>• ↓ HRQOL at three months	• b=-0.216<br>• b=-0.163<br>• b=0.207<br>• b=0.213<br>• b=0.199<br>• b=-0.201	• 0.008<br>• 0.036<br>• 0.010<br>• 0.007<br>• 0.013<br>• 0.009
Xia et al., 2019 [46]	• Baseline comorbidity of depression and anxiety	• ↓ 12-month SF-12 QOL<br>• ↓ 12-month PCS	• OR=1.77<br>• OR=1.62	• 0.003<br>• 0.01

		• ↓ 12-month MCS	• OR= 1.75	• 0.004
	• Patients with baseline comorbidity of depression and anxiety at baseline VS patients with only depression, only anxiety and normal group	• The lowest SF-12 QOL	• NR	• <0.0001
	• Patients with only depression (follow up)	• The lowest SF-12 QOL	• NR	• 0.04
	• Patients with baseline comorbidity of depression and anxiety (follow up)	• The lowest MCS	• NR	• 0.004
Kang et al., 2021 [47]	• Higher Education<br>• Better perceived financial status<br>• Diabetes<br>• History of Stroke<br>• History of other heart disease<br>• Higher score of the DASS 21	• ↑ HRQOL<br>• ↑ HRQOL<br>• ↓ HRQOL<br>• ↓ HRQOL<br>• ↓ HRQOL<br>• ↓ HRQOL	• b=0.228<br>• b=−0.169<br>• b=−0.210<br>• b=−0.150<br>• b=−0.193<br>• b=−0.442	• 0.000<br>• 0.008<br>• 0.001<br>• 0.015<br>• 0.002<br>• 0.000
Wulandari et al., 2020 [48]	• Female sex<br>• Higher cardiac anxiety<br>• Higher marital satisfaction	• ↓ HRQOL<br>• ↓ HRQOL<br>• ↑ HRQOL	• b=0.190<br>• b=-0.553<br>• b=0.271	• < 0.05<br>• 0.00<br>• 0.00
Džubur et al., 2022 [49]	• Depression	• ↓ Physical functioning<br>• ↓ Physical role<br>• ↓ Pain<br>• ↓ General health<br>• ↓ Vitality<br>• ↓ Emotional role<br>• ↓ Social function<br>• ↓ Mental health	• r = -0.701<br>• r= -0.538<br>• r= -0.477<br>• r= -0.510<br>• r= -0.453<br>• r= -0.212<br>• r= -0.359<br>• r=-0.218	• <0.01<br>• <0.01<br>• <0.01<br>• <0.01<br>• <0.01<br>• <0.01<br>• <0.01<br>• <0.01
	• >65 years ♀<br>• >65 years ♀<br>• <65 years ♀<br>• <65 years ♀	• ↓ physical functioning<br>• ↓ general health<br>• ↓ emotional role<br>• ↑ general health	• NR<br>• NR<br>• NR<br>• NR	• 0.019<br>• 0.001<br>• 0.014<br>• 0.028
Rasmussen et al., 2022 [50]	• Female sex	• ↓ SF-12 PCS<br>• ↓ SF-12 MCS<br>• ↓ Heart-QoL global<br>• ↓ EQ-5D-5L<br>• ↓ EQ VAS	• b=-1.34<br>• b=-1.36<br>• b=-0.11<br>• b=-0.03<br>• b=- 3.22	• <0.05<br>• <0.05<br>• <0.05<br>• <0.05<br>• <0.05

Upadhyay et al., 2022 [51]	• ↑ Perceived Social Support<br>• ↑ Cardiac Depression	• ↑ total QoL<br>• ↓ QoL Total	• b=0.05<br>• b = − 0.0256	• < 0.001<br>• <0.001
Jlassi et al., 2023 [52]	• Hospital stay<br>• Type 2 diabetes<br>• Smoking<br>• Number of children in charge<br>• LVEF	• ↑ Global QOL<br>• ↑ Global QOL<br>• ↓ Global QOL<br>• ↑ Global QOL<br>• ↑ Social QOL	• b=0.440<br>• b=0.396<br>• b=–0.424<br>• b=0.312<br>• b=0.337	• 0.004<br>• 0.008<br>• 0.008<br>• 0.046<br>• 0.014
Sauletzhanovna et al., 2024 [53]	• ↑ HADS-Anxiety score<br>• ↑ HADS-Depression score<br>• ↑ PHQ-9 score<br>• ↑ HADS-Anxiety score<br>• ↑ HADS-Depression score<br>• ↑ PHQ-9 score	• ↓ PCS<br>• ↓ PCS<br>• ↓ PCS<br>• ↓ MCS<br>• ↓ MCS<br>• ↓ MCS	• b= -0.82<br>• b=-0.78<br>• b=-0.75<br>• b=-1.24<br>• b=-1.18<br>• b=-1.15	• <0.001<br>• < 0.001<br>• < 0.001<br>• < 0.001<br>• < 0.001<br>• < 0.001
Füller et al., 2025 [54]	• Lower education level<br><br>• Poor financial situation<br>• Lower occupational qualification	• ↓ HRQoL at baseline<br>• ↓ HRQoL at follow-up<br>• ↓ HRQoL at follow-up<br>• ↓ HRQoL at follow-up	• NR<br>• NR<br>• NR<br>• NR	• 0.005<br>• 0.01<br>• 0.05<br>• 0.03
Malm et al., 2025 [55]	• Higher SOC-13 scores	• ↑ mental health<br>• ↑ physical health<br>• ↑ treatment satisfaction<br>• ↑ disease perception domain	• B=0.51<br>• B= 0.41<br>• B=0.79<br>• B=0.66	• < 0.001<br>• < 0.05<br>• < 0.01<br>• <0.10

♀: female sex; ♂ male sex; ↓: negative association with Health-Related Quality Of Life; ↑: positive association with Health-Related Quality Of Life.

ACS, Acute Coronary Syndrome; AMI, Acute Myocardial Infarction; ARB, Angiotensin Receptor Blockers; ASD, Acute Stress Disorder; BDI, Beck Depression Inventory; CABG, Coronary Artery Bypass Graft; CHD, Coronary Heart Disease; CVA, Cerebrovascular Accident; DASS 21, Depression Anxiety Stress Scales-21 items; EQ VAS, EuroQol Visual Analogue Scale; EQ-5D-5L, EuroQol 5-Dimensions 5-Levels; EuroQol, European Quality of Life ; GHQ-28, General Health Questionnaire-28; GSE, General Self-Efficacy; HADS, Hospital Anxiety and Depression Scale; HRQOL, Health-Related Quality Of Life; LVEF, Left Ventricular Ejection Fraction; LVF, Left Ventricular Function; LVIDd, Left Ventricular Internal Diameter end-diastole; MCS, Mental Component Summary; MI, Myocardial Infarction; NR, Not Reported; NSTEMI, "non-ST" Elevation Myocardial Infarction; OR, Odds Ratio; PCI, Percutaneous Coronary Intervention; PCS, Physical Component Summary; PHQ-9, Patient Health

Questionnaire-9; PTSD, Post-Traumatic Stress Disorder; QOL, Quality Of Life; SAQ, Seattle Angina Questionnaire; SF-12, Short Form-12 Questionnaire; SF-36, Short Form-36 Questionnaire; STEMI, "ST" Elevation Myocardial Infarction.

References

1. Bogg, J.; Thornton, E.; Bundred, P. Gender Variability in Mood, Quality of Life and Coping Following Primary Myocardial Infarction. *Coron. Health Care* **2000**, *4*, 163–168, doi:10.1054/chec.2000.0095.
2. Fritz, H.L. Gender-Linked Personality Traits Predict Mental Health and Functional Status Following a First Coronary Event. *Health Psychol.* **2000**, *19*, 420-428, doi: 10.1037/0278-6133.19.5.420.
3. Lane, D.; Carroll, D.; Ring, C.; Beevers, D.G.; Lip, G.Y.H. Effects of Depression and Anxiety on Mortality and Quality-of-Life 4 Months after Myocardial Infarction. *J. Psychosom. Res.* **2000**, *49*, 229–238, doi:10.1016/S0022-3999(00)00170-7.
4. Mayou, R.A.; Gill, D.; Thompson, D.R.; Day, A.; Hicks, N.; Volmink, J.; Neil, A. Depression and Anxiety As Predictors of Outcome After Myocardial Infarction: *Psychosom. Med.* **2000**, *62*, 212–219, doi:10.1097/00006842-200003000-00011.
5. Radley, A.; Grove, A.; Wright, S.; Thurston, H. Gender-Role Identity after Heart Attack: Links with Sex and Subjective Health Status. *Psychol. Health* **2000**, *15*, 123–133, doi:10.1080/08870440008400293.
6. Beck, C.A.; Joseph, L.; Bélisle, P.; Pilote, L. Predictors of Quality of Life 6 Months and 1 Year after Acute Myocardial Infarction. *Am. Heart J.* **2001**, *142*, 271–279, doi:10.1067/mhj.2001.116758.
7. Lane, D.; Carroll, D.; Ring, C.; Beevers, D.G.; Lip, G.Y.H. Mortality and Quality of Life 12 Months After Myocardial Infarction: Effects of Depression and Anxiety: *Psychosom. Med.* **2001**, *63*, 221–230, doi:10.1097/00006842-200103000-00005.
8. Rumsfeld, J.S.; Magid, D.J.; Plomondon, M.E.; O'Brien, M.M.; Spertus, J.A.; Every, N.R.; Sales, A.E. Predictors of Quality of Life Following Acute Coronary Syndromes. *Am. J. Cardiol.* **2001**, *88*, 781–784, doi:10.1016/S0002-9149(01)01852-5.
9. Brink, E.; Karlson, B.W.; Hallberg, L.R.-M. Health Experiences of First-Time Myocardial Infarction: Factors Influencing Women's and Men's Health-Related Quality of Life after Five Months. *Psychol. Health Med.* **2002**, *7*, 5–16, doi:10.1080/13548500120101522.
10. McBurney, C.R.; Eagle, K.A.; Kline-Rogers, E.M.; Cooper, J.V.; Mani, O.C.M.; Smith, D.E.; Erickson, S.R. Health-Related Quality of Life in Patients 7 Months After a Myocardial Infarction: Factors Affecting the Short Form-12. *Pharmacother. J. Hum. Pharmacol. Drug Ther.* **2002**, *22*, 1616–1622, doi:10.1592/phco.22.17.1616.34121.
11. Rumsfeld, J.S.; Magid, D.J.; Plomondon, M.E.; Sales, A.E.; Grunwald, G.K.; Every, N.R.; Spertus, J.A. History of Depression, Angina, and Quality of Life after Acute Coronary Syndromes. *Am. Heart J.* **2003**, *145*, 493–499, doi:10.1067/mhj.2003.177.
12. Bengtsson, I.; Hagman, M.; Währborg, P.; Wedel, H. Lasting Impact on Health-Related Quality of Life after a First Myocardial Infarction. *Int. J. Cardiol.* **2004**, *97*, 509–516, doi:10.1016/j.ijcard.2003.12.011.
13. Brink, E.; Grankvist, G.; Karlson, B.W.; Hallberg, L.R.-M. Health-Related Quality of Life in Women and Men One Year after Acute Myocardial Infarction. *Qual. Life Res.* **2005**, *14*, 749–757, doi:10.1007/s11136-004-0785-z.
14. Fauerbach, J.A.; Bush, D.E.; Thombs, B.D.; McCann, U.D.; Fogel, J.; Ziegelstein, R.C. Depression Following Acute Myocardial Infarction: A Prospective Relationship With Ongoing Health and Function. *Psychosomatics* **2005**, *46*, 355–361, doi:10.1176/appi.psy.46.4.355.
15. Spertus, J.; Safley, D.; Garg, M.; Jones, P.; Peterson, E.D. The Influence of Race on Health Status Outcomes One Year After an Acute Coronary Syndrome. *J. Am. Coll. Cardiol.* **2005**, *46*, 1838–1844, doi:10.1016/j.jacc.2005.05.092.

16. Dickens, C.M.; McGowan, L.; Percival, C.; Tomenson, B.; Cotter, L.; Heagerty, A.; Creed, F.H. Contribution of Depression and Anxiety to Impaired Health-Related Quality of Life Following First Myocardial Infarction. *Br. J. Psychiatry* **2006**, *189*, 367–372, doi:10.1192/bjp.bp.105.018234.
17. De Jonge, P.; Spijkerman, T.A.; Van Den Brink, R.H.S.; Ormel, J. Depression after Myocardial Infarction Is a Risk Factor for Declining Health Related Quality of Life and Increased Disability and Cardiac Complaints at 12 Months. *Heart* **2006**, *92*, 32–39, doi:10.1136/hrt.2004.059451.
18. Peterson, P.N.; Spertus, J.A.; Magid, D.J.; Masoudi, F.A.; Reid, K.; Hamman, R.F.; Rumsfeld, J.S. The Impact of Diabetes on One-Year Health Status Outcomes Following Acute Coronary Syndromes. *BMC Cardiovasc. Disord.* **2006**, *6*, 41, doi:10.1186/1471-2261-6-41.
19. Failde, I.I.; Soto, M.M. Changes in Health Related Quality of Life 3 Months after an Acute Coronary Syndrome. *BMC Public Health* **2006**, *6*, 18, doi:10.1186/1471-2458-6-18.
20. Norris, C.M.; Hegadoren, K.; Pilote, L. Depression Symptoms Have a Greater Impact on the 1-Year Health-Related Quality of Life Outcomes of Women Post-Myocardial Infarction Compared to Men. *Eur. J. Cardiovasc. Nurs.* **2007**, *6*, 92–98, doi:10.1016/j.ejcnurse.2006.05.003.
21. Rahimi, A.R.; Spertus, J.A.; Reid, K.J.; Bernheim, S.M.; Krumholz, H.M. Financial Barriers to Health Care and Outcomes After Acute Myocardial Infarction. *JAMA* **2007**, *297*, 1063, doi:10.1001/jama.297.10.1063.
22. Ho, P.M.; Eng, M.H.; Rumsfeld, J.S.; Spertus, J.A.; Peterson, P.N.; Jones, P.G.; Peterson, E.D.; Alexander, K.P.; Havranek, E.P.; Krumholz, H.M.; et al. The Influence of Age on Health Status Outcomes after Acute Myocardial Infarction. *Am. Heart J.* **2008**, *155*, 855–861, doi:10.1016/j.ahj.2007.11.032.
23. Thombs, B.D.; Ziegelstein, R.C.; Stewart, D.E.; Abbey, S.E.; Parakh, K.; Grace, S.L. Usefulness of Persistent Symptoms of Depression to Predict Physical Health Status 12 Months After an Acute Coronary Syndrome. *Am. J. Cardiol.* **2008**, *101*, 15–19, doi:10.1016/j.amjcard.2007.07.043.
24. Arnold, S.V.; Alexander, K.P.; Masoudi, F.A.; Ho, P.M.; Xiao, L.; Spertus, J.A. The Effect of Age on Functional and Mortality Outcomes After Acute Myocardial Infarction. *J. Am. Geriatr. Soc.* **2009**, *57*, 209–217, doi:10.1111/j.1532-5415.2008.02106.x.
25. Bergman, E.; Malm, D.; Karlsson, J.-E.; Berterö, C. Longitudinal Study of Patients after Myocardial Infarction: Sense of Coherence, Quality of Life, and Symptoms. *Heart Lung* **2009**, *38*, 129–140, doi:10.1016/j.hrtlng.2008.05.007.
26. Spertus, J.A.; Jones, P.G.; Masoudi, F.A.; Rumsfeld, J.S.; Krumholz, H.M. Factors Associated With Racial Differences in Myocardial Infarction Outcomes. *Ann. Intern. Med.* **2009**, *150*, 314–324, doi:10.7326/0003-4819-150-5-200903030-00007.
27. Arnold, S.V.; Spertus, J.A.; Jones, P.G.; Xiao, L.; Cohen, D.J. The Impact of Dyspnea on Health-Related Quality of Life in Patients with Coronary Artery Disease: Results from the PREMIER Registry. *Am. Heart J.* **2009**, *157*, 1042-1049.e1, doi:10.1016/j.ahj.2009.03.021.
28. Leifheit-Limson, E.C.; Reid, K.J.; Kasl, S.V.; Lin, H.; Jones, P.G.; Buchanan, D.M.; Parashar, S.; Peterson, P.N.; Spertus, J.A.; Lichtman, J.H. The Role of Social Support in Health Status and Depressive Symptoms After Acute Myocardial Infarction: Evidence for a Stronger Relationship Among Women. *Circ. Cardiovasc. Qual. Outcomes* **2010**, *3*, 143–150, doi:10.1161/CIRCOUTCOMES.109.899815.
29. Shin, N.-M.; Choi, J. Relationship Between Survivors' Perceived Health Status Following Acute Coronary Syndrome and Depression Symptoms During Early Recovery Phase. *Asian Nurs. Res.* **2010**, *4*, 174–184, doi:10.1016/S1976-1317(11)60002-9.
30. de Jong-Watt, W.; Sherifi, I. Patient-Centred Assessment of Social Support, Health Status and Quality of Life in Patients with Acute Coronary Syndrome. *Can. J. Cardiovasc. Nurs.* **2011**, *21*, 26–33.

31. Bucholz, E.M.; Rathore, S.S.; Gosch, K.; Schoenfeld, A.; Jones, P.G.; Buchanan, D.M.; Spertus, J.A.; Krumholz, H.M. Effect of Living Alone on Patient Outcomes After Hospitalization for Acute Myocardial Infarction. *Am. J. Cardiol.* **2011**, *108*, 943–948, doi:10.1016/j.amjcard.2011.05.023.
32. Dueñas, M.; Ramirez, C.; Arana, R.; Failde, I. Gender Differences and Determinants of Health Related Quality of Life in Coronary Patients: A Follow-up Study. *BMC Cardiovasc. Disord.* **2011**, *11*, 24, doi:10.1186/1471-2261-11-24.
33. Ginzburg, K.; Ein-Dor, T. Posttraumatic Stress Syndromes and Health-Related Quality of Life Following Myocardial Infarction: 8-Year Follow-Up. *Gen. Hosp. Psychiatry* **2011**, *33*, 565–571, doi:10.1016/j.genhosppsych.2011.08.015.
34. Panthee, B.; Kritpracha, C.; Chinnawong, T. Correlation between Coping Strategies and Quality of Life among Myocardial Infarction Patients in Nepal. *Nurse Media J. Nurs.* **2011**, *1*, 187–194.
35. Brink, E.; Alsén, P.; Herlitz, J.; Kjellgren, K.; Cliffordson, C. General Self-Efficacy and Health-Related Quality of Life after Myocardial Infarction. *Psychol. Health Med.* **2012**, *17*, 346–355, doi:10.1080/13548506.2011.608807.
36. Leifheit-Limson, E.C.; Reid, K.J.; Kasl, S.V.; Lin, H.; Buchanan, D.M.; Jones, P.G.; Peterson, P.N.; Parashar, S.; Spertus, J.A.; Lichtman, J.H. Changes in Social Support within the Early Recovery Period and Outcomes after Acute Myocardial Infarction. *J. Psychosom. Res.* **2012**, *73*, 35–41, doi:10.1016/j.jpsychores.2012.04.006.
37. Brink, E. Considering Both Health-Promoting and Illness-Related Factors in Assessment of Health-Related Quality of Life After Myocardial Infarction. *Open Nurs. J.* **2012**, *6*, 90-94.
38. Williams, L.; O'Connor, R.C.; Grubb, N.R.; O'Carroll, R.E. Type D Personality and Three-Month Psychosocial Outcomes among Patients Post-Myocardial Infarction. *J. Psychosom. Res.* **2012**, *72*, 422–426, doi:10.1016/j.jpsychores.2012.02.007.
39. Sertoz, O.O.; Aydemir, O.; Gulpek, D.; Elbi, H.; Ozenli, Y.; Yilmaz, A.; Ozan, E.; Atesci, F.; Abay, E.; Semiz, M.; et al. The Impact of Physical and Psychological Comorbid Conditions on the Quality of Life of Patients with Acute Myocardial Infarction: A Multi-Center, Cross-Sectional Observational Study from Turkey. *Int. J. Psychiatry Med.* **2013**, *45*, 97–109, doi:10.2190/PM.45.2.a.
40. Hosseini, S.H.; Ghaemian, A.; Mehdizadeh, E.; Ashraf, H. Contribution of Depression and Anxiety to Impaired Quality of Life in Survivors of Myocardial Infarction. *Int. J. Psychiatry Clin. Pract.* **2014**, *18*, 175–181, doi:10.3109/13651501.2014.940049.
41. Bennett, K.K.; Buchanan, D.M.; Jones, P.G.; Spertus, J.A. Socioeconomic Status, Cognitive-Emotional Factors, and Health Status Following Myocardial Infarction: Testing the Reserve Capacity Model. *J. Behav. Med.* **2015**, *38*, 110–121, doi:10.1007/s10865-014-9583-4.
42. Salazar, A.; Dueñas, M.; Fernandez-Palacin, F.; Failde, I. Factors Related to the Evolution of Health Related Quality of Life in Coronary Patients. A Longitudinal Approach Using Weighted Generalized Estimating Equations with Missing Data. *Int. J. Cardiol.* **2016**, *223*, 940–946, doi:10.1016/j.ijcard.2016.08.300.
43. Dzubur, A.; Mekic, M.; Pesto, S.; Nabil, N. Echocardiographic Parameters as Life Quality Predictors in Patients After Myocardial Infarction Treated with Different Methods. *Med. Arch.* **2016**, *70*, 419, doi:10.5455/medarh.2016.70.419-424.
44. Mahesh, P.K.B.; Gunathunga, M.W.; Jayasinghe, S.; Arnold, S.M.; Haniffa, R.; De Silva, A.P. Pre-Event Quality of Life and Its Influence on the Post-Event Quality of Life among Patients with ST Elevation and Non-ST Elevation Myocardial Infarctions of a Premier Province of Sri Lanka. *Health Qual. Life Outcomes* **2017**, *15*, 154, doi:10.1186/s12955-017-0730-9.
45. Kang, K.; Gholizadeh, L.; Han, H.-R.; Inglis, S.C. Predictors of Health-Related Quality of Life in Korean Patients with Myocardial Infarction: A Longitudinal Observational Study. *Heart Lung* **2018**, *47*, 142–148, doi:10.1016/j.hrtlng.2017.12.005.

46. Xia, K.; Wang, L.-F.; Yang, X.-C.; Jiang, H.-Y.; Zhang, L.-J.; Yao, D.-K.; Hu, D.-Y.; Ding, R.-J. Comparing the Effects of Depression, Anxiety, and Comorbidity on Quality-of-Life, Adverse Outcomes, and Medical Expenditure in Chinese Patients with Acute Coronary Syndrome. *Chin. Med. J. (Engl.)* **2019**, *132*, 1045–1052, doi:10.1097/CM9.0000000000000215.
47. Kang, K.; Gholizadeh, L.; Han, H.-R. Health-Related Quality of Life and Its Predictors in Korean Patients with Myocardial Infarction in the Acute Phase. *Clin. Nurs. Res.* **2021**, *30*, 161–170, doi:10.1177/1054773819894692.
48. Wulandari, D.; Ginanjar, A.S.; Purwono, U.; Purba, D. Marital Satisfaction, Anxiety, and Health-Related Quality of Life in Myocardial Infarction Patients. *J. Glob. Pharma Technol.* **2020**, *12*, 483-495.
49. Džubur, A.; Lisica, D.; Hodžić, E.; Begić, E.; Lepara, O.; Fajkić, A.; Gogić, E.; Ejubović, M. Relationship between Depression and Quality of Life after Myocardial Infarction. *Med. Glas.* **2022**, *19*, 0–0, doi:10.17392/1404-21.
50. Rasmussen, A.A.; Fridlund, B.; Nielsen, K.; Rasmussen, T.B.; Thrysoee, L.; Borregaard, B.; Thorup, C.B.; Berg, S.K.; Mols, R.E. Gender Differences in Patient-Reported Outcomes in Patients with Acute Myocardial Infarction. *Eur. J. Cardiovasc. Nurs.* **2022**, *21*, 772–781, doi:10.1093/eurjcn/zvac022.
51. Upadhyay, V.; Bhandari, S.S.; Rai, D.P.; Dutta, S.; García-Grau, P.; Vaddiparti, K. Improving Depression and Perceived Social Support Enhances Overall Quality of Life among Myocardial Infarction Survivors: Necessity for Integrating Mental Health Care into Cardiac Rehabilitation Programs. *Egypt. J. Neurol. Psychiatry Neurosurg.* **2022**, *58*, 87, doi:10.1186/s41983-022-00521-6.
52. Jlassi, O.; Omrane, A.; Ben Massoud, M.; Khalfallah, T.; Bouzgarrou, L.; Gamra, H. Determinants of Health-Related Quality of Life among Patients with Ischemic Heart Disease. *Health Syst.* **2024**, *13*, 322–331, doi:10.1080/20476965.2023.2275799.
53. Sauletzhanovna, T.A.; Mohammed, W.K.; Ahmed, A.S.; Mohammed, H.I.; Al-Hili, A.; Alnajar, M.J.; Naser, N.S.; Amr, E.F.; Mohsin, R.M. The Predictive Value of Depression and Anxiety on Protracted Cardiovascular Outcomes in Individuals with Acute Myocardial Infarction. *Int. J. Body Mind Cult.* **2024**, 64–75, doi:10.22122/ijbmc.v11isp.739.
54. Füller, D.; Andresen-Bundus, H.; Pagonas, N.; Jaehn, P.; Ukena, C.; Gödde, K.; Holmberg, C.; Ritter, O.; Sasko, B. Adverse Socioeconomic Factors Are Associated with a Widening Gap in One-Year Health-Related Quality of Life after Acute Myocardial Infarction. *Sci. Rep.* **2025**, *15*, 19791, doi:10.1038/s41598-025-04604-1.
55. Malm, D.; Mårtensson, J.; Årestedt, K. Sense of Coherence and Quality of Life in the Recovery of Women and Men with Myocardial Infarction: A 10-Year Follow-up Study. *Eur. J. Cardiovasc. Nurs.* **2025**, *24*, 631–639, doi:10.1093/eurjcn/zvaf028.
